# Molecular and cellular mechanisms of HIF prolyl hydroxylase inhibitors in clinical trials†
†Electronic supplementary information (ESI) available: Synthetic procedures; supplementary methods including NMR, OGFOD1/Tpa1a/OFD1/PHD2/FIH hydroxylation assays, cell culture and immunoblotting, RT-qPCR assays and cell viability assays; supplementary figures; supplementary tables. See DOI: 10.1039/c7sc02103h


**DOI:** 10.1039/c7sc02103h

**Published:** 2017-09-11

**Authors:** Tzu-Lan Yeh, Thomas M. Leissing, Martine I. Abboud, Cyrille C. Thinnes, Onur Atasoylu, James P. Holt-Martyn, Dong Zhang, Anthony Tumber, Kerstin Lippl, Christopher T. Lohans, Ivanhoe K. H. Leung, Helen Morcrette, Ian J. Clifton, Timothy D. W. Claridge, Akane Kawamura, Emily Flashman, Xin Lu, Peter J. Ratcliffe, Rasheduzzaman Chowdhury, Christopher W. Pugh, Christopher J. Schofield

**Affiliations:** a Chemistry Research Laboratory , Department of Chemistry , University of Oxford , Oxford OX1 3TA , UK . Email: christopher.schofield@chem.ox.ac.uk; b Ludwig Institute for Cancer Research , Nuffield Department of Clinical Medicine , University of Oxford , Oxford OX3 7DQ , UK; c Structural Genomics Consortium (SGC) , University of Oxford , Oxford OX3 7DQ , UK; d Target Discovery Institute (TDI) , Nuffield Department of Medicine , University of Oxford , NDMRB Roosevelt Drive , Oxford OX3 7FZ , UK; e Radcliffe Department of Medicine , Division of Cardiovascular Medicine , BHF Centre of Research Excellence , Wellcome Trust Centre for Human Genetics , Roosevelt Drive , Oxford OX3 7BN , UK; f The Francis Crick Institute , 1 Midland Road , London NW1 1AT , UK

## Abstract

Inhibition of the human 2-oxoglutarate (2OG) dependent hypoxia inducible factor (HIF) prolyl hydroxylases (human PHD1–3) causes upregulation of HIF, thus promoting erythropoiesis and is therefore of therapeutic interest. We describe cellular, biophysical, and biochemical studies comparing four PHD inhibitors currently in clinical trials for anaemia treatment, that describe their mechanisms of action, potency against isolated enzymes and in cells, and selectivities *versus* representatives of other human 2OG oxygenase subfamilies. The ‘clinical’ PHD inhibitors are potent inhibitors of PHD catalyzed hydroxylation of the HIF-α oxygen dependent degradation domains (ODDs), and selective against most, but not all, representatives of other human 2OG dependent dioxygenase subfamilies. Crystallographic and NMR studies provide insights into the different active site binding modes of the inhibitors. Cell-based results reveal the inhibitors have similar effects on the upregulation of HIF target genes, but differ in the kinetics of their effects and in extent of inhibition of hydroxylation of the N- and C-terminal ODDs; the latter differences correlate with the biophysical observations.

## Abstract 2

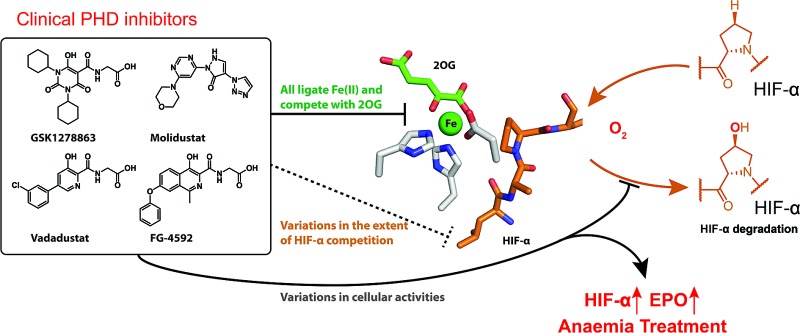
Four compounds in clinical trials for anaemia treatment are potent inhibitors of the hypoxia inducible factor (HIF) prolyl hydroxylases (PHDs), but differ in potency and how they interact with HIF at the PHD active site.

## Introduction

In humans, and other animals, the chronic response to hypoxia is regulated by the hypoxia inducible factors (HIFs), which are α,β-heterodimeric transcription factors.^1^ The HIF system works to enable cells, tissues, and whole organisms to adapt to limiting oxygen availability by upregulating an array of genes.^1^,^2^ The transcriptional activity of the HIFs is regulated in an oxygen dependent manner by 2-oxoglutarate (2OG) and ferrous iron dependent oxygenases which catalyze the post-translational hydroxylation of HIF-α subunits.^3^–^6^

HIF-α isoform prolyl-hydroxylation signals for degradation *via* the ubiquitin-proteasome system, because prolyl-hydroxylated HIF-α binds much more tightly than unmodified HIF-α to the Von Hippel–Lindau protein (pVHL), which is a targeting component of an E3 ubiquitin ligase complex.^4^,^7^,^8^ Two proline-residues, Pro402 and Pro564 in human HIF-1α, in the N- and C-terminal regions of the oxygen-dependent degradation domains, termed NODD and CODD, respectively, of HIF-α isoforms are efficiently hydroxylated by the HIF prolyl hydroxylases (PHDs or EGLNs) under normoxic conditions.^9^ Under hypoxic conditions, the activity of PHDs is limited by oxygen availability, so enabling the PHDs to act as hypoxia sensors.

In a second 2OG oxygenase-mediated mechanism of HIF regulation, factor inhibiting HIF (FIH) regulates HIF transcriptional activity *via* 2OG dependent hydroxylation of an asparagine-residue in the C-terminal transcriptional activation domain (CTAD) of HIF-α isoforms; such reaction reduces binding of HIF to transcriptional co-activator proteins (CBP/p300), which are histone lysine acetyltransferases.^10^,^11^ In humans there are three HIF-α isoforms of which HIF-1α and HIF-2α are most important. HIF works to upregulate transcription of hundreds of genes, the sets of which are context-dependent.^1^ HIF target genes include those encoding for proteins of biomedical interest, such as vascular endothelial growth factor (VEGF), nitric oxide synthase (NOS), and erythropoietin (EPO).^1^,^2^ The latter is of therapeutic interest because recombinant EPO is used for anaemia treatment. PHD inhibitors upregulate HIF-α and hence EPO (and other HIF target genes),^9^,^12^–^16^ and companies are pursuing PHD inhibitors for treatment of anaemia and other hypoxia related diseases.^17^–^25^ Four PHD inhibitors are currently in clinical trials for anaemia treatment.^18^,^25^

Given the pleiotropic and complex nature of the hypoxic response and the large number of components involved in the HIF system, it is likely important that clinically used PHD inhibitors are as selective as is possible for the desired physiological outcome, especially with regard to the long-term treatment of chronic diseases such as anaemia. There are ∼60 human 2OG oxygenases, which play roles in the regulation of protein biosynthesis, nucleic acid repair, collagen biosynthesis and fatty acid metabolism. Thus, off target inhibition by PHD inhibitors of other 2OG oxygenases may well be undesirable.^26^,^27^

Selectivity is also of interest with respect to HIF-1α and HIF-2α because the two HIF-α isoforms regulate substantially different, though sometimes overlapping, HIF target gene sets. For example, whereas carbonic anhydrase IX (*CA9*) is upregulated by HIF-1α,^15^ hepatic EPO is primarily regulated by HIF-2α.^28^ Moreover, given the differential specificity of the three PHD isoforms for the two prolyl-hydroxylation sites (NODD, Pro402, and CODD, Pro564) in HIF-1α,^29^ information on NODD/CODD inhibition may help enable development of PHD isoform or ODD specific inhibitors. Given that the role of FIH in HIF target gene expression varies in a context dependent manner,^30^ the selectivity of PHD inhibitors with respect to FIH is also important.

Here we report studies that inform on the activities, mechanisms of action, and selectivities of the PHD inhibitors (and related compounds) in clinical trials for anaemia treatment, *i.e.* Vadadustat from Akebia Therapeutics currently in phase III, FG-4592 from FibroGen in phase III, GSK1278863 from GlaxoSmithKline in phase III, and Molidustat from Bayer in phase II.^25^ We hope that the results be useful in interpreting the results of clinical trials with the compounds, and in future work on the therapeutic manipulation of the natural hypoxic response.

## Experimental

### Compound synthesis

FG-4592 was from Selleck Chemicals. IOX-4 was synthesized according to the reported procedure.^31^ GSK1278863, Vadadustat, and Molidustat were synthesized as described in the ESI.†

### X-ray crystallography

Recombinant forms of FIH (full-length) and PHD2 (residues 181–426) were produced as described.^10^,^32^ For crystallisation, Zn(ii) and Mn(ii) were substituted for Fe(ii) to avoid catalysis/reduce metal oxidation. Crystals were cryo-protected by transfer into crystallisation buffer supplemented with 20% (FIH) or 25% glycerol (PHD2) and freeze–cooled by plunging into liquid N_2_. Data for ligand bound protein complexes were from single crystals at 100 K using Diamond MX beamlines (see Table S1†). Data for FIH without inhibitor (apo-FIH) were collected from single crystals using ESRF ID30A-1/MASSIF-1 beamline. In total 8 apo-FIH datasets were averaged for the Pan-Dataset Density Analysis (PANDDA)^33^ for modelling Vadadustat. Data were processed using MOSFLM^34^ and SCALA^35^ for FIH and HKL2000 36 for PHD2. Structures were solved using Phaser^37^ using 1H2K (for FIH)^38^ and 4BQX (for PHD2)^39^ as search models. Alternating cycles of refinements using PHENIX^40^ and REFMAC^41^/CNS^42^ and model building using COOT^43^ were performed until *R*_work_ and *R*_free_ converged.

### MALDI-TOF MS PHD1, PHD2 and PHD3 hydroxylation and AlphaScreen PHD2 and histone demethylase assays

AlphaScreen antibody based PHD assays were as reported^39^ using 384-well white ProxiPlates™ (PerkinElmer). Reactions were performed in 50 mM HEPES buffer pH 7.5, 0.01% Tween-20 and 0.1% BSA in a final volume of 10 μL at room temperature. A 5 μL mixture of 10 nM PHD2 (catalytic domain, residues 181–426), 20 μM Fe(ii), and 200 μM l-ascorbic acid was incubated with 1 μL inhibitors supplemented with 20% DMSO for 15 minutes prior to incubation (10 minutes) with a 4 μL substrate mixture (150 nM biotinylated CODD peptide (HIF-1α residue 556–574) and 5 μM 2OG). Fianl concentrations of the reaction components: 5 nM PHD2, 10 μM Fe(ii), 100 μM l-ascorbic acid, 60 nM biotinylated CODD peptide, 2 μM 2OG and inhibitors with 2% DMSO. Reagent solutions as reported were used.^39^ The reaction mixture was quenched with 5 μL 30 mM ethylenediaminetetraacetic acid (EDTA). 5 μL of pre-incubated donor–acceptor bead mix (AlphaScreen® streptavidin-conjugated donor and ProteinA-conjugated acceptor beads; PerkinElmer) with HIF-1α hydroxy-Pro546 antibody (3434S, Cell Signaling) were then added to the reaction mixture for 1 hour in the dark at room temperature. The luminescence signal was measured using an Envision (Perkin Elmer) plate reader. Data were analyzed utilizing GraphPad prism.

Protocols for *in vitro* PHD1–3 MALDI-TOF MS hydroxylation assays^30^ and for the AlphaScreen histone demethylase assays^44^ were as reported.

### HRE reporter assays

HT1080 cells were stably transfected with a construct containing a firefly luciferase gene under the control of 5 tandem copies of the sequence: TCTAGAGGGCCCTACGTGCTGCTGCCTCGCATGGACTAGT (which contains the hypoxia response element (HRE) 3′ to the mouse erythropoietin gene) linked to a ‘minimal’ SV40 promoter. Clone P8 was selected on the basis of modest normoxic luciferase activity and significant (∼30 fold) induction by hypoxia, the 2OG oxygenase inhibitor dimethyloxaloylglycine (DMOG) or the iron chelator desferrioxamine (DFO) which remained stable over multiple passages.

HT1080P8 cells (25 000–30 000) were seeded into 96-well plates (100 μL per well) overnight to enable cell adhesion. The cells were then treated with 50 μL prepared 3× concentrated compound stock solution (supplemented with 1% DMSO final concentration in cell culture media) for the specified 6 hours, 16 hours and 24 hours (Fig. 3A and B). The medium was removed and cells washed with PBS. Luciferase signal was measured using Luciferase Assay System kit from Promega (E1500) following the manufacturer's protocol with a FLUOstar Omega Microplate Reader (BMG Labtech).

## Results and discussion

### Potency of the prolyl hydroxylase inhibitors

**Fig. 1:**
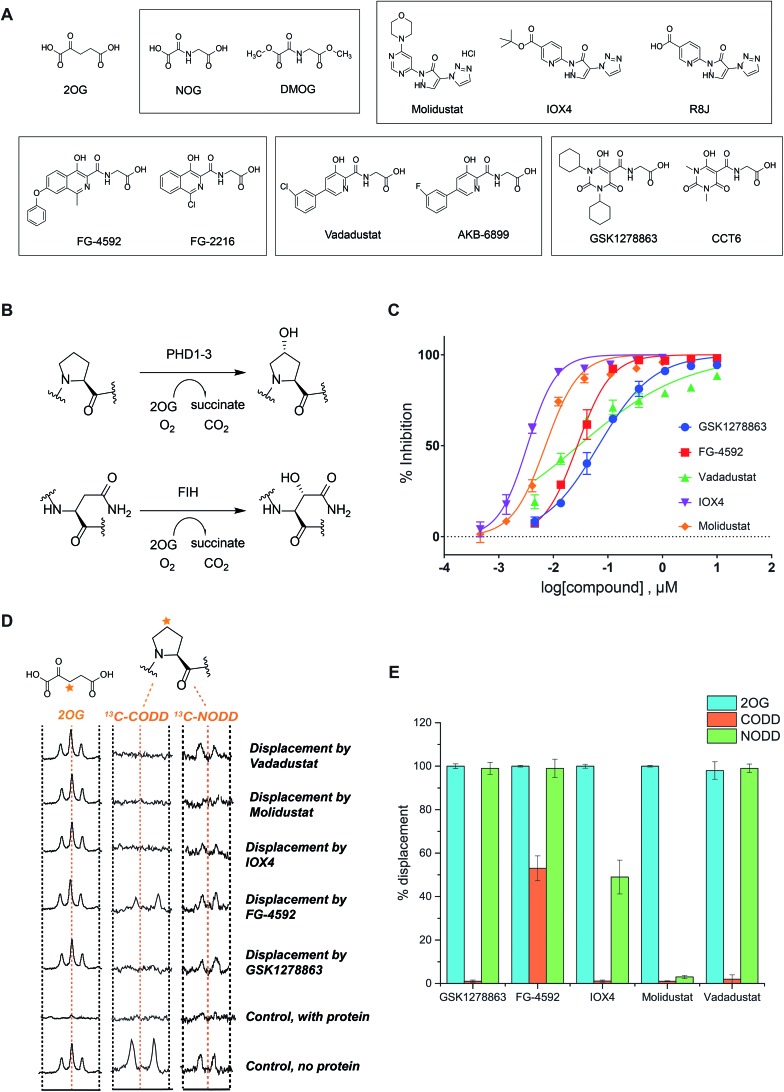
*In vitro* characterization of PHD inhibitors in clinical trials. (A) Chemical structures of 2OG, NOG, DMOG, FG-4592 and its structurally related analogue FG-2216, GSK1278863 and its analogue CCT6, Molidustat and its analogues IOX4 and RJ8, and Vadadustat, and the structurally related compound AKB-6899. (B) HIF-α hydroxylation reactions: PHD catalyzed prolyl- and FIH catalyzed asparaginyl-hydroxylation. (C) PHD inhibitor potency as assayed by the antibody based AlphaScreen assay.^39^ No enzyme and DMSO controls were used for normalization of the HIF-1α CODD peptide hydroxylation. Errors are standard deviations of the mean, *n* = 3. (D) Investigation of the binding mode of the PHD inhibitors by NMR.^56^,^63^ Qualitative single concentration screening of PHD inhibitors as monitored by CPMG-edited ^1^H analyses for 2OG displacement, and by 1D CLIP HSQC NMR with selective ^13^C-inversion analyses for ^13^C-CODD or ^13^C-NODD displacement. (E) Percentage of observed NODD/CODD displacement caused by the addition of PHD inhibitors to PHD2.Zn(ii).2OG. ^13^CODD or ^13^NODD complexes.

We began by evaluating the potency of the PHD inhibitors in trials against PHD2 (Fig. 1A), likely the most important of the three human PHD isoforms from a physiological perspective, and for which most structural information is available.^14^,^31^,^32^,^39^,^45^–^48^ Three of the PHD inhibitors in clinical trials, GSK1278863 (a.k.a Daprodustat),^49^,^50^ Vadadustat^51^ and Molidustat^52^,^53^ were synthesized as reported. FG-4592 (a.k.a. Roxadustat) was from Selleck Chemicals. For comparison, we included IOX4, a potent and selective PHD inhibitor, which is structurally related to Molidustat.^31^

To assess potency, we initially used a hydroxy-proline antibody-based AlphaScreen (Amplified Luminescent Proximity Homogeneous Assay Screen) assay to measure HIF-1α peptide (HIF-1α residue 556–574) hydroxylation as catalyzed by PHD2.^39^,^54^ The HIF-1α peptide used corresponds to the HIF-1α C-terminal oxygen-dependent degradation domain (CODD), the most conserved of the two prolyl-hydroxylation sites in HIF-1α and HIF-2α.^55^Fig. 1C shows dose–response curves for the five inhibitors. Indeed, like IOX4,^31^ all of the ‘clinical’ inhibitors potently inhibit PHD2 in this assay with IC_50_ values in the sub-μM range (Table 1). It is notable that Molidustat (IC_50_ = 7 nM) and the structurally related compound IOX4 (IC_50_ = 3 nM) are more potent than FG-4592 (IC_50_ = 27 nM), Vadadustat (IC_50_ = 29 nM) and GSK1278863 (IC_50_ = 67 nM) by this assay. By contrast, when using a LC-MS based assay, a different rank order of potency, with GSK1278863 being the most potent inhibitor, was obtained (Table S4†). Thus, the results reveal all of the compounds are potent PHD inhibitors, but imply variations in their mechanisms as manifested in assay dependent differences in relative potency. To investigate further, we initiated biophysical studies.

**Table 1:** Selectivity of FG-4592, GSK1278863, Molidustat, IOX4, and Vadadustat against isolated recombinant forms of human of 2OG dependent oxygenases (note that in some cases catalytic domains were used)^*a*^

Inhibitor	IC_50_ [μM]									
	PHD2	FIH	Ofd1	Tpa1p	OGFOD1	JARID1A (KDM5A)	JARID1B (KDM5B)	JARID1C (KDM5C)	JARID1D (KDM5D)	JMJD3 (KDM6B)
FG-4592	0.027	>100	8.5	12.8	<1	>100	>100	>100	>100	>100
GSK1278863	0.067	21	23.9	5.1	2.1	>100	>100	>100	>100	>100
Molidustat	0.007	66	10.3	3.8	5.1	>100	>100	>100	>100	35
IOX4	0.003	31	41.3	2.3	<1	53	87	97	91	<1
Vadadustat	0.029	29	ND^*b*^	ND^*b*^	1.4	>100	30	>100	>100	37

^*a*^Assay conditions are described in the Experimental and ESI sections and have been previously reported.^31^,^39^,^44^,^66^ PHD2: HIF-prolyl hydroxylase-2, FIH: factor inhibiting HIF, OGFOD1: 2OG and iron-dependent oxygenase domain containing 1, Ofd1: 2OG and Fe(ii) dioxygenase domain containing protein 1, Tpa1p: termination and polyadenylation protein 1, JARID1A (KDM5A): lysine-specific demethylase 5A, JARID1B (KDM5B): lysine-specific demethylase 5B JARID1C (KDM5C): lysine-specific demethylase 5C, JARID1D (KDM5D): lysine-specific demethylase 5D, JMJD3 (KDM6B): lysine-specific demethylase 6B.

^*b*^ND, not determined.

### Crystallography

**Fig. 2:**
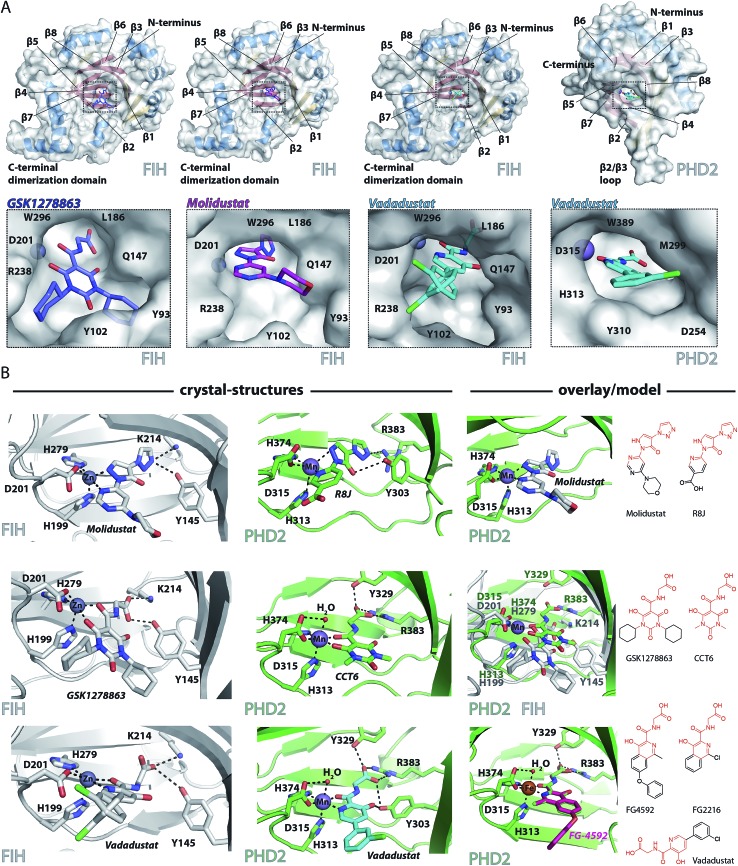
Crystallographically observed binding modes of clinically used PHD inhibitors with 2OG oxygenases. (A) Views from crystal structures of GSK1278863, Molidustat, and Vadadustat complexed with FIH; and Vadadustat complexed with PHD2. (B) Top: views from structures of: FIH complexed with Zn and Molidustat; PHD2_181–426_ complexed with Mn and the IOX4 derivative R8J (PDB code ; 5A3U)^31^; model of the PHD2.Mn.Molidustat complex. Middle: views from a structure of FIH complexed with Zn and GSK1278863; a structure of PHD2_181–426_ complexed with Mn and the GSK1278863 derivative CCT6; overlay of the structures from FIH.Zn.GSK1278863 and PHD2.Mn.CCT6. Bottom: views from structures of FIH in complex with Zn and Vadadustat; PHD2_181–426_ with Mn and Vadadustat; and an overlay of FG-4592 (modelled) with FG-2216 (using PDB code ; 3HQU^32^).

To date, there are no reported structures for the PHD inhibitors in clinical trials complexed with a 2OG oxygenase. Structures for the PHD2 catalytic domain complexed with related inhibitors, including R8J (related to Molidustat and IOX4, Fig. 2B)^31^ and FG-2216 (related to FG-4592, Fig. 2B),^32^,^39^,^45^ indicate the likely overall binding modes of Molidustat and FG-4592. The binding mode of GSK1278863 and related compounds (and to a lesser extent, Vadadustat) to 2OG oxygenases is of interest since it has a different scaffold to the FG-2216 related inhibitors. Although we did not obtain structures for three of the four clinical compounds with PHD2, we did obtain a structure of Vadadustat in complex with PHD2. We also obtained structures for GSK1278863, Molidustat and Vadadustat complexed with FIH, and a PHD2 structure with CCT6 (IC_50_ = 2.6 μM against PHD2, using AlphaScreen), which is closely related in structure to GSK1278863 (Fig. 1A). Our inability to obtain PHD2 structures with some inhibitors may reflect conformational changes induced by ligand binding (see below).^45^,^56^ Interestingly, during the course of the crystallization work, we accrued results implying that the conformational heterogeneity induced by small molecule binding to PHD2, can impede the potential for crystallographically productive packing, as has been reported,^31^,^32^,^39^ even with molecules that bind to form stable complexes in solution, and as supported by data not shown.

2OG oxygenase catalysis normally proceeds *via* 2OG binding to the iron (which is bound by a conserved HxD···H motif) followed by binding of substrate then oxygen (Fig. S1†).^57^ The 2OG C-5 carboxylate binds to basic and alcohol groups from subfamily specific residues, *i.e.* Arg-383 and Tyr-329 in PHD2, and Lys-214 and Tyr-145 in FIH.^38^,^45^,^58^ 2OG binds to the active site iron in a bidentate manner with its C-2 ketone oxygen coordinating at a conserved position (*trans* to Asp-315 for PHD2; *trans* to Asp-201 for FIH). The coordination position of the 2OG C-1 carboxylate varies, at least in the crystalline state. In the deacetoxycephalosporin-C synthase (DAOCS) subfamily, of which the PHDs are members, the 2OG C-1 carboxylate (normally) coordinates *trans* to the distal (*i.e.* C-terminal) iron-chelating histidine, whereas for Jumonji C (JmjC) subfamily members (of which FIH is a member) it is (normally) *trans* to the proximal (*i.e.* N-terminal) histidine of the HxD···H motif. The position of 2OG C-1 carboxylate binding is proposed to have mechanistic consequences, including for the relative rate of reaction of the enzyme-2OG substrate complex with oxygen, which is unusually slow for PHD2.^48^,^59^,^60^

FG-4592 is structurally related to Vadadustat and FG-2216; all three share the same glycinamide sidechain and have a similar heteroaromatic core (Fig. 1A). Structures for PHD2.Fe.FG-2216 reveal coordination of the metal *via* the glycinamide oxygen (*trans* to PHD2 Asp-315) and isoquinoline nitrogen (*trans* to PHD2 His-374) ring (Fig. 2).^32^ The glycinamide carboxylate is positioned to form electrostatic interactions with Tyr-329 and Arg-383 in PHD2, similarly to the 2OG C-5 carboxylate. The structure of Vadadustat complexed with PHD2 (1.99 Å resolution, *P*6_3_ space group, 1 molecule per asymmetric unit) shows a similar binding mode involving metal coordination *via* its glycinamide oxygen (*trans* to PHD2 Asp-315) and the nitrogen of its pyridine ring (*trans* to PHD2 His-374) and electrostatic interactions with Tyr-329 and Arg-383 with the glycinamide carboxylate (Fig. 2). The chlorophenyl group of Vadadustat projects into the substrate binding region and thereby likely interferes with productive substrate binding. However, the average *B* factor for the chlorophenyl group (48.0 Å^2^) is higher than that for the whole molecule (33.0 Å^2^), implying the chlorophenyl ring likely has a rotational freedom around the C–C axis that connects the two aromatic rings. Overall, the degree of similarity in active site binding modes observed in the PHD2-FG-2216 and PHD2-Vadadustat structures suggests conservation of interactions between PHD2 active site and these structurally related inhibitors. Given this structural/binding mode similarity, a model for FG-4592 binding PHD2 was generated (Fig. 2B) using the PHD2.Mn.FG-2216 crystal structure. Similarly to Vadadustat binding, the model predicts the hydrophobic phenoxy group of FG-4592, which is absent in FG-2216 (Fig. 2B), projects into the substrate binding region and will interfere with substrate binding (as supported by NMR studies – see below).

A structure of Vadadustat complexed with FIH (2.30 Å resolution, *P*4_1_2_1_2 space group, 1 molecule per asymmetric unit) reveals a similar coordination mode to that observed for FG-2216 and Vadadustat with PHD2, suggesting conserved binding of this motif. Note that the Vadadustat chlorophenyl group likely has multiple conformations in the FIH.Zn.Vadadustat structure; two major conformations were identified using Pan-Dataset Density Analysis (PANDDA, see ESI†).^33^ A crystal structure of Molidustat with FIH (2.30 Å resolution, *P*4_1_2_1_2 space group, 1 molecule per asymmetric unit) reveals binding to the metal *via* nitrogens of its pyrazolone (*trans* to Asp-201) and pyrimidine rings (*trans* to His-279); the triazole ring of Molidustat is positioned to form electrostatic interactions with the 2OG C-5 carboxylate binding residues (Fig. 2B). Although we did not obtain PHD2 crystals complexed with Molidustat, we have reported a structure with the related compound R8J, which is similar to IOX4 (Fig. 1A).^31^ R8J, has the same tricyclic core as Molidustat and coordinates the metal in PHD *via* the nitrogens of its pyrazolone ring (*trans* to Asp-315) and pyridine (*trans* to His-374) ring; its triazole ring is positioned to from electrostatic interactions with the 2OG binding residue Arg-383 (Fig. 2B), *i.e.* the triazole ring of Molidustat occupies the same site as the glycinamide side chain of FG-4592/FG-2216 (Fig. 2B). Surprisingly, analysis of Molidustat complexed with FIH (for which it is a poor inhibitor, see Table 1) reveals that the nitrogen of its pyrimidine ring is positioned to ligate to the metal *trans* to the distal histidine, as do R8J and 2OG in binding to PHD2 and not *trans* to the proximal histidine, as observed for FIH-2OG binding (Fig. S2†). Thus, the binding mode of Molidustat to FIH might in this respect reflect that of Molidustat to PHD2. A model of the PHD2.Mn.Molidustat complex based on overlay of FIH.Zn.Molidustat and PHD.Mn.R8J structures (Fig. 2B) indicates that the Molidustat morpholine ring may not completely block substrate binding, consistent with NMR studies (see below).

A structure of GSK1278863 (2.45 Å resolution, *P*4_1_2_1_2 space group, 1 molecule per asymmetric unit) with FIH reveals binding to the metal *via* its amide oxygen (*trans* to Asp-201), and one ‘pyrimidine-trione’ oxygen (*trans* to His-279); the GSK1278863 glycinamide carboxylate is positioned to form electrostatic interactions with Lys-214 and Tyr-145 (Fig. 2B). Interestingly, the oxygen of the pyrimidine-trione coordinates the metal *trans* to the distal histidine, as observed for Molidustat when complexed to FIH (Fig. 2B). Like GSK1278863, CCT6 has a pyrimidine-trione barbiturate ring core and glycinamide side chain. A structure of CCT6 (2.25 Å resolution, *P*6_3_ space group, 1 molecule per asymmetric unit) with PHD2 reveals metal coordination *via* its glycinamide oxygen (*trans* to Asp-315) and the oxygen of the pyrimidine-trione group (*trans* to His-374); the CCT6 glycinamide carboxylate is similarly positioned to form electrostatic interactions with the 2OG binding residues Tyr-329 and Arg-383. Overlay of the FIH.Zn.GSK1278863 and PHD2.Mn.CCT6 structures reveals an angular difference of 20° (degrees) between the binding mode of the pyrimidone rings of GSK1278863 and CCT6, likely in part due to steric demands of the cyclohexyl rings in GSK1278863 (Fig. 2).

Overall, the combined crystallographic studies predict the four clinical PHD inhibitors interact with the active site metal of the PHDs in a bidentate manner and bind to form electrostatic interactions in the 2OG C-5 carboxyl binding pocket. This binding mode enables them to compete with 2OG, consistent with work on related inhibitors.^31^ The differences in the extents to which the inhibitors project into the HIF-α substrate binding site of PHD2, and influence substrate binding, are notable (Fig. 2).

### NMR binding assays

To investigate the effect of the inhibitors on HIF binding, we used NMR which can reveal PHD binding modes not apparent from crystallography.^31^,^61^ We used the PHD2 catalytic domain (residues 181–426, with catalytically inert Zn(ii) substituting for Fe(ii))^48^ employing ^1^H excitation sculpting, ^1^H CPMG (Carr–Purcell–Meiboom–Gill), and wLOGSY (water-ligand observed *via* gradient spectroscopy) methods^62^ to investigate inhibitor binding.^63^,^64^ FG-4592, GSK1278863, Molidustat, and IOX4 all bind tightly (Fig. S6–S9†), with their *K*_D_ values in the sub-μM range as measured by either water relaxation or 2OG displacement^63^ (Fig. S10† and 1D), consistent with the catalytic assays. Using either water relaxation or 2OG displacement level assays, IOX4 was a weaker binder than GSK1278863, Molidustat or FG-4592. Note that these results contrast with the results of the AlphaScreen catalytic turnover assays, where IOX4 and Molidustat were more potent (Table 1).

Competition assays involving 2OG, NODD and CODD were carried out utilizing ^1^H CPMG^62^ and 1D selective HSQC NMR with selective ^13^C inversion.^65^ IOX4, FG-4592, GSK1278863, Molidustat, and Vadadustat completely displaced 2OG at 400 μM with PHD2 at 10 μM (Fig. 1D and E), implying 2OG competition, consistent with the crystal structures (Fig. 2). Different results were obtained when we examined the effects on the binding of ^13^C-labelled HIF-1α CODD and NODD. Only FG-4592 efficiently displaced CODD within detection limits (1D-selective HSQC with selective ^13^C inversion, Fig. 1E). This is notable since compounds closely related to FG-4592, such as FG-2216, do not, at least completely, displace CODD from PHD2 as revealed by both crystallography^32^,^45^ and NMR.^56^ The reason for displacement of CODD by FG-4592 likely, at least in part, results from the presence of its phenoxy group, which projects into the HIF-α substrate binding region (as based on modelling of the location of the phenoxy group of FG-4592, based on the crystal structure with FG-2216) adjacent to the catalytic centre so more efficiently displacing CODD. However, as shown by experiments with NODD, other factors must be involved. In contrast to the observations with CODD, GSK1278863, FG-4592, IOX4, and Vadadustat all clearly displaced NODD (Fig. 1E); by contrast the extent of NODD displacement by Molidustat was barely detectable.

Solution and crystallographic studies have revealed that binding of both CODD and NODD to the PHDs involves induced fit, in which a loop (β2β3 loop) folds to enclose the hydroxylated proline and the active site.^32^,^56^ Inhibitors not entirely displacing the ODD substrates (*i.e.* IOX4 and Molidustat with NODD, and all with CODD) thus likely manifest different active site conformations relative to those completely displacing substrate (*i.e.* GSK1278863, FG-4592, and Vadadustat on NODD). These differences may underline the different rank orders in inhibition/binding under the different assay conditions, as described above.

Overall, the combined biophysical analyses reveal that all of the clinical PHD inhibitors bind to the active site metal, compete with 2OG, but the extent to which they displace NODD or CODD for PHD2 is variable with Molidustat being notable in that it can bind without substantially displacing NODD or CODD and FG-4592 in that it most extensively displaced CODD.

### Selectivity against other 2OG dioxygenases

To investigate the selectivity of the PHD inhibitors, we tested them against representatives from different subfamilies, including FIH,^31^ 2OG and iron-dependent oxygenase domain containing 1 (OGFOD1),^66^ and members of the JmjC histone *N*^ε^-methyl lysine demethylase (KDM) subfamily,^44^ which are important in transcriptional regulation. It was important to include FIH, because the extent to which HIF target genes are regulated by FIH catalyzed HIF-α hydroxylation can vary and FIH has multiple non HIF-α substrates.^30^,^67^–^69^ OGFOD1 was included because it is also a human prolyl hydroxylase (acting at C-3 position, rather than C-4);^70^,^71^ previous studies have also shown that OGFOD1 may be inhibited by (some) PHD inhibitors.^66^

The results reveal that all of the tested compounds are selective for PHD2 over FIH, and almost all of the tested JmjC-domain containing histone demethylases (KDMs). One exception (as noted previously^31^) for IOX4, is that it, and to a much less extent, Molidustat, inhibit KDM6B/JMJD3 (Table 1). Notably, the selectivity for all of the compounds for PHD2 over the other tested prolyl hydroxylase, OGFOD1 (which has an active site closely related to PHD2),^66^ is substantially less than for the other oxygenases (Table 1, Fig. S12†). To investigate, we conducted tests with the PHD inhibitors against yeast homologues of OGFOD1, 2OG and Fe(ii) dioxygenase domain containing protein 1, Ofd1 (from *Schizosaccharomyces pombe*), and termination and polyadenylation protein 1, Tpa1p (from *Saccharomyces cerevisiae*).^70^ The results (Table 1, Fig. S14 and S15†) show all the compounds inhibit Ofd1 and Tpa1p with moderate potency, except for IOX4 which has a relatively high IC_50_ for Ofd1, which (in part) might be a consequence of the low solubility under the assay conditions. Thus, the clinical PHD inhibitors may not be (completely) selective with respect to other human prolyl hydroxylases, including the collagen prolyl hydroxylases.^6^

Although sharing homologous C-terminal catalytic domains, the N-terminal regions of PHD1–3 vary. Given the differences in their tissue distribution,^72^ subcellular localization^73^,^74^ as well as their activity on HIF hydroxylation of PHD1–3, it is of interest to know whether the PHD inhibitors in clinical trial shows selectivity for PHD isoforms. *In vitro* inhibition assays of the clinical trial PHD inhibitors (and some of their structurally related analogue) against 1 μM recombinant human PHD1–3 were performed. The results (Table S3 and Fig. S11†) show all inhibitors potently inhibit PHD1–3 with IC_50_ values around the single-digit micromolar or high sub-micromolar range.

### Cellular inhibition by the prolyl hydroxylase inhibitors

#### Inhibitor potency and HIF stabilization

To evaluate efficacy of the inhibitors in cells including for NODD/CODD hydroxylation selectivity, we performed luciferase-based hypoxia response element (HRE) reporter gene and immunoblotting assays. We tested whether the inhibition of the PHDs is effective through HIF-1α and -2α stabilization in HeLa, U2OS, Hep3b, HT1080 and RCC4 cells. This reporter system utilizes firefly luciferase expression as regulated by a promoter with a tandem HRE sequence.

**Fig. 3:**
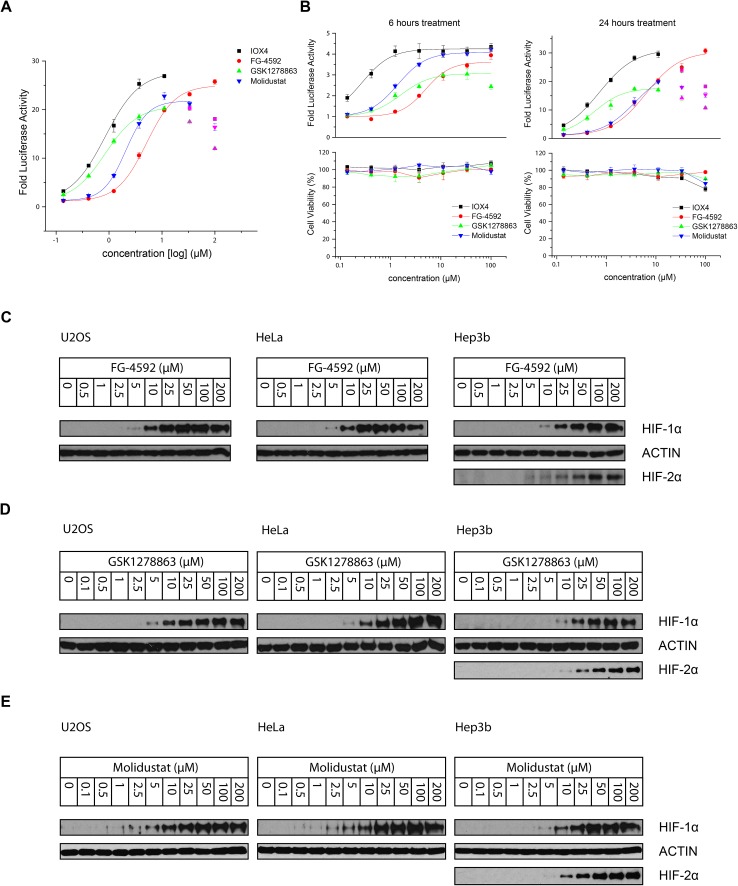
Stabilization of HIF-α through cellular inhibition of HIF prolyl hydroxylation by PHD inhibitors. (A) HIF-1α and HIF-2α induction in HT1080 cells 16 hours following PHD inhibitor treatment as measured by the HRE luciferase reporter assay. (B) Cell viability assays in parallel with an HRE reporter assay suggests the observed decrease of HIF induction with some inhibitor concentrations might, at least in part, be due to reduced cell proliferation. The apparent relative shift of the dose–response curves for GSK1278863 and Molidustat implies different characteristic pharmacodynamics for these two inhibitors. (C) Immunoblots showing dose-dependent HIF-1α stabilization in U2OS, HeLa and Hep3b (and HIF-2α) cells after 6 hours treatment with FG-4592, GSK1278863 (D) and Molidustat (E).

All the inhibitors potently induced firefly luciferase activity in a dose-dependent manner after 16 hours cell treatment with EC_50_ values of 0.8, 5.1, 0.8, and 2.1 μM being determined for IOX4, FG-4592, GSK1278863, and Molidustat, respectively (Fig. 3A). In the initial studies we observed Vadadustat to be substantially less potent than the other compounds; hence assays for it were conducted separately. Of all of the tested compounds, IOX4 and FG-4592 have highest efficacy on maximal level HIF stabilization, with a lower quantity of IOX4 being needed to achieve the same effect. Interestingly, although GSK1278863 has the lowest observed *E*_max_, it is more active than FG-4592 and Molidustat at low concentration ranges (Fig. 3A); the luciferase signals were reduced at relatively high (>33 μM) concentrations for GSK1278863, IOX4, and Molidustat.

To investigate whether some of the effects at high compound doses result from cytotoxicity, we carried out titrations over 6 and 24 hours (Fig. 3B). Good correlation between the decrease in the HIF induced luciferase signal and reduced number of viable cells with high dose treatment was observed for 24 hours of incubation. In contrast, no apparent decrease in number of viable cells or reduction in the luciferase signal was observed over 6 hours. The results suggest that the decreases in HIF luciferase activity at the high doses are, at least in part, due to reduced cell proliferation. Interestingly, the potencies of GSK1278863 and Molidustat appeared to change over time, with their titration curves shifting from 6 hours to 24 hours treatment relative to IOX4 and FG-4592. This observation may indicate different metabolic behaviours for GSK1278863 and Molidustat compared to IOX4 and FG-4592.

The cellular HIF-α stabilization was further studied using immunoblotting. Treatment with FG-4592 (Fig. 3C), GSK1278863 (Fig. 3D), or Molidustat (Fig. 3E) at up to 200 μM for 6 hours led to dose-dependent stabilization of HIF-1α in HeLa and U2OS cells, as well as HIF-2α in Hep3b cells (HeLa and U2OS cells express relatively low HIF-2α levels, Fig. S16†). In all of the cells tested, HIF-1α levels (and HIF-2α levels in Hep3b cells) were saturated at <100 μM FG-4592, GSK1278863 or Molidustat (Fig. 3C–E). Comparison of the three inhibitors in HKC8 and Hep3b cell lines on the same blot (Fig. S17A†) shows that the amount of compound required for saturating HIF-α levels with GSK1278863 is lower than with FG-4592 or Molidustat, consistent with the HRE results (Fig. 3A and B). Moreover, at 10 μM GSK1278863 the level of HIF-1α stabilization is higher than for FG-4592 or Molidustat, (Fig. S17A†), also consistent with the HRE results (Fig. 3A and B).

We performed titrations with Fe(ii) to investigate whether PHD inhibition involves iron chelation in solution, as for some compounds upregulating HIF-α, *e.g.* dipyridine (DP)^9^,^75^ and likely, desferrioxamine (DFO).^75^,^76^ Both HeLa and Hep3b cells were pre-treated with DMSO, DFO, IOX4, FG-4592, GSK1278863, or Molidustat (6 hours), followed by administration of ferrous ammonium citrate (FAC, for 20 hours). With DFO, HIF-1α upregulation is completely abolished by addition of ferrous iron, whereas with the other inhibitors, including DMOG, HIF-1α remained stable on iron addition (Fig. S17B†). These observations support the proposal that the clinical PHD inhibitors work by active site binding rather than by complexing iron in solution.

#### Inhibitor potency on HIF hydroxylation

**Fig. 4:**
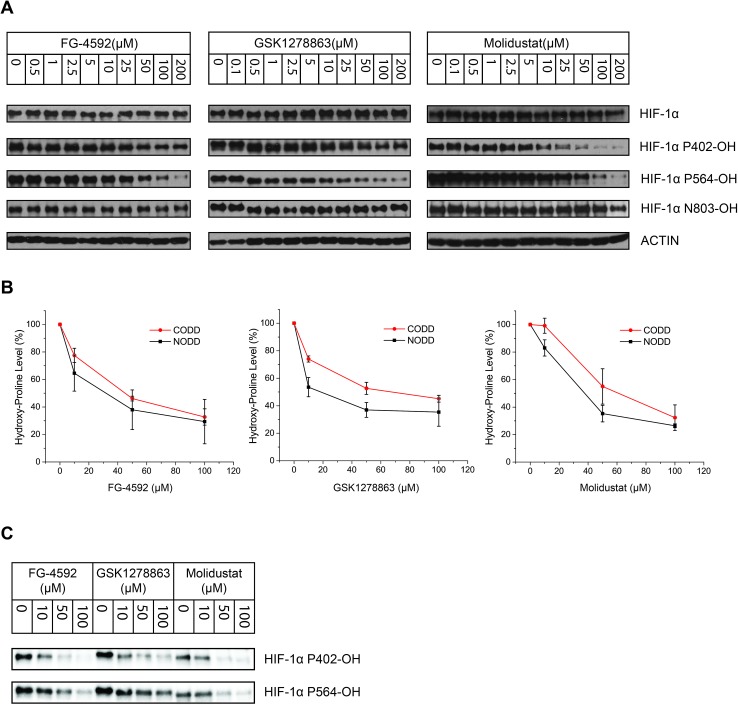
Cellular inhibition of HIF-1α prolyl- and asparaginyl-hydroxylation by the PHD inhibitors. (A) Immunoblots from human VHL-deficient RCC4 cells treated with the PHD inhibitors showing inhibition of both HIF-1α NODD and CODD hydroxylation. Titrations demonstrate dose-dependent inhibition. The lack of observed inhibition of Asn-hydroxylation up to 100 μM of the PHD inhibitors, implies they selectively inhibit Pro- over Asn-hydroxylation. (B) Comparison of inhibitor potency on CODD and NODD hydroxylation sites showing the means of values of for the three inhibitors from 3 independent experiments. Each data point represents the mean value and standard deviation of 3 independent biological (assay) repeats. Quantification of the immunoblots was done using Image Lab™ Software. (C) Representative blots of data used in (B).

To test whether the cellular HIF-α induction occurs *via* inhibition of HIF-α hydroxylation, we utilized a Von Hippel–Lindau (VHL)-deficient renal cell carcinoma cell line RCC4, in which HIF-α is constitutively stabilized in normoxia due to loss of functional VHL.^77^ We used antibodies specific for hydroxylation at the N-terminal and C-terminal oxygen-dependent degradation domains, Pro402 (NODD) and Pro564 (CODD) at HIF-1α.^78^ HIF-1α hydroxylation status was analyzed after 6 hours treatment. IOX4, FG-4592, GSK1278863, and Molidustat inhibited both NODD and CODD hydroxylation in a dose dependent manner (Fig. 4A). We also analyzed inhibition of Asn-hydroxylation (Asn803) in HIF-1α by immunoblotting.^78^,^79^ FG-4592, GSK1278863 and Molidustat all manifested no sign of HIF-1α Asn803 hydroxylation inhibition (at least up to 100 μM, Fig. 4A), consistent with the isolated protein assay results (IC_50_s for FIH are >100 fold higher than for PHD2, Table 1).

To investigate whether there is a difference in inhibition of hydroxylation at the NODD and CODD sites, we treated RCC4 cells with FG-4592, GSK1278863, or Molidustat at 10, 50 and 100 μM (6 hours). Notably, FG4692, which was the only inhibitor showing displacement of CODD by NMR (Fig. 1E) showed the greatest inhibition of CODD hydroxylation among the inhibitors in cells (Fig. 4B). Secondly, GSK1278863, which displaces NODD but not CODD in the isolated protein assays, better inhibits NODD than CODD hydroxylation in cells. Moreover, Molidustat, which did not displace NODD or CODD peptide, showed weaker inhibition of both hydroxylation sites compared to FG-4592 and GSK1278863 at 10 μM (Fig. 4B). Interestingly, although displacement of the substrate does not directly correlate with inhibition of hydroxylation (*e.g.* 2OG competition will cause inhibition even if the substrate is not displaced by inhibitors), these cellular results correlate with the NMR NODD/CODD displacement results (Fig. 1E).

#### Time dependent HIF stabilization with prolyl hydroxylase inhibitors

**Fig. 5:**
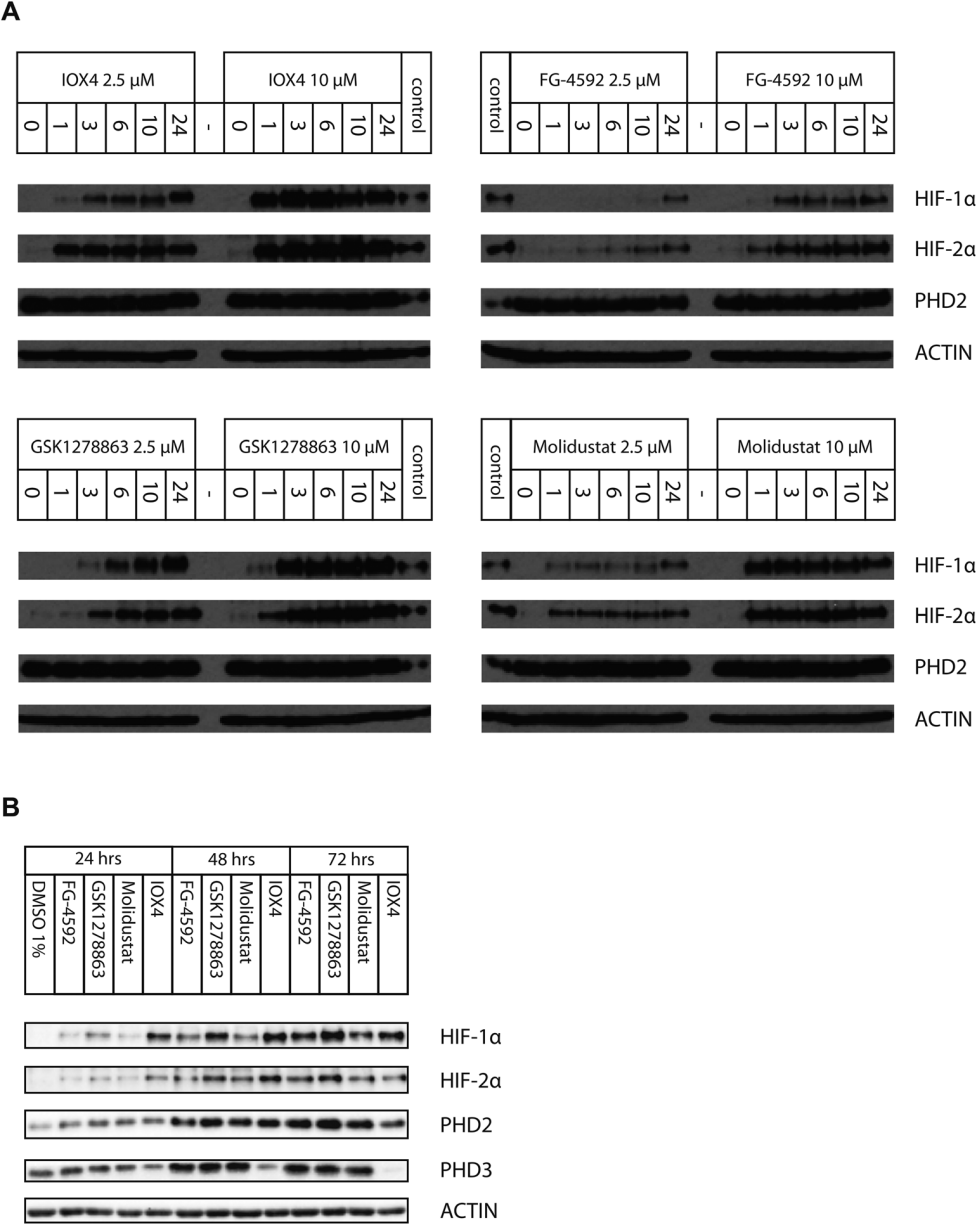
Time dependent HIF-α stabilization by PHD inhibitors. (A) Immunoblots showing HIF-α induction in Hep3b cells at the 1st, 3rd, 6th, 10th, and 24th hour after treatment of cells with FG-4592, GSK1278863, IOX4, or Molidustat at 2.5 μM and 10 μM. Samples including controls were loaded equally on the four blots. (B) Immunoblots showing HIF-α induction in Hep3b cells after prolonged treatment (24, 48, and 72 hours) with 10 μM FG-4592, GSK1278863, IOX4, or Molidustat.

We then investigated potential time dependent effects of the inhibitors on HIF-α stabilization in Hep3b cells (Fig. 5A). Both IOX4 and Molidustat showed differential HIF-1α, and HIF-2α stabilization. At 2.5 μM inhibitor concentration, the HIF-2α level was saturated after 1 hour, but the HIF-1α level continued to increase for up to 24 hours. At 10 μM IOX4 HIF-1α (HIF-2α) level saturated at 3–6 (3–10) hours and slightly decreased up to 24 hours; however, at 10 μM Molidustat saturated HIF-1α and HIF-2α levels after the first hour. FG-4592 showed the least effect on both HIF-1α and HIF-2α upregulation among the four inhibitors; the HIF-1α and HIF-2α levels gradually increased over 24 hours for both isoforms. For GSK1278863, treatment at 2.5 μM significantly increases HIF-1α levels over time; however, the increase in the HIF-2α level was not as sharp as for HIF-1α; at 10 μM both HIF-1α and HIF-2α level saturated after 3 hours of GSK1278863 treatment.

We then investigated inhibition over longer times in Hep3b cells (10 μM for 24, 48 and 72 hours), using immunoblotting for HIF-1α, HIF-2α, PHD2 and PHD3 (Fig. 5B). At 10 μM, IOX4 was most effective at stabilizing HIF-1α and HIF-2α after 24 hours treatment; the effects of GSK1278863 started to match those of IOX4 after 48 hours and exceeded for both HIF-1α and HIF-2α, after 72 hours. It is notable that the cell density at the 72nd hour for IOX4 was significantly lower than the other 3 inhibitors (40–50% less cell as judged visually). Both HIF-1α and HIF-2α levels increased gradually over 24–72 hours after treatment with 10 μM FG-4592. With 10 μM Molidustat treatment, the HIF-1α level gradually increased over 72 hours whereas HIF-2α levels were saturated after 48 hours. Two of the three human HIF prolyl hydroxylase isoforms, PHD2 and PHD3 (but not PHD1), are HIF target genes with PHD3 being negatively upregulated by hypoxia, a process that could act as a negative feedback loop.^80^–^85^ Upregulation of both PHD2 and PHD3 were observed after 48 and 72 hours with all inhibitors, except IOX4. It is possible that the reduction in PHD2 and PHD3 levels after 72 hours of IOX4 treatment is accounted for by low cell number. However, the strong reduction of PHD3 protein levels after 48 hours of IOX4 treatment could well be a consequence of an unidentified mechanism, suggesting possible off-target effects for IOX4.

#### Effect of the inhibitors on HIF target gene regulation

**Fig. 6:**
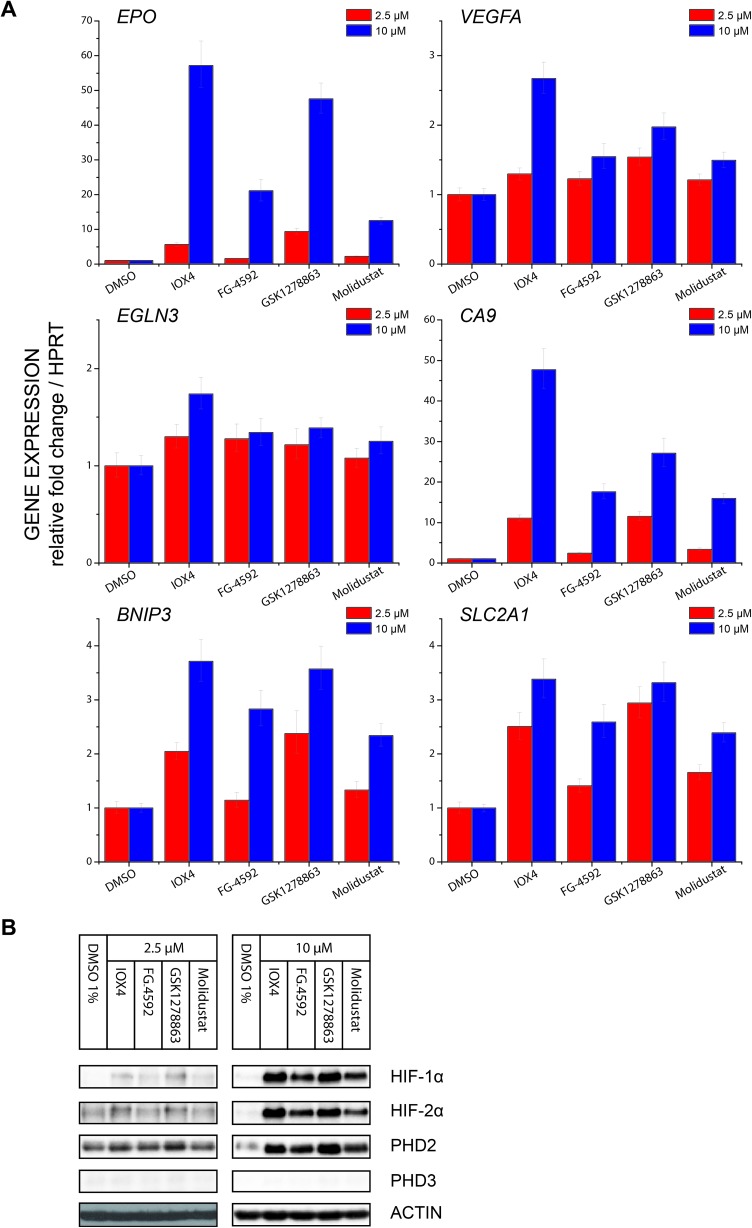
Effects of PHD inhibitors on HIF target gene regulation. (A) Regulation of HIF target genes, HIF-1α dependent carbonic anhydrase IX (*CA9*) and BCL2/adenovirus E1B 19 kDa protein-interacting protein 3 (*BNIP3*), HIF-2α dependent erythropoietin (EPO), both HIF-1α and HIF-2 α dependent glucose transporter 1 (*SLC2A1*) and vascular endothelial growth factor (*VEGFA*), and the strongly FIH dependent HIF target gene PHD3 (*EGLN3*), after treatment with 2.5 μM or 10 μM PHD inhibitors for 24 hours, as assayed by RT-qPCR. Each data point represents the mean and standard deviation of the relative fold change with respect to DMSO treated sample normalized to reference gene *HPRT* level, *n* = 3 biological repeats for inhibitors and *n* = 4 for DMSO control. (B) Immunoblots of cells treated with 2.5 μM and 10 μM PHD inhibitors for 24 hours showing level of HIF-1α, HIF-2α, PHD2 and PHD3 protein level at the 24th hour. Cells were seeded and treated with inhibitors at the same time of experiments in (A). Note that correlation between the HIF-α protein level and HIF-α target gene response is observed.

The upregulation of HIF-α isoforms has multiple downstream effects.^1^,^30^,^86^,^87^ To investigate whether the inhibitors have differential effects on HIF target genes we measured their mRNA levels for a set of HIF target genes after 24 hours in Hep3b cells. The panel aimed to cover a small, yet representative, HIF target gene set. It included HIF-1α regulated carbonic anhydrase IX (*CA9*) and BCL2/adenovirus E1B 19 kDa protein-interacting protein 3 (*BNIP3*), primarily HIF-2α regulated erythropoietin (*EPO*), both HIF-1α and HIF-2α regulated glucose transporter 1 (*SLC2A1*) and vascular endothelial growth factor (*VEGFA*) as well as the strongly FIH-dependent HIF target gene PHD3 (*EGLN3*).^30^ The changes in HIF target gene mRNA levels (Fig. 6A) correlate with HIF-1α and HIF-2α protein levels (Fig. 6B), with IOX4 and GSK1278863 causing the highest response consistent with their relatively high HIF-α protein stabilization. Amongst these genes, *CA9* and *EPO* responded most significantly to inhibitor treatment with highest fold change up to 50 folds at 10 μM and 10 fold at 2.5 μM; *BNIP3* and *SLC2A1* have relatively mild response to inhibitor treatment (up to 3 fold changes); the strongly FIH-dependent gene *PHD3* showed no clear sign of upregulation (PHD3 immunoblots showed no signed of induction on inhibitor treatment, likely due to the fact that FIH is not inhibited. See Fig. S19† for longer exposure). Interestingly, at 2.5 μM GSK1278863 resulted in slightly higher HIF protein level stabilization as well as target gene upregulation than IOX4 whereas at 10 μM reverse effect was observed. This is consistent with the immunoblotting and reporter results showing that GSK1278863 stabilizes HIF-1α more than other inhibitors at a relatively low inhibitor concentration (<10 μM in Fig. 3A and B and 10 μM in Fig. S17A†).

#### Cellular assays with Vadadustat

The effects of Vadadustat on inhibition of PHD activity in the cells tested were substantially weaker than the other inhibitors as demonstrated in multiple repeated assays. Fig. S20A† shows that the ability of Vadadustat (EC_50_ 41 μM for 16 hour treatment) on HIF-α induction in the HRE reporter assay in HT1080 cells is weaker than for FG-4592 (EC_50_ 7 μM for 16 hours treatment), and the other compounds tested above (*i.e.*, IOX4, GSK1278863, and Molidustat). Similar levels of differential activities between Vadadustat and FG-4592 were observed after 6 hour and 24 hour treatments.

Immunoblots of HIF-1α gave similar results to the HRE luciferase assay results. When Hep3b cells were treated with up to 200 μM Vadadustat for up to 6 hours, dose-dependent HIF-1α stabilization was not clear until 50 μM Vadadustat was used (Fig. S21A†). Further, unlike the other inhibitors (Fig. 3C–E), where the level of HIF-1α was saturated around 100 μM, the effect on HIF-1α increased with >100 μM Vadadustat. Direct comparison of HIF-1α induction after 6 hours treatment in Hep3b cells (Fig. S21B†) shows that the potency of Vadadustat is substantially weaker than FG-4592, at least in our cellular assays.

The preclinical candidate, AKB-6899, which is structurally related to Vadadustat (Fig. 1A and S21C†), is reported to selectively induce HIF-2α accumulation *via* preferential inhibition of PHD3,^88^ although dose-dependent stabilization of HIF-1α by AKB-6899 was also reported in another study.^89^ Our assays using isolated enzymes show AKB-6899 potently inhibits all three PHD isoforms (Fig. S10 and Table S3†). To investigate whether AKB-6899 selectively induces HIF-2α, we treated Hep3b cells with Vadadustat, AKB-6899 and FG-4592 at various concentrations (for 6 hours). We observed that AKB-6899 stabilizes HIF-1α, but to a much lesser extent than FG-4592 (Fig. S21C†). Interestingly, the effect of Vadadustat and AKB-6689 on HIF-2α stabilization was at a more comparable level to that of FG-4592, suggesting an apparently potential preferential effect of Vadadustat and AKB-6689 on HIF-2α compared to HIF-1α (as compared to FG-4592) in Hep3b cells. Thus, although our assays with isolated enzymes show Vadadustat/AKB-6689 potently inhibit PHD1–3, they may manifest selective inhibition in cells.

The potency of inhibition of HIF-1α ODD proline hydroxylation was analyzed after 6 hours treatment in RCC4 VHL deficient cells with Vadadustat. Similar to HIF-1α stabilization, Vadadustat was not very potent in inhibiting HIF-1α NODD or CODD hydroxylation. Further, whereas, NODD hydroxylation was significantly inhibited with 50 μM FG-4592, no sign of PHD inhibition was observed with 50 μM Vadadustat, and only slight inhibition was observed at 100 μM Vadadustat (Fig. S21D†). Similarly, inhibition of CODD hydroxylation was also relatively weaker with Vadadustat: no clear inhibition with up to 100 μM Vadadustat was observed whilst clear inhibition was observed with 50 μM FG-4592. Inhibition of hydroxylation of Asn803 was not observed with up to 200 μM Vadadustat (Fig. S21D†).

To investigate whether the relatively low activity of Vadadustat in our assays is cell type dependent, we tested Vadadustat and FG-4592 at 10 and 100 μM for 7 hours in HeLa, 293T and HKC8 cells in parallel with testing in Hep3b cells. The resulting immunoblots (Fig. S22†) show that the apparent potency of the inhibitors indeed varies in different cell types, though Vadadustat was consistently less potent than FG-4592. The variations may, in part, be a consequence of variations in the expression levels of the three PHDs in different cell lines, as well as the differences in cellular uptake of the inhibitors.

The effect of Vadadustat on HIF-α target genes correlates well with its effect on HIF-α protein stabilization. Treatment with 2.5 or 10 μM Vadadustat of Hep3b cells for 24 hours failed to obviously upregulate assayed HIF target genes; by contrast, FG-4592 treatment at the same concentration showed similar levels of gene upregulation (Fig. S23A and B†) as before (Fig. 6). Upregulation of HIF target genes with Vadadustat for 24 hours was observed at higher concentrations (50 and 100 μM) though the effects were still smaller than analogous treatment with FG-4592 (Fig. S24†).

## Discussion

The discovery that EPO, and subsequently other hypoxically upregulated proteins, are regulated by HIF,^87^,^90^,^91^ the α-subunit of which is strongly hypoxically regulated, has promoted investigations on the pharmacological modulation of the HIF system, aimed at either activating or deactivating HIF/promoted expression. The most clinically advanced work concerns PHD inhibitors, four of which are now in trials for anaemia treatment *via* upregulation of EPO or selective metabolism.^18^,^25^ Various lines of evidence indicate that PHD inhibition is efficacious in terms of EPO upregulation. However, given the pleiotropic and context dependent activities of the different forms of HIF and the multitude of other variables involved in the regulation of transcription, it is uncertain how difficult it will be to achieve clinically acceptable PHD inhibitors. Although selectivity might be achieved *via* targeting inhibitors to specific organs, *e.g.* the kidneys and liver for EPO upregulation, studies comparing the *in vitro* properties of the compounds in clinical trials are likely important, in particular for long term drug use.

Overall, our results reveal that the PHD inhibitors in clinical trials, FG-4592, GSK1278863, Molidustat and Vadadustat, work by active site iron chelating mechanisms in terms of inhibiting isolated PHD2 and, that FG-4592, GSK1278863, Molidustat have similar potencies in cells. At least in our cellular assays, for reasons presently unknown (possibly relating to uptake/metabolism), Vadadustat was less potent than the other three inhibitors. Although factors other than cellular potency are likely involved *in vivo*, this difference appears to correlate with the higher doses of Vadadustat that are being used in clinical trials for anaemia treatment compared to the FG-4592, Molidustat and, in particular, GSK1278863 (Table S5†). There were clear differences between the inhibition kinetics of the compounds that are manifested in both turnover and binding assays using isolated recombinant enzymes; these differences may have consequences for the clinical application of the compounds, particularly over the long timeframes normally required for treatment of anaemia.

Catalytic assays revealed the clinical PHD inhibitors to potently inhibit PHD2 in the sub-micromolar range; Molidustat (and the related compound IOX4) were more potent than FG-4592 and Vadadustat, and GSK1278863 was a weaker inhibitor in an antibody-based assay. By contrast when using the more direct LC-MS based assay, GSK1278862 was the most potent inhibitor. NMR studies revealed all the compounds compete with 2OG; this observation is consistent with the crystallographic insights employing PHD2 and FIH using either the clinical inhibitors, or analogues more amenable to crystallization, which reveal all three bind to the active site metal in a bidentate manner. Importantly, the solution NMR studies reveal differences in the relative extents to which the inhibitors displace CODD and NODD binding to PHD2. Although all the inhibitors very likely perturb the catalytically productive conformation of HIF-α at the PHD active site,^39^ of the four inhibitors tested, only FG-4592 displaced CODD efficiently within our limits of detection using the semi-quantitative NMR assays (Fig. 1D and E). Previous work has shown that FG-2216, which is structurally related to FG-4592, does not completely displace CODD;^56^ this difference likely reflects the presence of an additional phenoxy group on FG-4592, which is predicted to project into the CODD/NODD binding site (Fig. 2B). However, the situation is more complex than simple steric blockade of substrate binding, since, *e.g.*, one of the cyclohexyl groups of GSK1278863 might be expected to act in a similar manner to the phenoxy group of FG-4592, and the rank order results of the NMR based binding assays in the absence of NODD/CODD does not mirror those of the AlphaScreen catalytic turnover assays (though it better correlates with the more direct LC-MS assay results). Further, the results of the NMR-based NODD binding assays reveal that FG-4592, GSK1278863, and IOX4 all displace NODD; however, NODD displacement was barely detectable for Molidustat. Overall, these results likely reflect the substantial induced fit nature of substrate binding to the PHDs.^56^ Importantly, they imply differences in the binding modes of NODD and CODD to the PHDs, at least when complexed with inhibitors. Given the emerging different roles for NODD/CODD and PHD/HIF-α isoforms, these observations may be of biological relevance.^56^ Differential sequestration of NODD/CODD may have consequences for the activities of inhibitors in cells, *e.g.* by limiting binding of HIF-α to pVHL, or by modulating PHD lifetime.

Selectivity profiling with representatives of other subgroups of human 2OG dependent oxygenases including FIH and JmjC histone demethylases (KDMs) indicates that all four tested inhibitors are selective, with the notable exception of OGFOD1 (and its yeast homologues Ofd1 and Tpa1p), which were assigned as prolyl hydroxylases subsequent to development of the clinical PHD inhibitors.^70^,^71^,^92^–^94^ This observation may reflect the similarity of the OGFOD1 active site to that of the PHDs.^66^ Like the PHDs, OGFOD1 is a prolyl hydroxylase, albeit one acting at the proline C-3 position.^71^ The lack of inhibition of FIH by the clinical PHD inhibitors is also notable (and consistent with cellular results), since the extent to which HIF target genes are regulated by FIH activity (and FIH inhibitors) varies.^30^ Overall, these results indicate that the PHD inhibitors in clinical trials are likely quite selective against other human 2OG oxygenases, but with an important exception being other prolyl hydroxylases. Further studies on the selectivity of the inhibitors *versus* other human prolyl hydroxylases, including the C-3 and C-4 prolyl hydroxylases involved in collagen biosynthesis are of interest.^95^–^97^

The results of HRE reporter assays in HT1080 cells reveal micromolar (FG-4592, Molidustat, and Vadadustat) or sub-micromolar (IOX4 and GSK1278863) potencies. Interestingly, these results appear to contrast with the immuno-based assay results with isolated PHD2 (though not with the NMR binding assay and LC-MS catalytic assay results) wherein GSK1278863 was ∼10 fold less potent than Molidustat. Moreover, the maximum dose induction of HIF-α, as characterized either by the HRE reporter assay or by immunoblotting, was consistently lower for GSK1278863 than the other inhibitors. These observations may reflect complexities in the precise PHD inhibition mechanism, as suggested by the results with isolated PHD2, in particular with respect to NODD/CODD competition, or cell penetration (inhibitor degradation issues). In the latter regard, it is of interest that GSK1278863 is reported to undergo P450 oxygenase mediated hydroxylation of its cyclohexane rings.^49^,^50^ It is possible that one, or more, of the hydroxylated metabolites may be in part responsible, for the cellular activity of GSK1278863.

Time-dependent studies with FG-4592, GSK1278863, Molidustat and IOX4 in Hep3b cells revealed different behaviours. For treatment within 24 hours, with 2.5 μM of either IOX4 or Molidustat, HIF-1α gradually stabilized up to 24 hours, but HIF-2α levels saturated after the first hour; with 10 μM IOX4 or Molidustat, both HIF-1α and HIF-2α levels saturated early after 3–6 and 1 hour respectively; at 2.5 μM and 10 μM FG-4592, both HIF-α isoforms level gradually increased over 24 hours; at 2.5 μM GSK1278863, HIF-1α levels increased substantially, whereas the increase in HIF-2α levels was smaller over the same time frame; at 2.5 μM GSK1278863, both HIF-1α and HIF-2α saturated after 3 hours (Fig. 5A). On prolonged treatment, IOX4 had the greatest effect on HIF-1α and HIF-2α after 48 hours, GSK1278863 was better after 72 hours; the lower level of upregulation of both HIF-α isoforms with IOX4 treatment may reflect its cytotoxicity (Fig. 5B and S18†). Although the assay timescales may not be relevant to the long timescales of drug treatment, the results suggest careful time-dependent monitoring of the consequence of inhibitor application should be carried out.

Profiling of a representative set of HIF-α target gene (*EPO*, *VEGFA*, *CA9*, *BNIP3*, *EGLN3* (*PHD3*), and *SLCA2A1*) responses in the presence of a moderate (effectively inducing HIF-α but not causing cytotoxicity) inhibitor concentration using RT-qPCR, revealed inhibition induced upregulation of gene expression in a dose dependent manner. Notably, the expression of *EGLN3* (*PHD3*), which is strongly FIH dependent,^30^ was marginally induced, consistent with the lack of activity of the inhibitors *versus* FIH. Moreover, none of IOX4, FG-4592, GSK1278863 or Molidustat showed differential HIF-1α *versus* HI-2α regulation (Fig. 6A and B).

Studies utilizing antibodies specific for NODD and CODD hydroxylation sites in human VHL-deficient RCC4 cells, which manifest stable HIF-α levels, validated the ability of the inhibitors to inhibit the PHDs in cells. Quantification of HIF-α hydroxylation levels in RCC4 cells reveals differential effects on NODD/CODD hydroxylation for different inhibitors (Fig. 4). Although multiple factors operate in cells, it is interesting that the observations on NODD/CODD hydroxylation correlate with the NMR NODD/CODD displacement results using purified PHD2, *i.e.* FG-4592, the only inhibitor that displaces CODD, shows most potent inhibition of CODD hydroxylation; GSK1278863, which only displaced NODD in NMR assay, showed better NODD inhibition over CODD in cells; and Molidustat, which does not displace NODD or CODD in solution, had a weaker effect on either hydroxylation site than FG-4592 or GSK1278863 in cells (at 10 μM).

Using a HIF-1α hydroxy-asparagine specific antibody^78^,^79^ immunoblotting of RCC4 cells treated with the PHD inhibitors showed no sign of FIH inhibition, consistent with the *in vitro* selectivity profiling results against FIH and expression profiling. Thus, the clinical PHD inhibitors will not only alter the ratio of ODD prolyl-hydroxylated: non prolyl-hydroxylated HIF-α isoforms (in the favour of the latter), but potentially also the ratio of CTAD asparaginyl-hydroxylated: CTAD non asparaginyl-hydroxylated HIF-α. Given that the extent to which FIH regulates HIF target genes varies (in a context dependent manner)^30^ and that the effect of CTAD asparaginyl-hydroxylation are mediated *via* reduced binding of the CTAD to the highly pleiotropic CBP/P300 transcriptional coactivator proteins, it is difficult to predict the physiological effect of perturbed CTAD hydroxylation status of HIF-α isoforms, but it is something that should be considered in the ongoing clinical and animal studies with PHD inhibitors.

## Conclusions

Overall the results reveal that the PHD inhibitors in clinical trials have similar overall mechanisms of action and of potencies, both in terms of assays in cells and against the isolated PHD enzymes. They all work *via* chelation to the active site iron, though note this mechanism does not necessarily preclude selectivity, as evidenced by assays against other 2OG dependent oxygenases. However, the selectivity results imply that further work on the optimizing selectivity of the inhibitors with respect to the other prolyl-hydroxylases may well be beneficial. There are, however, clear differences between the inhibitors, as manifested in the time dependencies of their effects in cells, and, in particular, in differences in their relative effects on NODD and CODD binding. The collective results suggest that the development of PHD inhibitors that manifest at least a degree of inhibition selectivity for NODD/CODD and maybe HIF-1α/HIF-2α hydroxylation should be possible. The latter is of particular interest given that it has been reported that HIF-1α and HIF-2α may have opposing roles on the progress of cancer.^98^ In this regard the development of inhibitors, including those not binding to the active site metal, that exploit differences in the binding of different HIF-α and non-HIF substrates to the PHDs is also of interest.

## Accession numbers

The atomic coordinates for the reported crystal structures have been deposited to the Protein Data Bank with PDB accession codes 5OP6, ; 5OP8, ; 5OPC, ; 5OX6, and ; 5OX5.

## Author contributions

T.-L. Y. designed and performed experiments including PHD2 AlphaScreen inhibition assay and cellular assays, analyzed data, prepared figures, and wrote the manuscript. T. M. L, I. J. C., and R. C. determined structures of FIH and PHD2 analyzed data, and prepared figures and text for the manuscript. M. I. A and I. K. H. L designed and performed NMR experiments, analyzed data, and prepared figures and text for the manuscript. C. C. T., O. A, J. P. H., and D. Z. synthesized PHD inhibitors. A. T. performed FIH and KDMs inhibition assays, analyzed data, and prepared figures. K. L. performed PHD1–3 inhibition assays, analyzed data, and prepared figures. C. T. L. performed Tpa1p, Ofd1 and OGFOD1 inhibition assays, analyzed data, and prepared figures. H. M. made the HT1080P8 cell line with the help of C. W. P., T.-L. Y., A. K., E. F., T. D. W. C., X. L., C. W. P., P. J. R., and C. J. S. designed studies. C. J. S. initiated the work, designed experiments, analyzed data, and wrote the manuscript.

## Conflicts of interest

C. J. S., C. W. P., and P. J. R. are co-founders of, and hold equity in, a university spin-out company, ReOx, which aims to exploit basic science discoveries on HIF for therapeutic benefit.

## Supplementary Material

Supplementary information
