# Supplementary material for: Molecular and cellular mechanisms of HIF prolyl hydroxylase inhibitors in clinical trials
Source: Chem Sci. 2017 Sep 11;8(11):7651–68. doi: 10.1039/c7sc02103h (PMC5802278; doi:10.1039/c7sc02103h)
Supplement: Supplementary file 1 [file SC-008-C7SC02103H-s001.pdf]

Electronic Supplementary Material (ESI) for Chemical Science.
This journal is © The Royal Society of Chemistry 2017

Supplementary Information

for

Molecular and Cellular Mechanisms of HIF Prolyl Hydroxylase inhibitors in Clinical Trials

Authors: Tzu-Lan Yeh[1,4], Thomas M. Leissing[1,2], Martine I. Abboud[1], Cyrille C. Thinnes[1], Onur Atasoylu[1], James P. Holt-Martyn[1], Dong Zhang[1], Anthony Tumber[1,3], Kerstin Lippl[1], Christopher T. Lohans[1], Ivanhoe K. H. Leung[1‡], Helen Morcrette[5†], Ian J. Clifton[1], Timothy D. W. Claridge[1], Akane Kawamura[1,5], Emily Flashman[1], Xin Lu[2], Peter J. Ratcliffe[4,6], Rasheduzzaman Chowdhury[1], Christopher W. Pugh[4], and Christopher J. Schofield[1*]

Affiliations:

[1]Chemistry Research Laboratory, Department of Chemistry, University of Oxford, Oxford OX1 3TA, UK.

[2]Ludwig Institute for Cancer Research, Nuffield Department of Clinical Medicine, University of Oxford, Oxford OX3 7DQ, UK.

[3]Structural Genomics Consortium (SGC), University of Oxford, Oxford OX3 7DQ, UK.

[4]Target Discovery Institute (TDI), Nuffield Department of Medicine, University of Oxford, NDMRB Roosevelt Drive, Oxford OX3 7FZ, UK.

[5]Radcliffe Department of Medicine, Division of Cardiovascular Medicine, BHF Centre of Research Excellence, Wellcome Trust Centre for Human Genetics, Roosevelt Drive, Oxford OX3 7BN, UK.

[6]The Francis Crick Institute, 1 Midland Road, London NW1 1AT, UK.

* Corresponding author. Email: christopher.schofield@chem.ox.ac.uk

§ Electronic Supplementary Information (ESI) available: Synthetic procedures; Supplementary methods including NMR, OGFOD1/Tpa1a/OFD1/PHD2/FIH hydroxylation assays, cell culture and immunoblotting, RT-qPCR assays and cell viability assays; Supplementary Figures; Supplementary Tables.

† Current Address: College of Life and Environmental Sciences, University of Exeter, Exeter, Devon, EX4 4QD, UK

‡ Current Address: School of Chemical Sciences, The University of Auckland, Private Bag 92019, Auckland 1142, New Zealand

Materials and Methods

Synthetic Procedures 4
NMR experiments 12
MALDI-TOF MS OGFOD1 hydroxylation assays 13
MALDI-TOF MS OFD1/TPA1p hydroxylation assays 13
LC-MS PHD2 hydroxylation assays 14
LC-MS FIH hydroxylation assays 14
Cell Culture and Immunoblotting 15
RT-qPCR assays 16
Cell viability assays 16
Supplementary Figures 17
Supplementary Tables 38
Reference 44

Supplementary Figures

Fig. S1. PHD catalysed prolyl- and FIH catalyzed asparaginyl-hydroxylation proceed via an ordered sequential process. 17
Fig. S2. Comparison of crystal structure views of FIH complexed with Molidustat and GSK1278863. 18
Fig. S3. Views from crystal structures of FIH.Zn.Vadadustat and $PHD2_{181-426}$.Mn.Vadadustat structure. 19
Fig. S4. Comparison of views from crystal structures from FIH in complex with Molidustat, GSK1278863 and Vadadustat with representative structures of other 2OG-oxygenases with 2OG(NOG)/substrates bound. 20
Fig. S5. Comparison of Binding of CCT6 to PHD2 and GSK1278863 to FIH. 21
Fig. S6. NMR binding assay of FG-4592 to PHD2. 22
Fig. S7. NMR binding assay of GSK1278863 to PHD2. 23
Fig. S8. NMR binding assay of Molidustat to PHD2. 24
Fig. S9. NMR binding assay of IOX4 to PHD2. 25
Fig. S10. Measurement of binding constants between PHD inhibitors and the catalytic domain of PHD2 26
Fig. S11. *In vitro* PHD isoform selectivity profiling. Inhibition of PHD inhibitors against human PHD1-3 as assayed by MALDI-TOF MS 27
Fig. S12. *In vitro* OGFOD1 selectivity profiling. 28
Fig. S13. *In vitro* FIH selectivity profiling. 29
Fig. S14. *In vitro* OFD1 selectivity profiling. 30
Fig. S15. *In vitro* TPA1p selectivity profiling. 31
Fig. S16. Immunoblot showing comparison of HIF-1α and HIF-2α expression in various cell lines. 31
Fig. S17. Comparison of PHD inhibitors on HIF-α stabilization in human cell lines. 32
Fig. S18. Hep3b cell viability after prolonged treatment of PHD inhibitors. 32
Fig. S19. Immunoblots of Hep3b cells treated with 2.5 μM and 10 μM PHD inhibitors for 24 hours. 33

Fig. S20. Effect of Vadadustat on Cellular Inhibition assessed by the HRE luciferase reporter assay. 33
Fig. S21. Effect of Vadadustat on Cellular Activity of the HIF prolyl- and asparaginyl-hydroxylases. 34
Fig. S22. Effect of Vadadustat on HIF-1α stabilization in different cell lines. 35
Fig. S23. Effect of Vadadustat on HIF target gene transcription (with FG-4592 for comparison). 36
Fig. S24. Effect of Vadadustat on HIF target gene transcription (with FG-4592 for comparison). 37

Supplementary Tables

Table S1. Data collection and refinement statistics of FIH and PHD2 complex structures. 38
Table S2. Buffer and vapour diffusion condition used for FIH and PHD2 complex crystallization. 39
Table S3. Selectivity of FG-4592, GSK1278863, Molidustat, IOX4, Vadadustat and Fluoro-Vadadustat against 1μM isolated recombinant human PHD1-3 using the MALDI-TOF MS based assay.[25] 39
Table S4. *In vitro* activity of clinical trial PHD inhibitors against 150 nM isolated recombinant human PHD2 using the LC-MS based RapidFire assay. 40
Table S5. Published phase 2 studies of PHD inhibitors in Anaemia CKD. 41

Materials and Methods

Synthetic Procedures

Molidustat was synthesized as reported[1,2]. Synthetic procedures for GSK1278863, Vadadustat and AKB-6899 were modified from those reported.[3,4] All chemicals used were reagent grade from Sigma Aldrich. Thin layer chromatography was performed using Merck aluminium plates (0.2 mm silica gel-60 F_{254}). After elution, TLC plates were visualized either under UV light or by staining with potassium permanganate or anisaldehyde followed by heating. Chromatographic separations were performed using a Teledyne Isco CombiFlash® Rf+ machine using either the silica gel or reverse phase pre-packed columns (ISCO). Selected infra red absorption frequencies are reported. ^{1}H NMR spectra were recorded in the Fourier Transform mode using a Bruker B-ACS 60 Ultrashield 400 plus spectrometer, operating at a nominal ^{1}H NMR frequency of 400 MHz, using the specified deuterated solvent. All spectra were processed using Topspin 3.0 software. Chemical shifts are reported in ppm and referenced to the residual solvent peak. Multiplicities in the NMR spectra are described as: s = singlet, d = doublet, t = triplet, q = quartet, m = multiplet, br = broad; coupling constants are reported in Hz (to the nearest 0.5 Hz).

Molidustat[1,2]

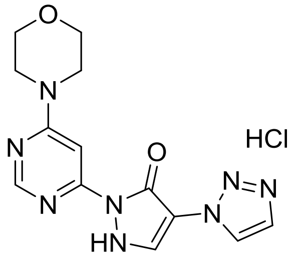

Molidustat was synthesized as reported.[1,2]

mp: 177ºC (dec.);

ν_{max}/cm^{-1} 1689 (CO);

δ_H (400 MHz, DMSO-d_6) δ 8.54 (d, J = 1.0 Hz, 1H), 8.40 (d, J = 1.0 Hz, 1H), 8.28 (s, 1H), 7.90 (d, J = 1.0 Hz, 1H), 7.41 (s, 1H), 3.70 (s, 8H);

δ_C (100 MHz, DMSO-d_6) 162.29, 155.27, 154.24, 152.36, 136.57, 133.37, 124.33, 103.35, 86.24, 66.09, 44.93;

m/z (ESI$^-$) 315.2 ([M-H]$^+$).

GSK1278863 Synthesis[3]

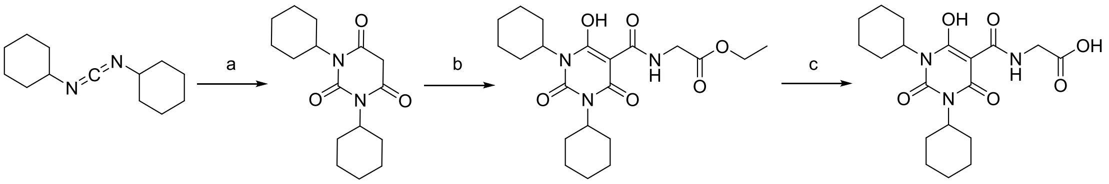

Scheme 1. Synthetic route used for the preparation of GSK1278863. (a) Malonic acid, 0°C, THF, then room temperature, 2h; (b) ethyl isocyanatoacetate, diisopropylethylamine, CH_2Cl_2, 16h; (c) 6M NaOH, H_2O, 3h, then 6M HCl.

1,3-Dicyclohexylpyrimidine-2,4,6(1*H*,3*H*,5*H*) trione[3]

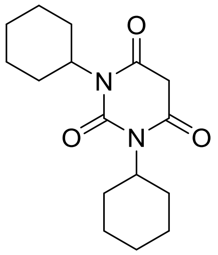

A solution of *N*,*N*'-dicyclohexylcarbodiimide (2.54 g, 12.3 mmol) in THF (10 mL) was slowly added to a solution of malonic acid 641 mg, 6.2 mmol) in THF (10 mL) at 0°C. The resultant reaction mixture was warmed to room temperature, then stirred for 2h. The precipitate was then collected by filtration, then immediately slurried in ethanol (20 mL) and heated to reflux. The mixture was then allowed to cool to room temperature and the precipitate filtered, washed with cold ethanol, and dried to give 1,3-dicyclohexylpyrimidine-2,4,6(1*H*,3*H*,5*H*)-trione (1.3 g, 72 %) as a white powder.

mp 203°C;

ν_{max}/cm^{-1} 1743 (CO), 1691 (CO), 1672 (CO);

δ_H (400 MHz, DMSO-d_6) 4.34 - 4.54 (2 H, m), 3.70 (2 H, s), 2.00 - 2.29 (4 H, m), 1.70 - 1.86 (4 H, m), 1.48 - 1.68 (6 H, m), 1.19 - 1.38 (4 H, m), 1.03 - 1.18 (2 H, m);

δ_C (100 MHz, DMSO-d_6) 166.5, 152.0, 54.2, 41.7, 29.1, 26.4, 25.3;

m/*z* (ESI$^-$) 291 ([M-H]$^-$);

HRMS (ESI$^-$) $C_{16}H_{23}O_3N_2$, ([M-H]$^-$) requires 291.1703; found 291.1716.

Ethyl (1,3-Dicyclohexyl-6-hydroxy-2,4-dioxo-1,2,3,4-tetrahydropyrimidine-5-carbonyl)glycinate[3]

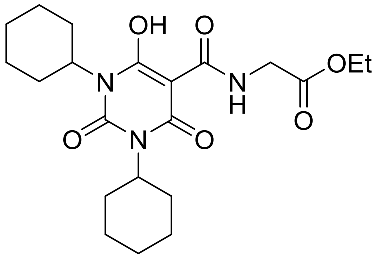

Ethyl isocyanatoacetate (460 µL, 3.7 mmol) was slowly added to a stirred solution of 1,3-dicyclohexylpyrimidine-2,4,6(1*H*,3*H*,5*H*)-trione (1.09 g, 3.7 mmol) and diisopropylethylamine (1.3 mL) in CH_2Cl_2 (15 mL) at room temperature. The resultant solution was stirred at room temperature for 16h. The resultant reaction mixture was extracted with HCl (6M in H_2O, 5 mL); the organic layer was dried over anhydrous Na_2SO_4 and concentrated *in vacuo*. The resulting solid was then slurried in cyclohexane (5 mL) and heated to reflux. The mixture was allowed to cool to room temperature; the precipitate was then collected by filtration and purified *via* flash column chromatography (10 % - 40 % EtOAc, cyclohexane) to give ethyl

(1,3-dicyclohexyl-6-hydroxy-2,4-dioxo-1,2,3,4-tetrahydropyrimidine-5-carbonyl)glycinate (840 mg, 54 %) as a white powder.

mp 163°C;

ν_{max}/cm^{-1} 1750 (CO), 1676 (CO), 1627 (CO);

δ_H (400 MHz, $CDCl_3$) 4.53 - 4.81 (2 H, m), 4.19 (2 H, q, *J*=7.0 Hz, OC*H*$_2$), 4.08 (2 H, d, *J*=5.5 Hz), 2.14 - 2.41 (4 H, m), 1.68 - 1.84 (4 H, m), 1.49 - 1.67 (6 H, m), 1.02 - 1.40 (9 H, m);

δ_C (100 MHz, $CDCl_3$) 171.8, 168.4, 61.9, 41.5, 29.2, 29.0, 26.5, 26.4, 25.3, 25.2, 14.2;

m/*z* (ESI$^-$) 429 ([M-H]$^-$);

HRMS (ESI$^-$) $C_{21}H_{30}O_6N_3$, ([M-H]$^-$) requires 420.2129; found 420.2140.

(1,3-Dicyclohexyl-6-hydroxy-2,4-dioxo-1,2,3,4-tetrahydropyrimidine-5-carbonyl)glycine (GSK1278863)[3]

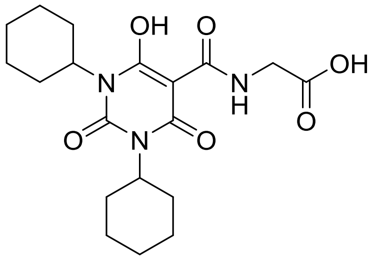

A suspension of ethyl (1,3-dicyclohexyl-6-hydroxy-2,4-dioxo-1,2,3,4-tetrahydropyrimidine-5-carbonyl)glycinate (532 mg, 1.3 mmol) in EtOH (1.5 mL) was treated dropwise with NaOH (6M in H_2O, 250 µL), then stirred for 3 h at room temperature. The reaction mixture was then acidified by dropwise addition of HCl (6M in H_2O, 300 µL) and diluted with H_2O (1 mL). The precipitate was collected by filtration. The crude solid was then slurried in H_2O, heated to 35°C, then stirred vigorously for 1h, filtered, and dried. The solid material was then purified *via* recrystallisation from hot glacial acetic acid (1.5 mL). Subsequent purification *via* flash column chromatography (15 % - 40 % EtOAc, cyclohexane, 0.1 % AcOH) gave purified (1,3-dicyclohexyl-6-hydroxy-2,4-dioxo-1,2,3,4-tetrahydropyrimidine-5-carbonyl)glycine (332 mg, 67 %) as a white solid.

mp 262°C;

ν_{max}/cm^{-1} 1717 (CO), 1662 (CO);

δ_H (400 MHz, DMSO-*d*$_6$) 4.49 - 4.76 (2 H, m), 4.12 (2 H, d, *J*=5.5 Hz), 2.13 - 2.37 (4 H, m), 1.71 - 1.86 (4 H, m), 1.50 - 1.68 (6 H, m), 1.19 - 1.35 (4 H, m), 1.03 - 1.18 (2 H, m);

δ_C (100 MHz, DMSO-*d*$_6$) 171.2, 170.3, 42.0, 29.1, 26.8, 26.5, 25.5;

m/*z* (ESI$^-$) 392 ([M-H]$^-$);

HRMS (ESI$^-$) $C_{19}H_{26}O_6N_3$, ([M-H]$^-$) requires 392.1816; found 392.1833.

Vadadustat Synthesis[4]

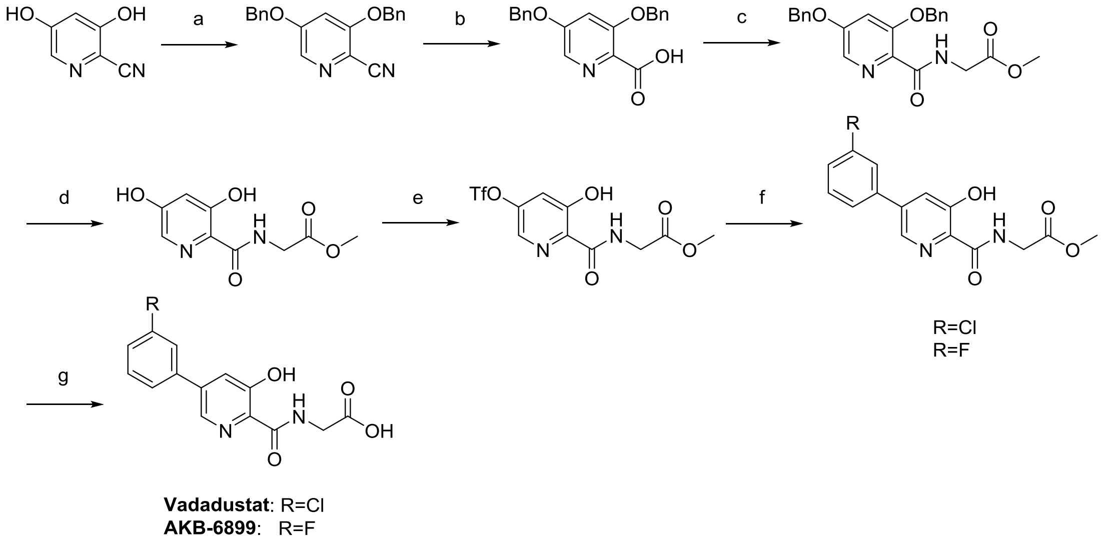

Scheme 2. Route used for preparation of Vadadustat and AKB-6899. (a) Benzyl alcohol, NaH, dry THF, 150°C microwave, 8h; (b) 30% w/v sodium hydroxide, MeOH, reflux, 16h; (c) diisopropyethylamine, propylphosphonic anhydride, dry EtOAc, 0°C, 20 min, then glycine methyl ester hydrochloride, room temperature, 12h; (d) 10% Pd/C, H_2, MeOH, 16h; (e) diisopropylethylamine, N-phenyl trifluoromethansulfonimide, CH_2Cl_2, 12h; (f) Pd(dppf)Cl_2, K_3PO_4, for **1,** 3-chlorophenylboronic acid, for **2,** 3-fluorophenylbornic acid, 1,4-dioxane, 85°C, 16h; (g) 1M LiOH, 2h, then 1M HCl.

3,5-Bisbenzyloxy pyridine-2-carbonitrile[4]

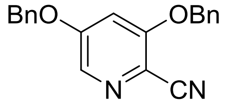

To a 20 mL microwave pressure vessel was added dried THF (10 mL) and benzyl alcohol (661.3 µL, 6.36 mmol). The solution was cooled to 0°C and sodium hydride (266 mg of a 60% dispersion in mineral oil, 6.65 mmol) was added portionwise. The reaction mixture was gradually allowed to warm to room temperature with stirring until the evolution of hydrogen gas ceased. The solution was then re-cooled to 0°C when 3,5-dichloro-2-cyanopyridine (500 mg, 2.89 mmol) was added, and the solution was transferred to an Biotage microwave reactor to 150 °C, 400 W and held for 8 h. The reaction mixture was quenched with H_2O, concentrated *in vacuo*, diluted with EtOAc and washed with 2M Na_2CO_3, H_2O and saturated aqueous NaCl. The organic layer was dried ($MgSO_4$), filtered, and concentrated *in vacuo* to give a brown solid. The crude solid was purified by flash chromatography (EtOAc:cyclohexane, gradient 0:1 to 2:1) to afford 720 mg (78%) of the desired compound as an off white solid.

mp 126.5-127.8 °C;

ν_{max}/cm^{-1}: 2228 (CN);

δ_H (400 MHz, DMSO-d_6): 8.13 (d, J = 2.3 Hz, 1H), 7.56 (d, J = 2.3 Hz, 1H), 7.47 (tt, J = 8.2, 1.9 Hz, 5H), 7.47 – 7.33 (m, 5H), 5.36 (s, 2H), 5.30 (s, 2H);

δ_C (101 MHz, DMSO-d_6): 159.62, 159.12, 135.95, 135.87, 133.12, 129.14, 129.09, 128.90, 128.87, 128.67, 128.30, 116.26, 114.45, 107.87, 71.11, 71.07;

m/z (ESI$^-$) 317.1 ($[M+H]^+$);

HRMS: (ESI$^-$) $C_{20}H_{17}N_2O_2$, ([M+H]$^+$) requires 317.1290; found 317.1284.

3,5-Bisbenzyloxy pyridine-2-carboxylic acid[4]

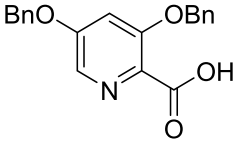

To a solution of 3,5-bisbenzyloxy pyridine-2-carbonitrile, (650 mg, 2.06 mmol) in MeOH (20 mL) was added 30% w/v sodium hydroxide (30 mL); the reaction mixture was then refluxed for 16 h. The solvent was removed *in vacuo* and the resulting suspension was acidified with conc. HCl until the pH was ~3. The aqueous solution was then extracted with EtOAc and washed with H_2O and saturated aqueous NaCl. The organic layer was dried ($MgSO_4$), filtered, and concentrated *in vacuo* to give an orange gel. The crude product was purified by flash chromatography (EtOAc:cyclohexane, gradient 1:4 to 2:1) to afford 600 mg (90% yield) of the desired compound as a brown oil.

ν_{max}/cm^{-1} 2980 (COOH, Br), 1756 (COOH);

δ_H (400 MHz, DMSO-d_6): 12.67 (s, 1H), 7.99 (d, J = 2.5 Hz, 1H), 7.48 (d, J = 1.7 Hz, 1H), 7.47 – 7.32 (m, 10H), 5.25 (d, J = 3.2 Hz, 4H);

δ_C (101 MHz, DMSO-d_6): 165.63, 157.17, 154.76, 136.35, 136.02, 132.71, 128.61, 128.56, 128.45, 128.23, 128.05, 127.87, 127.26, 108.11, 70.14, 69.83;

m/z (ESI$^-$) 336.1 ([M+H]$^+$);

HRMS: (ESI$^-$) $C_{20}H_{18}NO_4$, ([M+H]$^+$) requires 336.1236; found 336.1229.

[(3,5-Bisbenzyloxy pyridine-2-carbonyl) amino]acetic acid methyl ester[4]

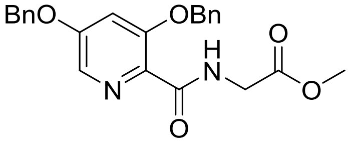

To a solution of 3,5-bisbenzyloxy pyridine-2-carboxylic acid, (500 g, 1.50 mmol) in EtOAc (20 mL) at 0°C (under a N_2 atmosphere) was added diisopropylethylamine (784 µL, 4.5 mmol) and propylphosphonic anhydride (50% by mass) (1.16 ml, 2.00 mmol). The solution was then stirred for 20 minutes, then glycine methyl ester hydrochloride (251 mg, 2.00 mmol) was added. The reaction was allowed to warm slowly to room temperature. The reaction mixture was stirred overnight, then washed with H_2O and saturated aqueous NaCl. The organic layer was dried ($MgSO_4$), filtered and concentrated *in vacuo* to afford a yellow oil that was purified by flash chromatography (EtOAc:cyclohexane gradient 0:1 to 1:2) to afford 503.7 mg (83%) of the desired product as orange crystals.

mp 110.8-112.5 °C;

ν_{max}/cm^{-1} 3400 (N-H), 1748 (COOR), 1671 (CONH);

δ_H (400 MHz, DMSO-d_6): 12.67 (s, 1H), 8.65 (t, J = 6.0 Hz, 1H), 8.01 (d, J = 2.0 Hz, 1H), 7.54 – 7.29 (m, 11H), 5.26 (d, J = 8.5 Hz, 4H), 4.00 (d, J = 6.0 Hz, 2H), 3.64 (s, 3H);

δ_C (101 MHz, DMSO-d_6): 170.43, 164.12, 157.28, 155.15, 136.50, 136.05, 132.78, 128.55, 128.37, 128.28, 128.22, 128.04, 127.71, 127.14, 108.65, 70.14, 69.84, 51.67, 40.77;

m/z (ESI$^-$) 407.2 ($[M+H]^+$);

HRMS: (ESI$^-$) $C_{23}H_{22}N_2O_5$, ($[M+H]^+$) requires 407.1607; found 407.1600.

[(3,5-Dihydroxy pyridine-2-carbonyl) amino]acetic acid methyl ester[4]

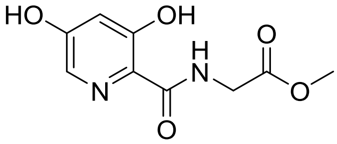

To a solution of [(3,5-bisbenzyloxy pyridine-2-carbonyl) amino]acetic acid methyl ester**,** (500 mg, 1.23 mmol) in MeOH (20 mL) was added 10% Pd/C (14 mg, 0.13 mmol). The reaction mixture was stirred under an atmosphere of H_2 at room temperature for 16 h. The suspension was filtered through a Celite^{TM} pad, the filtrate concentrated *in vacuo*. The crude material was purified by flash chromatography (EtOAc:cyclohexane gradient 0:1 to 1:1) to afford 270 mg (apparent quantitative yield) of the desired compound as an off-white solid.

mp 149.0-150.1 °C;

ν_{max}/cm^{-1} 3363 (O-H, Br), 1738 (COOR), 1640 (CONH);

δ_H (400 MHz, DMSO-*d*$_6$): 12.27 (s, 1H), 10.82 (s, 1H), 9.11 (t, *J* = 6.0 Hz, 1H), 7.76 (d, *J* = 2.5 Hz, 1H), 6.68 (d, *J* = 2.5 Hz, 1H), 4.04 (d, *J* = 6.0 Hz, 2H), 3.66 (s, 3H);

δ_C (101 MHz, DMSO-*d*$_6$): 169.82, 169.10, 158.83, 158.54, 130.25, 123.05, 109.32, 51.89, 40.42;

m/z (ESI$^-$) 227.1 ($[M+H]^+$);

HRMS: (ESI$^-$) $C_9H_{11}N_2O_5$, ($[M+H]^+$) requires 227.0668; found 227.0663.

[(3-Hydroxy-5-trifluoromethane sulfonyloxy pyridine-2-carbonyl) amino]acetic acid methyl ester[4]

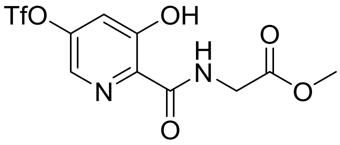

To a solution of [(3,5-dihydroxy pyridine-2-carbonyl) amino]acetic acid methyl ester, (506.3 g, 2.24 mmol) in CH_2Cl_2 (30 mL) was added diisopropylethylamine (DIPEA) (459 mL, 2.65 mmol). The mixture was cooled to 0°C, then N-phenyl triflouoromethansulfonimide (941 m g, 2.65 mmol) was added. The resulting solution was slowly warmed to room temperature and stirred overnight. The solvent was removed *in vacuo*, the crude material was purified by flash chromatography (EtOAc:cyclohexane 1:4) to afford 566.9 mg (71%) of the desired product as an off-white solid.

mp 98.5-99.4 °C;

ν_{max}/cm^{-1} 3337 (O-H, Br), 1743 (C=OOR), 1647 (C=ONH);

δ_H (400 MHz, DMSO-*d*$_6$): 12.63 (s, 1H), 9.61 (t, *J* = 6.0 Hz, 1H), 8.42 (d, *J* = 2.5 Hz, 1H), 7.87 (d, *J* = 2.5 Hz, 1H), 4.10 (d, *J* = 6.0 Hz, 2H), 3.67 (s, 3H);

δ_C (101 MHz, DMSO-*d*$_6$): 169.36, 167.82, 157.98, 148.69, 133.06, 131.31, 119.78, 119.37, 116.59, 52.01, 40.69;

m/z (ESI$^-$) 359.0 ($[M+H]^+$);

HRMS: (ESI$^-$) $C_{10}H_{10}F_3N_2O_7S$, ($[M+H]^+$) requires 359.0161; found 359.0156.

{[5-(3-Chlorophenyl)-3-hydroxypyridine-2-carbonyl] amino}acetic acid methyl ester[4]

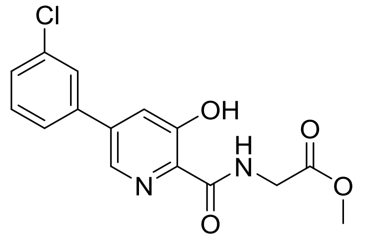

To a degassed solution of [(3-hydroxy-5-trifluoromethane sulfonyloxy pyridine-2-carbonyl) amino] acetic acid methyl ester, (200 mg, 0.56 mmol) in 1,4-dioxane (10 mL) at room temperature under N_2 was added 3-chlorophenylboronic acid (175.7 mg, 1.12 mmol), Pd(dppf)Cl_2 (22 mg, 0.003 mmol) and K_3PO_4 (276 mg, 1.3 mmol). The resulting suspension was heated in a sealed tube at 85°C for 16 h. After this time, the mixture was cooled to room temperature and solvent removed *in vacuo*. The residue was then treated with 1M HCl (1 mL) and diluted with EtOAc. The organic layer was separated, washed with H_2O, saturated aqueous NaCl and concentrated *in vacuo*. The crude material was then purified by flash chromatography (EtOAc:cyclohexane 3:7) to afford 80.8 mg (45%) of the desired compound as a white solid.

mp 128.5-128.8 °C;

ν_{max}/cm^{-1} 3378 (O-H, Br), 1749 (COOR), 1650 (CONH);

δ_H (400 MHz, $CDCl_3$): 11.83 (s, 1H), 8.43 (t, *J* = 5.5 Hz, 1H), 8.29 (d, *J* = 2.0 Hz, 1H), 7.57 (q, *J* = 1.5 Hz, 1H), 7.50 – 7.35 (m, 4H), 4.26 (d, *J* = 5.5 Hz, 2H), 3.82 (s, 3H);

δ_C (101 MHz, $CDCl_3$): 169.60, 168.79, 157.65, 140.73, 138.42, 138.34, 135.17, 130.45, 130.22, 128.97, 127.40, 125.46, 123.82, 52.60, 40.77;

m/z (ESI$^-$) 321.1 ($[M+H]^+$);

HRMS: (ESI$^-$) $C_{15}H_{14}ClN_2O_4$, ($[M+H]^+$) requires 321.0642; found 321.0637.

{[5-(3-Chlorophenyl)-3-hydroxypyridine-2-carabonyl]amino}acetic acid (Vadadustat)[4]

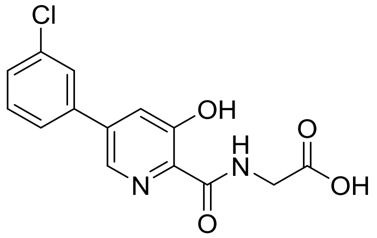

To a solution of {[5-(3-chlorophenyl)-3-hydroxy pyridine-2-carbonyl] amino} acetic acid methyl ester, (50 mg, 0.16 mmol) in THF (2 mL) was added 1M LiOH (1.5 mL). The reaction mixture was stirred at room temperature for 2 h. The solution was acidified using 1M HCl (2 mL); the solvent was then removed *in vacuo* and the resulting solid suspended in $CHCl_3$:iso-propanol (1:1), and filtered. The resultant filtrate was dried ($MgSO_4$), filtered and re-concentrated *in vacuo*. The crude material was triturated with a small amount of MeOH to afford 34.2 mg (70%) of the desired product as a colourless solid.

mp 146.3-147.9 °C;

ν_{max}/cm^{-1} 3366 (O-H, Br), Br 3067 (COOH), 1730 (COOH), 1649 (CONH);

δ_H (400 MHz, DMSO-d_6): 12.38 (s, 1H), 9.38 (t, J = 6.0 Hz, 1H), 8.55 (d, J = 2.0 Hz, 1H), 7.93 (d, J = 2.0 Hz, 1H), 7.86 – 7.71 (m, 2H), 7.54 (dd, J = 5.0, 1.5 Hz, 2H), 4.01 (d, J = 6.0 Hz, 2H);

δ_C (101 MHz, DMSO-d_6): 170.94, 169.19, 157.61, 139.93, 138.84, 138.33, 134.44, 131.42, 130.68, 129.34, 127.56, 126.56, 123.92, 41.16;

m/z (ESI$^-$) 307.0 ([M+H]$^+$);

HRMS: (ESI$^-$) $C_{14}H_{11}ClN_2O_4$, ([M+H]$^+$) requires 307.0486; found 307.0480.

{[5-(3-Fluorophenyl)-3-hydroxy pyridine-2-carbonyl] amino}acetic acid methyl ester[4]

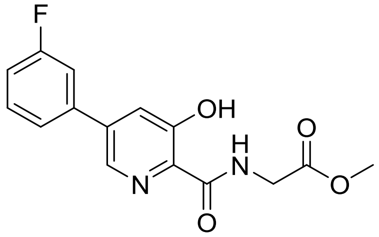

To a degassed solution of [(3-hydroxy-5-triflouoromethane sulfonyloxy pyridine-2-carbonyl) amino] acetic acid methyl ester, (200 mg, 0.56 mmol) in 1,4-dioxane (10 mL) at room temperature under N_2 was added 3-fluorophenylboronic acid (156.7 mg, 1.12 mmol), Pd(dppf)Cl_2 (22 mg, 0.003 mmol) and K_3PO_4 (276 mg, 1.3 mmol). The resultant suspension was heated in a sealed tube at 85°C for 16 h. After this time, the mixture was cooled to room temperature and solvent removed *in vacuo*. The residue was then treated with 1M HCl (1 mL) and diluted with EtOAc. The organic layer was separated, washed with H_2O, saturated aqueous NaCl and concentrated *in vacuo*. The crude material was then purified by flash chromatography (EtOAc:cyclohexane 3:7), to give the desired product (contaminated with some starting material), which was used for next step without further purification.

mp 114.1-117.5 °C;

ν_{max}/cm^{-1} 3288 (O-H, Br), 1790 (COOR), 1659 (CONH);

δ_H (400 MHz, DMSO-d_6): 12.28 (s, 1H), 9.52 (t, J = 6.0 Hz, 1H), 8.56 (d, J = 2.0 Hz, 1H), 7.79 (d, J = 2.0 Hz, 1H), 7.76 – 7.66 (m, 2H), 7.59 – 7.53 (m, 1H), 7.35 – 7.28 (m, 1H), 4.11 (d, J = 6.0 Hz, 2H), 3.68 (s, 3H);

δ_C (101 MHz, DMSO-d_6): 169.63, 168.91, 162.67 (d, J = 244.0 Hz), 157.19, 139.68, 138.46, 138.07 (d, J = 8.0 Hz), 131.16 (d, J = 8.5 Hz), 130.10, 123.50 (d, J = 3.0 Hz), 123.45, 115.85 (d, J = 21.0 Hz), 114.24 (d, J = 22.5 Hz), 52.00, 40.64;

m/z (ESI$^-$) 305.1 ([M+H]$^+$);

HRMS: (ESI$^-$) $C_{14}H_{11}FN_2O_4$, ([M+H]$^+$) requires 305.0938; found 305.0932.

{[5-(3-Fluorophenyl)-3-hydroxypyridine-2-carabonyl] amino}acetic acid (AKB-6899)[4]

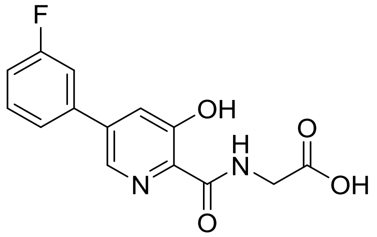

To a solution of {[5-(3-fluorophenyl)-3-hydroxy pyridine-2-carbonyl] amino} acetic acid methyl ester, (50 mg, 0.16 mmol) in THF (2 mL) was added 1M LiOH (1.5 mL). The reaction mixture was stirred at room temperature for 2 h. The solution was acidified using 1M HCl (2 mL); the solvent was then removed *in vacuo* and the resulting solid suspended in $CHCl_3$:iso-propanol (1:1), and filtered. The resultant filtrate was dried ($MgSO_4$), filtered and re-concentrated *in vacuo*. The crude material was triturated with a small amount of MeOH to afford 34.8 mg (73%) of the desired product as a white solid.

mp 92.4-96.1 °C;

ν_{max}/cm^{-1} Br 3296 (O-H), Br 3009 (COOH), 1680 (COOH), 1600 (CONH);

δ_H (400 MHz, DMSO-d_6): 12.38 (s, 1H), 9.33 (t, J = 6.0 Hz, 1H), 8.56 (d, J = 2.0 Hz, 1H), 7.78 (d, J = 2.0 Hz, 1H), 7.73 (ddd, J = 10.5, 2.5, 1.5 Hz, 1H), 7.69 (ddd, J = 8.0, 2.0, 1.0 Hz, 1H), 7.56 (td, J = 8.0, 6.0 Hz, 1H), 7.35 – 7.27 (m, 1H), 4.00 (d, J = 6.0 Hz, 2H);

δ_C (101 MHz, DMSO-d_6): 170.91, 169.11, 163.12 (d, J = 244.0 Hz), 157.62, 140.00, 138.81, 138.56 (d, J = 8.0 Hz), 131.62 (d, J = 9.0 Hz), 130.70, 123.92 (d, J = 3.0 Hz), 123.83, 116.28 (d, J = 21.0 Hz), 114.66 (d, J = 23.0 Hz), 41.31;

m/z (ESI^-) 291.1 ($[M+H]^+$);

HRMS: (ESI^-) $C_{14}H_{11}FN_2O_4$, ($[M+H]^+$) requires 291.0781; found 291.0775.

NMR experiments

Nuclear Magnetic Resonance (NMR) spectra were recorded using either a Bruker AVIII 600 MHz NMR spectrometer equipped with a BB-$^{19}F/^{1}H$ Prodigy N_2 cryoprobe, or a Bruker AVIII 700 MHz NMR spectrometer equipped with a 5-mm inverse cryoprobe using 5 mm diameter NMR tubes (Norell) or 3 mm MATCH NMR tubes (Cortectnet). Data were processed using the Bruker 3.1 software. Prior to Fourier transformation, data were processed with a line broadening of 0.3 Hz. The catalytic domain of human PHD2 (residues 181-426) was used in all the protein NMR experiments.

For ^{1}H excitation sculpting suppression NMR experiments, the assay mixture contained 50 µM *apo*-PHD2 (obtained by EDTA treatment) supplemented with 200 µM Zn(II) and 500 µM of the compound to be studied buffered with 50 mM Tris-D_{11} (pH 7.5) and 0.02 % NaN_3 in 90 % H_2O and 10 % D_2O. Spectra were typically obtained using 256 scans and a relaxation delay of 1 s. A 2 ms Sinc pulse was used for water suppression.

For ^{1}H Carr-Purcell-Meiboom-Gill (CPMG) NMR experiments, the assay mixtures contained 50 µM *apo*-PHD2 supplemented with 200 µM Zn(II) and 500 µM of the compound to be studied buffered with 50 mM Tris-D_{11} (pH 7.5) and 0.02 % NaN_3 in 90 % H_2O and 10 % D_2O. Typical experimental parameters for CPMG NMR spectroscopy were as follows: total echo time, 40 ms; relaxation delay, 2 s; number of transients, 1024. The PROJECT-CPMG sequence ($90°_x–[\tau–180°_y–\tau–90°_y–\tau–180°_y–\tau]_n$–acq) as described by Augilar *et al.* was used.[5] Water suppression was achieved by pre-saturation.

Water-Ligand Observed Gradient SpectroscopY (wLOGSY) experiments were conducted using the pulse sequence of Dalvit *et al.*[6] Unless stated, all experiments were at 277 K. Typical parameters were as follows: mixing time, 1 s; relaxation delay, 2 s; number of transients, 256. Solvent excitation was achieved using a 16 ms 180 degree selective rectangular shape pulse with 100% truncation level and 1000 points (Squa100.1000) set at the H_2O frequency. Water suppression was achieved by a 2 ms Sinc (Sinc1.1000) pulse at the H_2O frequency.

2OG displacement experiments were monitored by CPMG-edited ^{1}H NMR using a Bruker AV600 instrument. Samples contained 10 µM 2OG, 10 µM *apo*-PHD2$_{181\text{-}426}$ (if necessary) supplemented with 60 µM Zn(II) and increasing concentrations of the inhibitors from a DMSO-D_6 stock buffered with 50 mM Tris-D_{11} (pH 7.5) and 0.02 % NaN_3 in 85 % H_2O and 10 % D_2O at 298 K. The PROJECT-CPMG sequence: $(90°_x–[\tau–180°_y–\tau–90°_y–\tau–180°_y–\tau]_n–acq)$, was applied. Typical experimental parameters were as follows: total echo time, 40 ms; relaxation delay, 2 s; number of transients, 264. Water suppression was achieved by presaturation.

For ^{13}C-CODD and ^{13}N-CODD displacement experiments, 1D Clean In-Phase (CLIP) HSQC with selective ^{13}C-inversion displacement experiments were conducted using a Bruker AV700 instrument. 3 mm MATCH NMR tubes were used (Cortecnet). All experiments were conducted at 298 K. The reporter CODD or NODD molecule was uniformly ^{13}C labelled on its prolyl-ring. [7] The CLIP-HSQC sequence was used for 1D HSQC experiments (without ^{13}C decoupling).[8] Relaxation delay was 2s. The $^1J_{CH}$ was set to 160 Hz. A 6.8 ms Q3.1000 180 degree pulse was used and selective irradiation was applied at the selected chemical shift.

K_D measurements were carried out as reported by Leung *et al.* using the PHD2.Zn(II) complex and 2OG as a reporter ligand.[9] Water relaxation experiments were conducted using a Bruker AV500 instrument using the PHD2.Mn(II) complex as reported by Leung *et al.*[10]

MALDI-TOF MS OGFOD1 hydroxylation assays

Recombinant human OGFOD1 was prepared as reported,[11] and stored at -80 °C in 50 mM HEPES (pH 7.5), 150 mM NaCl, 1 mM DTT and 5% (*w*/*v*) glycerol after purification with immobilized metal affinity chromatography. Activity assays were performed by determining the extent of hydroxylation of a 20-residue fragment of human RPS23 containing amino acid residues 51-70 (H_2N-VLEKVGVEAKQPNSAIRKCV-$CONH_2$) by matrix-assisted laser desorption/ionisation time-of-flight mass spectrometry (MALDI-TOF MS) using a Waters® Micromass® MALDI micro MX™ mass spectrometer and MassLynx™ 4.1 as described [12]. The optimised hydroxylation assay involved incubation of OGFOD1 (1 µM) with inhibitor (1 % *v*/*v* in DMSO) in the presence of Fe(II) (50 µM), 2OG (25 µM), L-ascorbic acid (100 µM) and RPS23$_{51\text{-}70}$ (25 µM) in HEPES (50 mM, pH 7.5) at 37 °C for 15 min. Reagent solution were made as previously reported. *(64)* Reactions were quenched with formic acid (1 % *v*/*v*). Samples were prepared by mixing reaction mixture (1 µL) with α-cyano-4-hydrocinnamic acid (CHCA) solution (water:acetonitrile 1:1) (1 µL). Dose-response was assessed in 8-point triplicates. Data were analysed using GraphPad Prism 5.04.

MALDI-TOF MS OFD1/TPA1p hydroxylation assays[13]

For enzyme production, the reported procedures were used.[13] *E. coli* BL21(DE3) cells transformed with pNIC28-His6-Ofd1 or pNIC28-His6-Tpa1pΔ2-20 were cultured to an OD600 of 0.6; recombinant protein production was induced through the addition of isopropyl β-D-1-thiogalactopyranoside (IPTG) to a concentration of 0.5 mM. The induced cultures were

incubated overnight at 18°C with shaking, then harvested. The induced cells were suspended in 500 mM NaCl, 40 mM imidazole, 50 mM HEPES pH 7.5, sonicated, and the His-tagged protein was purified by nickel affinity chromatography.[13] Both Ofd1 and Tpa1p were buffer-exchanged into 500 mM NaCl, 5% glycerol, 1 mM DTT, 50 mM HEPES pH 7.5 following immobilized metal affinity chromatography, flash-frozen and stored at -80°C. Enzyme assays consisted of Ofd1 (1 µM) or Tpa1p (2 µM) with 2OG (20 µM), L-ascorbic acid (100 µM), $(NH_4)_2Fe(SO_4)_2$ (50 µM), RPS23 peptide (amino acid sequence AKGIVLEKVGVEAKQPNSAIRKCVRVQLIKNGKKITAF-$CONH_2$; 25 µM) and the relevant inhibitor (initially prepared in 10% DMSO, diluted to 1% DMSO in the reaction mixture) all in 50 mM HEPES pH 7.5. Reactions were quenched after 2 min (Ofd1) or 4 min (Tpa1p) by the addition of formic acid to a final concentration of 1%. The extent of hydroxylation was monitored by MALDI-TOF MS, and the IC_{50} curve was fit to the data using Prism.

LC-MS PHD2 hydroxylation assays

The PHD2 RF-MS RapidFire liquid chromatography mass spectrometry (RF-MS) assay monitors turnover of a C-terminal oxygenase dependent domain (CODD) peptide substrate DLDLEMLAPYIPMDDDFQL-$CONH_2$ and appearance of the hydroxylated peptide product (Pro564) in an endpoint type assay format (typical enzyme incubation time of 15 minutes). Tris(hydroxymethyl)aminomethane was from Fisher. Ferrous ammonium sulfate (FAS), 2-oxoglutarate (2OG) and L-ascorbic acid were from Sigma Aldrich; solutions of these were prepared freshly each day. All inhibition assays were carried out in 384-well polypropylene plates (Greiner Bio-One). PHD2 assays were performed in assay buffer (50 mM Tris.Cl pH 7.5, 50 mM NaCl). Titrations of compounds for IC_{50} determinations (3-fold and 11-point IC_{50}) were prepared using an ECHO 550 acoustic dispenser (Labcyte) and dry dispensed into 384-well polypropylene assay plates. The final assay concentration of DMSO was kept constant at 0.5%. PHD2 protein was prepared at a concentration of 300 nM in assay buffer and 25 µl dispensed across each 384-well assay plate. The PHD2 solution was allowed to equilibrate with the inhibitors for 15 minutes at room temperature; the enzyme reaction then initiated by dispense of 25 µl of substrate (20 µM FAS, 200 µM L-ascorbic acid, 10 µM CODD peptide and 20 µM 2OG in the assay buffer. Enzyme reactions were allowed to proceed for 20 minutes at room temperature and the reaction terminated by addition of 10% formic acid (5 µl). Assay plates were then transferred to a RapidFire RF360 sampling robot (Agilent) connected to an Agilent 6530 accurate mass quadrupole-time-of-flight (Q-TOF) mass spectrometer. Assay samples were aspirated under vacuum and loaded onto a C4 solid phase extraction (SPE) cartridge. After loading the C4 SPE was washed with 0.1 % formic acid in water to remove non-volatile buffer salts and then peptide was eluted from the SPE with 85% acetonitrile, 15% water containing 0.1% formic acid onto the mass spectrometer. Peptide charge states were monitored in positive mode. Ion chromatogram data were extracted for the +2 charge state and peak area data integrated using RapidFire Integrator software (Agilent). % conversion of the CODD peptide substrate to the +16 hydroxylated peptide was calculated using the equation: % conversion = 100 x hydroxylated / (hydroxylated + non-hydroxylated peptide). IC_{50} values were determined from non-linear regression plots using GraphPad prism.

LC-MS FIH hydroxylation assays

Tris(hydroxymethyl)aminomethane was from Fisher; all other reagents were from Sigma Aldrich and of the highest available purity. Ferrous ammonium sulphate (FAS) was prepared

freshly every day as a 400 mM stock solution in 20 mM HCl, this was then diluted to 1 mM in deionized water. L-ascorbic acid (50 mM) and 2OG stock solutions (10 mM) were prepared freshly every day in deionized water as for the PHD2 assays. The synthetic consensus ankyrin peptide (HLEVVKLLLEAGADVNAQDK-$CONH_2$)[14] was synthesized by GL Biochem (Shanghai) Ltd and dissolved to 1 mM in deionized water.

20 µl FIH (100 nM) in the assay buffer (50 mM Tris.Cl pH 7.8, 50 mM NaCl) was pre-incubated for 15 minutes in the presence of the inhibitors and the enzyme reaction initiated by addition of 20 µl substrate (200 µM L-ascorbic acid, 20 µM Fe^{2+}, 10 µM synthetic ankyrin peptide [14] and 20 µM 2OG). After 15 minutes the reaction was quenched by addition of 4 µl 10% formic acid and the reaction transferred to a RapidFire RF360 high throughput sampling robot. Samples were aspirated under vacuum onto a C4 Solid Phase Extraction (SPE) cartridge. After an aqueous washing step (0.1% formic acid in water), to remove non-volatile buffer components from the C4 SPE, peptide was eluted in an organic washing step (85% acetonitrile in water, 0.1% formic acid) and injected into an Agilent 6530 Q-TOF mass spectrometer. Ion chromatogram data was extracted for the non-hydroxylated peptide substrate and the hydroxylated peptide product; the peak area data for extracted ion chromatograms were integrated using RapidFire Integrator software. % conversion of substrate to product was calculated in Microsoft excel and IC_{50} curves generated using Graphpad Prism.

Cell Culture and Immunoblotting

HeLa and RCC4 cells were cultured in Dulbecco’s Modified Eagle’s Medium (DMEM; Sigma Aldrich D6546-500ML) supplemented with 10% fetal bovine serum (Sigma Aldrich F7524-500ML), 4 mM L-glutamine (Sigma Aldrich G7513- 100ML), 50 U/mL penicillin, and 50 µg/mL streptomycin (Sigma Aldrich P0781-100ML). Cells were seeded to reach 90-100% confluency prior to compound treatment. Cells were exposed to the inhibitor at a final concentration of 1% DMSO for 5-6 hours. For Fe(II) titration in the presence of inhibitors, ferric ammonium citrate was added to the cells for further 16 hours after 6 hours of incubation with the inhibitors.

Cells were harvested in urea/SDS buffer (6.7 M urea, 10 mM Tris-Cl pH 6.8, 10% glycerol, and1% SDS) supplemented with 1mM dithiothreitol following a phosphate-buffered saline (PBS) rinse. Cell extracts were analyzed with SDS-PAGE, electroblotted to polyvinylidene difluoride (PVDF) membrane (Millipore) and probed with the corresponding primary antibody for 1 hour at room temperature or overnight at 4 °C. The secondary antibody specific to primary antibody species conjugated with HRP were applied at room temperature for 1 hour. The antibodies used for immonoblotting were as follows: pan-anti-HIF-1α (clone 54, BD Transduction Laboratories™), anti-HIF-1α Hyp402 (catalogue number 07-1585, Merck Millipore), anti-HIF-1α Hyp564 (D43B5, Cell Signaling), anti-HIF-1α HyN803 (a generous gift from Lee et al.[15]), PHD2[16], PHD3[16], and HRP-conjugated anti-β-actin (clone AC15, Abcam). Details of antibodies used are given in Tian et al.[17] Detection of signal was achieved using SuperSignal™ West Dura Extended Duration Substrate kit (Thermo Scientific 34075, unless otherwise stated) either with film or ChemiDoc™ MP (BIO-RAD) imaging system. Quantification of the immunobloting signal was performed with Image Lab™ software from BIO-RAD.

RT-qPCR assays

Cells were lysed using the TRI reagent® (Sigma T9424). RNA was then extracted by phase separation following the manufacturer's protocol. Extracted RNA was treated with the RNase-free DNase I (DNA-*free*™ DNA Removal Kit, Applied Biosystems AM1906) to eliminate contaminating DNA. For reverse transcription, 1μg of DNase-treated RNA samples were used for cDNA synthesis using High-Capacity cDNA Reverse Transcription Kit (Thermo Fisher Scientific 4368814). RT-qPCR assays were performed with SYBR Green dye (Applied Biosystems 4385612) on a StepOnePlus thermocycler (Applied Biosystems). Quantitation of relative expression level was analyzed using the $\Delta\Delta C_T$ method[18] with *HPRT* as a reference gene using; statistical analyses of the biological and technical repeats were calculated using StepOne Software (version 2.2.2). Sequences of primers used were: *HPRT*- forward 5′ GACCAGTCAACAGGGGACAT 3′, reverse 5′ AACACTTCGTGGGGTCCTTTTC 3′, *BNIP3* – forward 5′ CGTTCCAGCCTCGGTTTCTATT 3′, reverse 5′ GAGCGAGGTGGGCTGTCAC 3′, *CA9* - forward 5′ AGCACAGAAGGGGAACCAAAG 3′, reverse 5′ ATGAGCAGGACAGGACAGTTAC 3′, *EPO* - forward 5′ GAGCCCAGAAGGAAGCCATC 3′, reverse 5′ CGGAAAGTGTCAGCAGTGATTG 3′, *PHD3* - forward 5′ CTGGTCCTCTACTGCGGGA 3′, reverse 5′ AGCCACCATTGCCTTAGACCTC 3′, *SLC2A1* - forward 5′ GCCAAGAGTGTGCTAAAGAAGC 3′, reverse 5′ GCCGACTCTCTTCCTTCATCTC 3′, and *VEGFA* – forward 5′ GGCAGAATCATCACGAAGTGGT 3′, reverse 5′ GGCACACAGGATGGCTTGAA 3′.

Cell viability assays

Cell seeding and inhibitor treatments were performed in parallel in the same manner as used for the HRE reporter assay (see above). Resazurin (Sigma, R7017) was used for detection of cell viability at a final concentration of 10μg/ml. Cells were incubated with Resazurin at 37°C for 1-2 hours. Fluorescence signals were measured with an excitation wavelength of 544 nm and emission wavelength of 590 nm using a FLUOstar Omega Microplate Reader (BMG Labtech).

Supplementary Figures

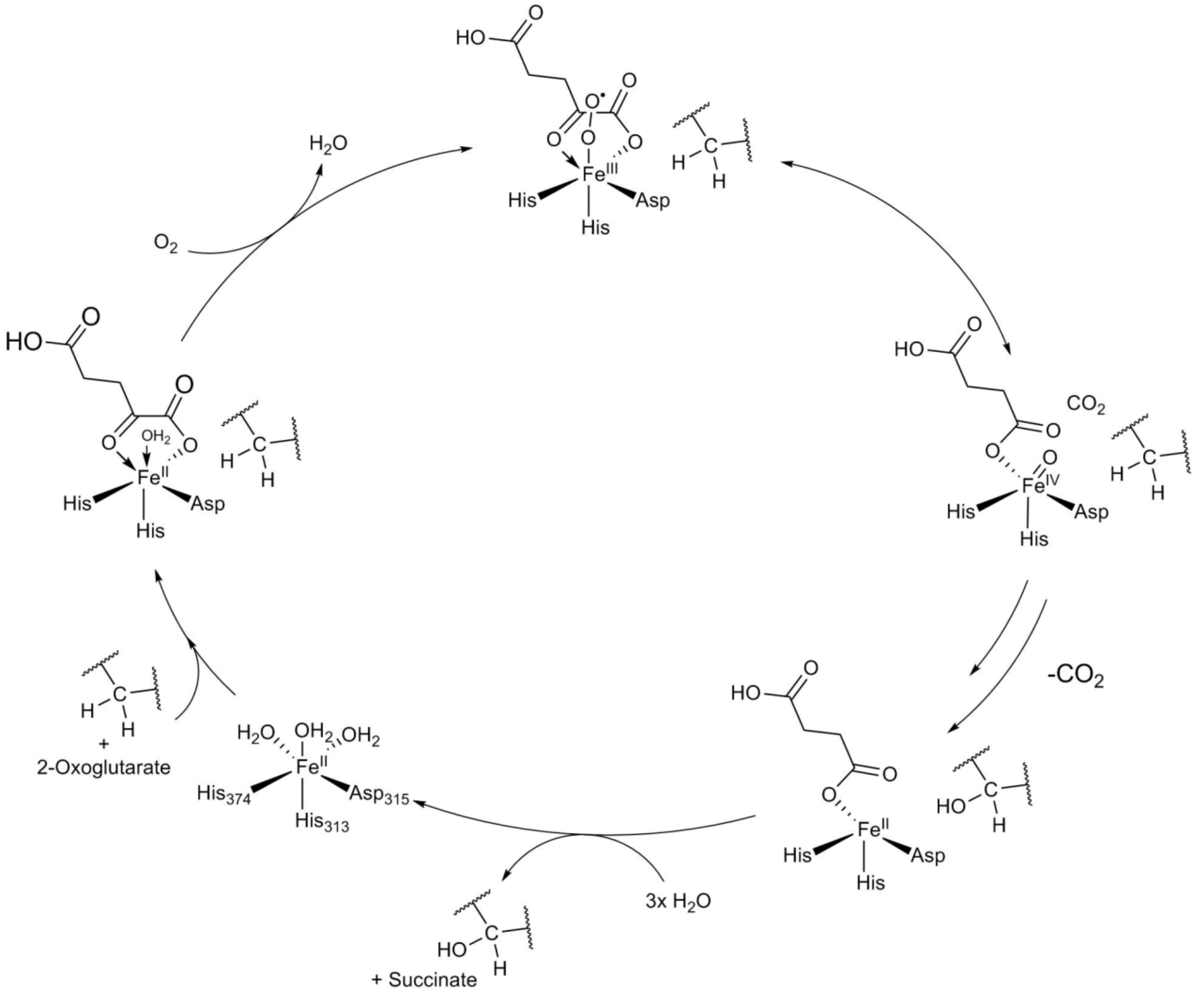

Fig. S1. PHD catalysed prolyl- and FIH catalyzed asparaginyl-hydroxylation proceed via an ordered sequential process. Residue numbers correspond to those for PHD2.

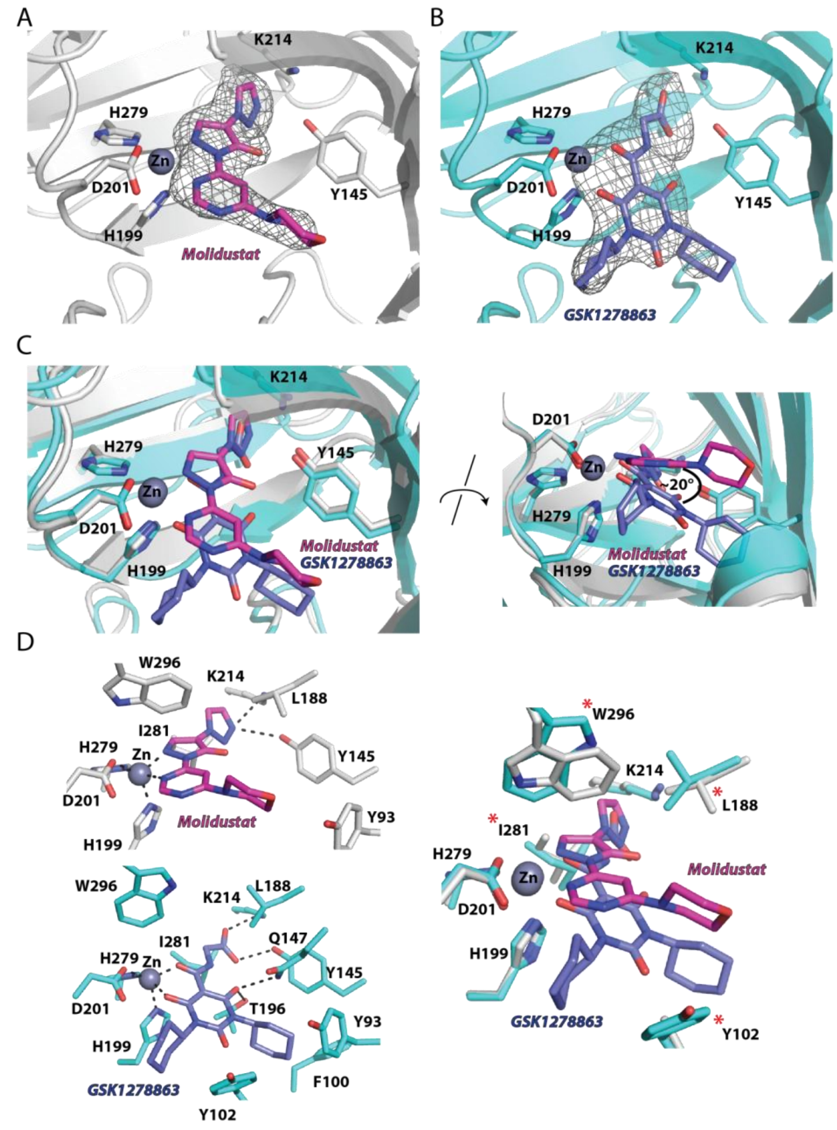

Fig. S2. Comparison of crystal structure views of FIH complexed with Molidustat and GSK1278863. Views FIH structures in complex with (**A**) Molidustat and (**B**) GSK1278863; Fo-Fc OMIT maps are contoured to 3σ. (**C**) Overlay of FIH.Zn.Molidustat and FIH.Zn.GSK1278863 structures indicates reasonable conservation of overall binding modes for the 2 inhibitors, including with regard to the bidentate metal coordination, interactions with the 2OG methylene binding pocket, and the 'binding direction' of the Molidustat morpholine ring and one GSK1278863 cyclohexyl ring. The "metal binding planes", as defined by binding of the Molidustat pyrazolone and pyrimidine rings and the GSK1278863 pyrimidine-trione ring to the metal, show an 'angular difference' of ~20°. (**D**) Interactions of Molidustat/GSK1278863 in the FIH active site. In addition to electrostatic interactions with the metal and 2OG C-5 carboxylate binding pocket, Molidustat is positioned to form hydrophobic interactions between its triazole ring and Ile-281/Leu-188; and with Trp-296 and its metal coordinating rings. The observed GSK1278863 binding mode implies it is not positioned to form hydrophobic interactions with Ile-281/Leu-188, but to make electrostatic interactions with Lys-147/Thr-196; one of the GSK1278863 cyclohexyl rings projects into a hydrophobic region (Tyr-93/Phe-100). This binding mode apparently pushes the flexible residue Tyr-102 to new conformation relative to that in FIH structures with NOFD (PDB:1YCI)[19] or Daminozide (PDB:4AI8)[20]; (the Tyr-102 side chain is disordered in the FIH-Molidustat complex GSK1278863).

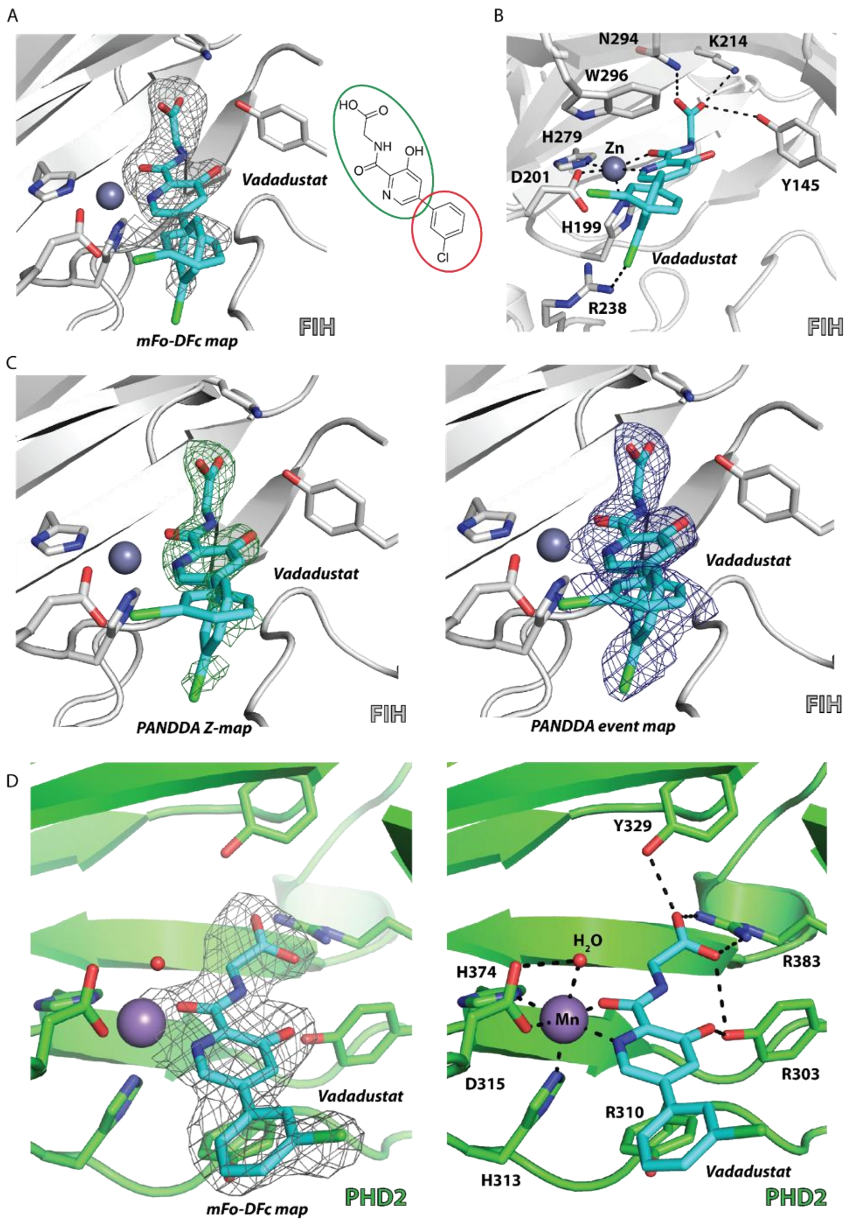

Fig. S3. Views from crystal structures of FIH.Zn.Vadadustat and $PHD2_{181\text{-}426}$.Mn.Vadadustat structure. (A) Views from a crystal structure of FIH in complex with Vadadustat showing the Fo-Fc OMIT map contoured to 3σ. Note that the fit of the electron density map for the N-glycinamide and hydroxy-pyridine groups is good, whereas that for the chlorophenyl group is poorer, likely due to conformational flexibility of the chlorophenyl group. (B) Two major conformations were modelled, likely stabilized by weak hydrogen bonds with Arg-238 or interactions with Trp-296 in each case. Models for the side-chain orientation were based on 'pan-dataset' density analysis.[21] (C) Views from the FIH-Vadadustat crystal structure left (the Z-map contoured to 6σ) and, right, the PANDDA event map (2.60 Å resolution) with a 'Background Density Correction factor' (BDC) of 0.33 (contoured to 3σ), the latter implies two preferred confirmations for the chlorophenyl group. (D) Views from a crystal structure of $PHD2_{181\text{-}426}$.Mn.Vadadustat the Fo-Fc OMIT map contoured to 3σ.

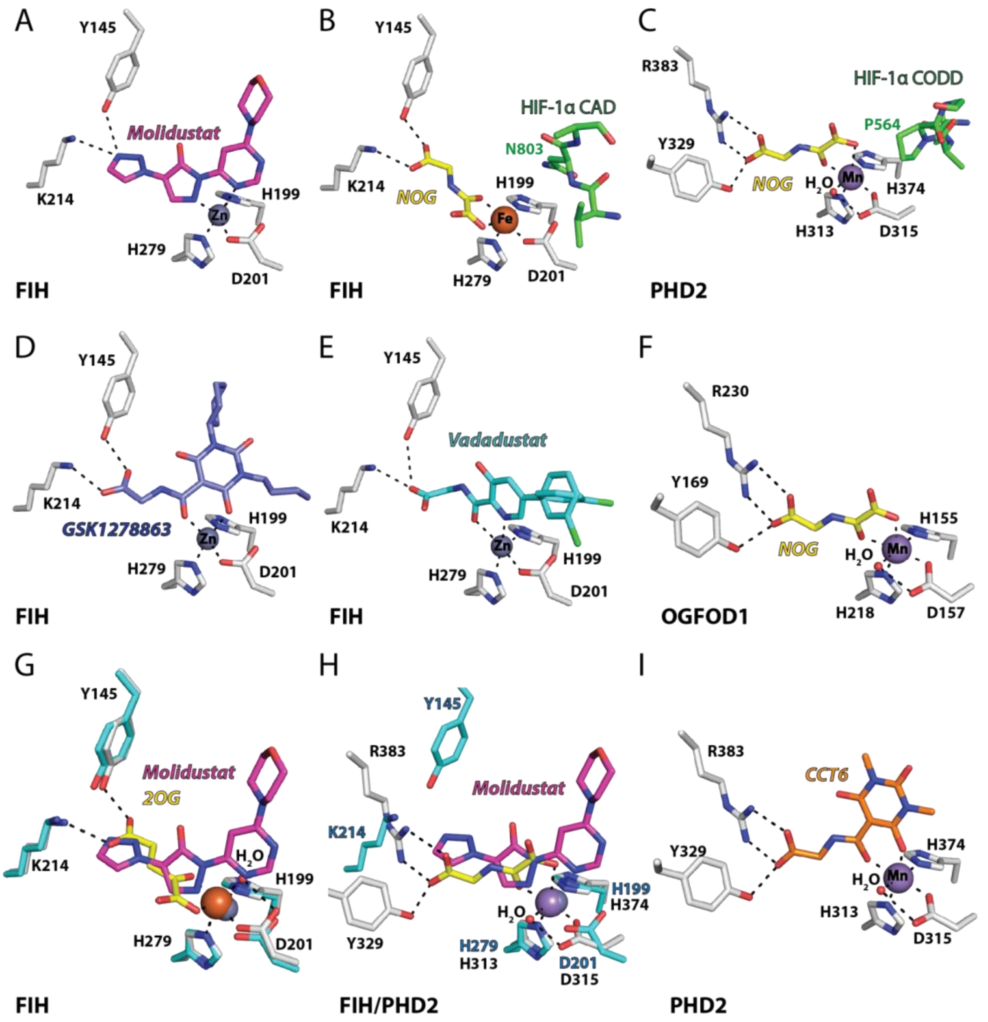

Fig. S4. Comparison of views from crystal structures from FIH in complex with Molidustat, GSK1278863 and Vadadustat with representative structures of other 2OG-oxygenases with 2OG(NOG)/substrates bound. Views from crystal structures of FIH complexed with Molidustat, GSK1278863, NOG and HIF-1α CAD (PDB code 1H2K)[22], and Vadadustat; PHD in complex with CCT6; NOG and HIF-1α CODD (PDB code 3HQR)[23]; and OGFOD1 in complex with NOG (PDB code 4NHX)[11]. Analysis of the binding modes of Molidustat, GSK1278863, and Vadadustat reveals both inhibitors coordinate the active site metal in a bidentate manner. All three inhibitors coordinate the metal *trans* to Asp-201 and His-279; this contrasts with the structure with 2OG which coordinates the metal trans to Asp-201 and His-199. The coordination site *trans* to the distal histidine (His-279) is occupied by a water molecule in some FIH substrate complex structures. The metal coordination site trans to His-199 in the FIH Molidustat, GSK1278863 and Vadadustat structures is not occupied by a detectable ligand. In prolyl hydroxylases and structurally related 2OG oxygenases, however, 2OG coordinates the metal trans to the distal histidine and acidic metal binding residue (His-313 and Asp-315)[24]. Overlay of the FIH.Zn.Molidustat complex structure with the FIH.Fe.2OG (PDB 2Y0I) and PHD2.Mn.NOG (PDB 3HQR) complex structures reveals that the binding mode of Molidustat more closely reassembles the active site PHD2-2OG binding rather than FIH-2OG binding.

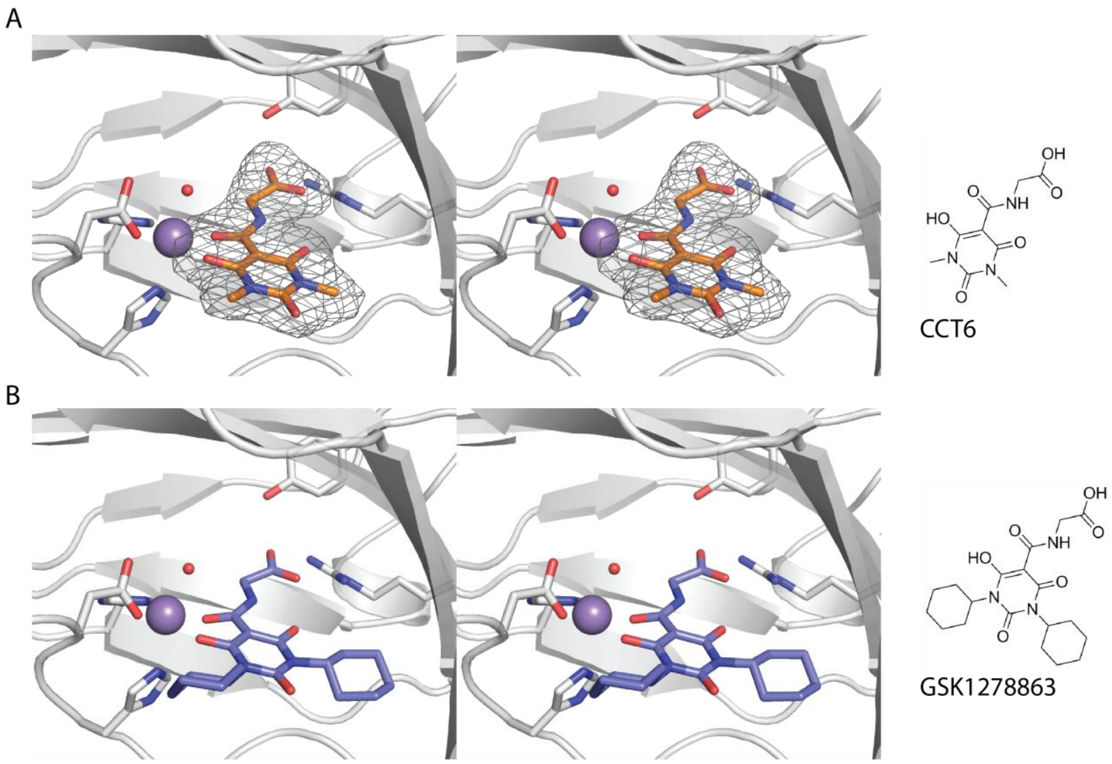

Fig. S5. Comparison of Binding of CCT6 to PHD2 and GSK1278863 to FIH. (**A**) Stereoview from a structure of PHD2 complexed with Mn(II) and CCT6 showing the Fo-Fc OMIT electron density map contoured to 3σ. (**B**) Stereoview from a model for GSK1278863 binding to the active site of PHD2 based on the interactions of CCT6 with the PHD2 active site.

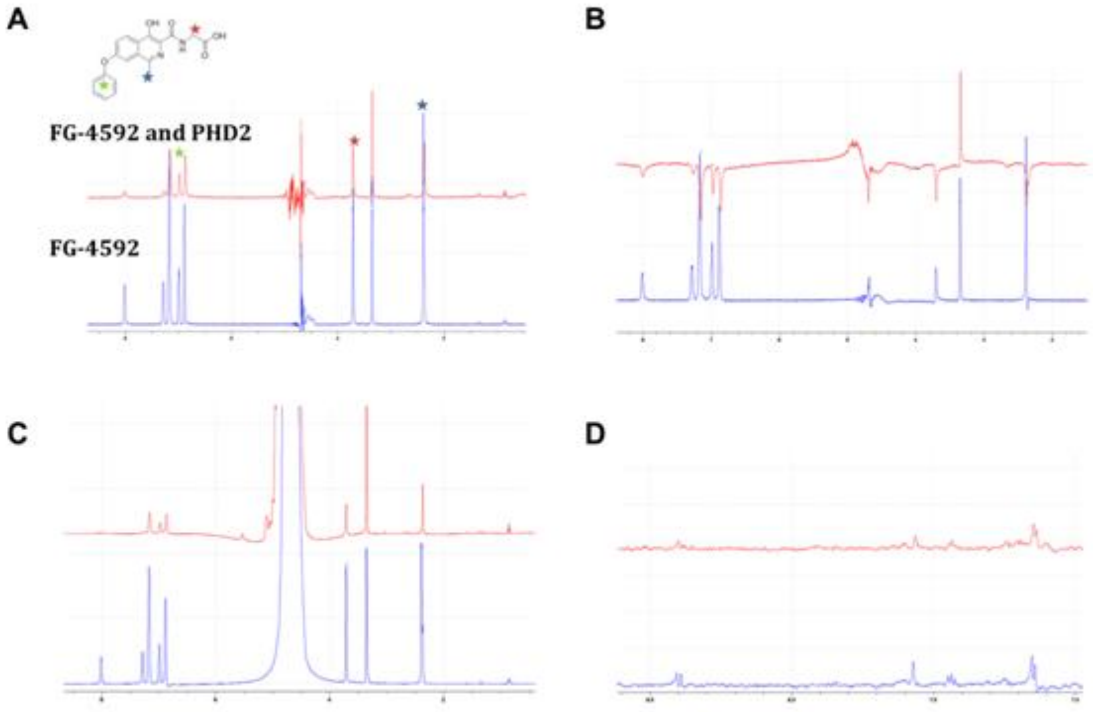

Fig. S6. NMR assay of FG-4592 binding to PHD2. (**A**) Single-concentration qualitative binding monitored by ^{1}H excitation sculpting NMR. Peaks corresponding to FG-4592 are highlighted by stars. Addition of FG-4592 to PHD2.Zn leads to line broadening of the FG-4592 peaks, indicating binding. (**B**) Single-concentration qualitative binding screening monitored by ^{1}H CPMG NMR. Addition of FG-4592 to PHD2 leads to a reduction in the intensity of the peaks of FG-4592, indicating binding. **(C**) wLOGSY NMR spectra of FG-4592 with PHD2.[6,7,9] The results imply FG-4592 binds to PHD2 since the protein and FG-4592 share similar intermolecular NOEs signs. (**A**-**C**) Assay mixtures contained 50 µM *apo*-PHD2$_{181\text{-}426}$, 200 µM Zn(II), 500 µM FG-4592 and 50 mM Tris-D$_{11}$, pH 7.5 in 10 % D_2O and 90 % $_2$O. (**D**) ^{1}H CPMG NMR screening of an equimolar enzyme-inhibitor mixture (50 µM) of FG-4592 and PHD2. The addition of FG-4592 to PHD2 leads to a partial reduction in the intensity of the peaks of FG-4592 with an equimolar mixture enzyme-inhibitor, characteristic of moderate to strong binding. Red curves: FG-4592 and PHD2; blue curves: FG-4592 alone.

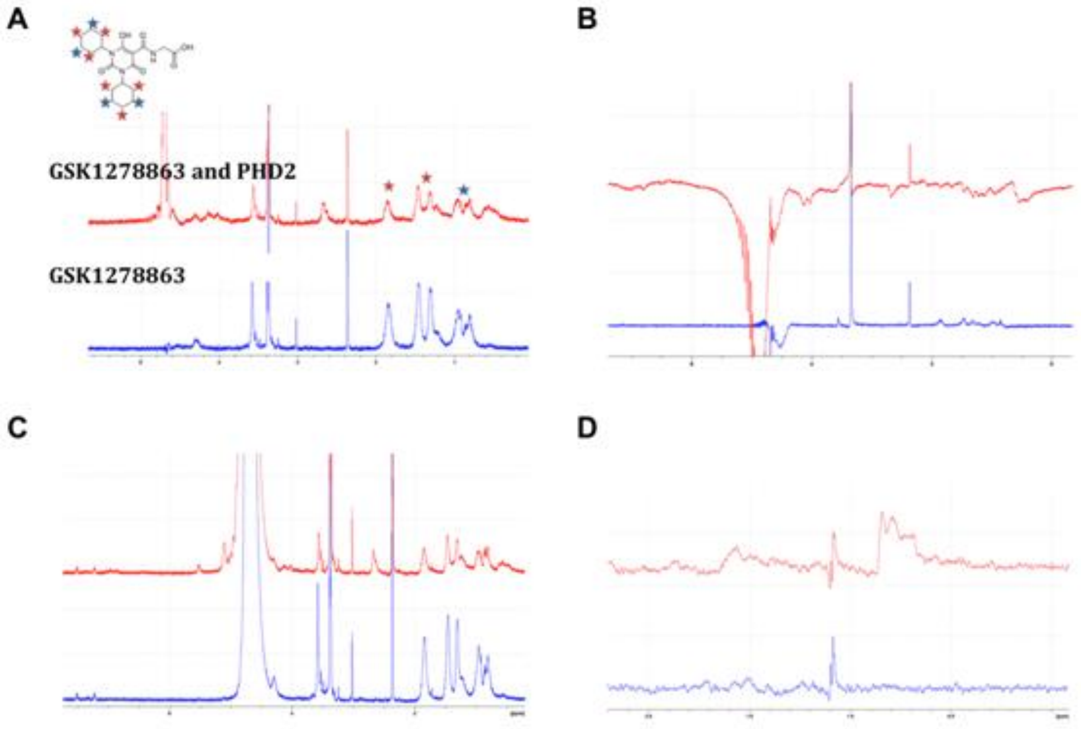

Fig. S7. NMR assay of GSK1278863 binding to PHD2. (**A**) Single-concentration qualitative binding screening monitored by ^{1}H excitation sculpting NMR. Peaks corresponding to GSK1278863 are highlighted with stars. Addition of GSK1278863 to PHD2.Zn leads to line broadening of the GSK1278863 peaks, indicating binding. (**B**) Single-concentration qualitative binding screening monitored by ^{1}H CPMG NMR. The addition of GSK1278863 to PHD2 leads to a reduction in peak intensity for GSK1278863, indicating binding. (**C**) wLOGSY NMR spectra of GSK1278863 with PHD2 PHD2.[6,7,9] The results imply that GSK1278863 binds to PHD2 since the protein and GSK1278863 share similar intermolecular NOEs signs. (**A**-**C**) Assay mixtures contained 50 µM *apo*-PHD2$_{181\text{-}426}$, 200 µM Zn(II), 500 µM GSK1278863 and 50 mM Tris-D$_{11}$, pH 7.5 in 10 % D_2O and 90 % H_2O. (**D**) ^{1}H CPMG NMR screening of an equimolar enzyme-inhibitor mixture (50 µM) of GSK1278863 and PHD2. The addition of GSK1278863 to PHD2 leads to a partial reduction in the intensity of the peaks of GSK1278863 with an equimolar enzyme-inhibitor mixture, characteristic of moderate to strong binding. Red curves: GSK1278863 and PHD2; blue curves: GSK1278863 alone.

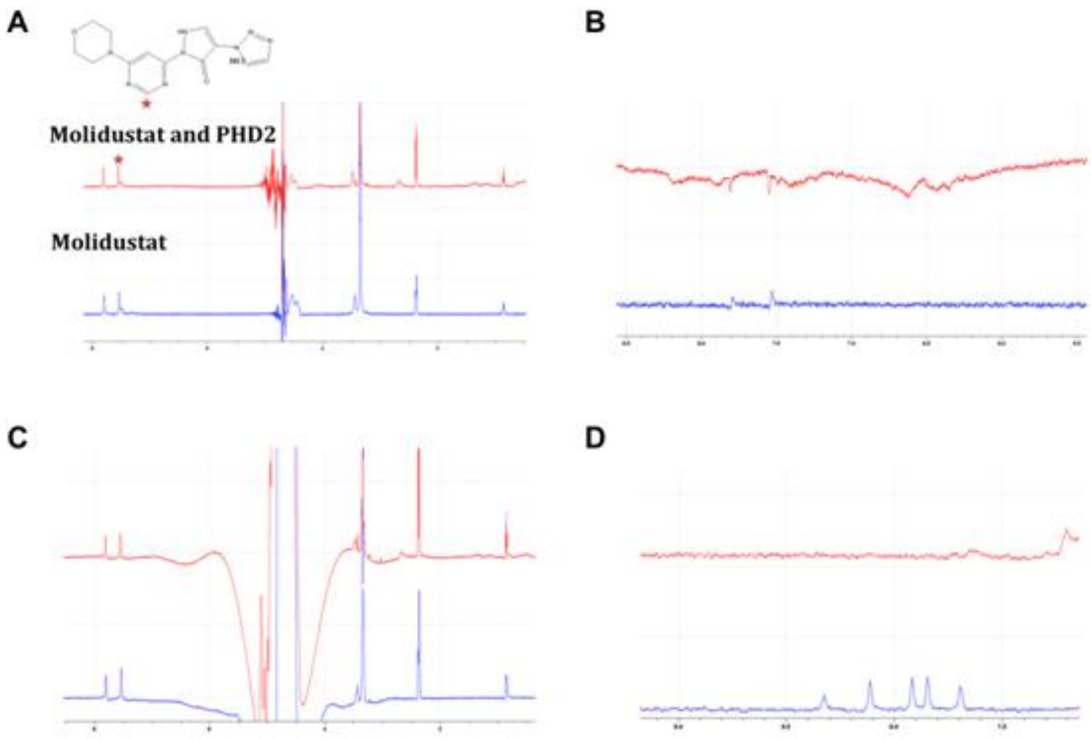

Fig. S8. NMR assay of Molidustat binding to PHD2. (**A**) Single-concentration qualitative binding screening monitored by ^{1}H excitation sculpting NMR. The peaks corresponding to Molidustat are highlighted with stars. Addition of Molidustat to PHD2.Zn leads to line broadening of the Molidustat peaks, indicating binding. (**B**) Single-concentration qualitative binding screening monitored by ^{1}H CPMG NMR. The addition of Molidustat to PHD2 leads to a reduction in the intensity of the peaks of Molidustat, indicating binding. (**C**) wLOGSY NMR spectra of Molidustat with PHD2 PHD2.[6,7,9] The results show that Molidustat binds to PHD2 since the protein and Molidustat share similar intermolecular NOEs signs. (**A-C**) Assay mixtures contained 50 µM *apo*-PHD2$_{181-426}$, 200 µM Zn(II), 500 µM Molidustat and 50 mM Tris-D$_{11}$, pH 7.5 in 10 % D_2O and 90 % H_2O. (**D**) ^{1}H CPMG NMR screening of an equimolar enzyme-inhibitor mixture (50 µM) of Molidustat and PHD2. The addition of Molidustat to PHD2 leads to complete reduction in the intensity of the peaks of Molidustat with an equimolar enzyme-inhibitor mixture, characteristic of strong binding. Red curves: Molidustat and PHD2; blue curves: Molidustat alone.

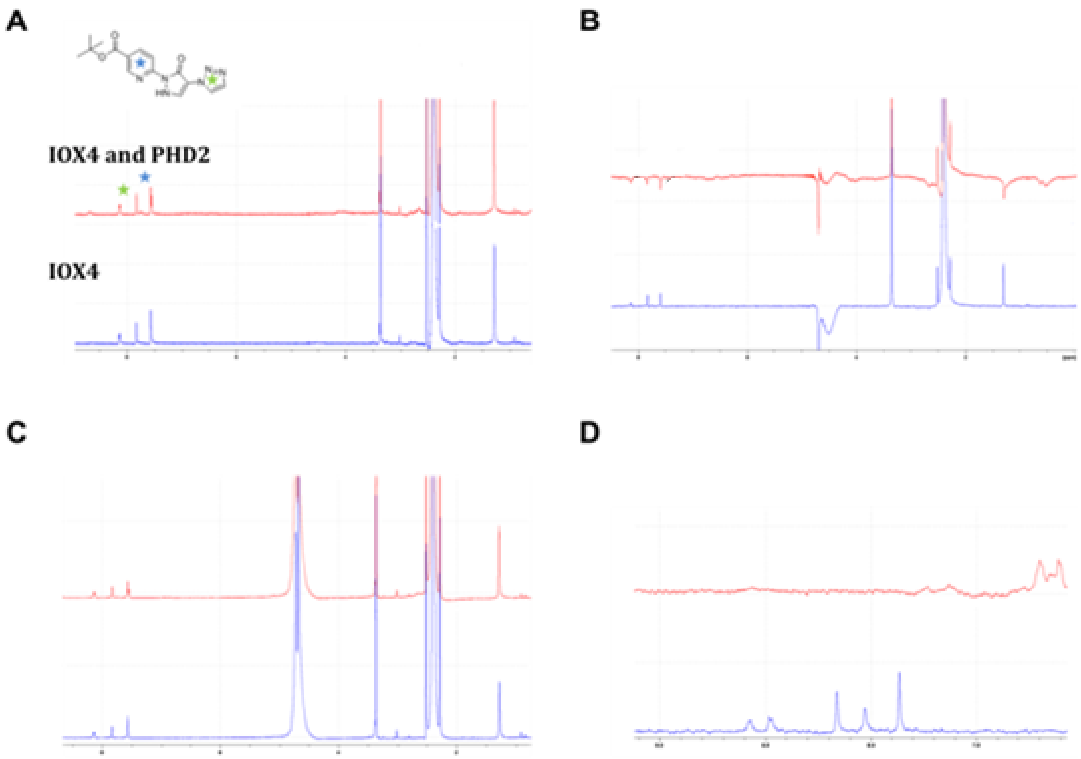

Fig. S9. NMR assay of IOX4 binding to PHD2. (**A**) Single-concentration qualitative binding screening monitored by ^1H excitation sculpting NMR. The peaks corresponding to IOX4 are highlighted with stars. Addition of IOX4 to PHD2.Zn leads to line broadening of the IOX4 peaks, indicating binding. (**B**) Single-concentration qualitative binding screening monitored by ^1H CPMG NMR. Addition of IOX4 to PHD2 leads to a reduction in the intensity of the peaks of IOX4, indicating binding. (**C**) wLOGSY NMR spectra of IOX4 with PHD2.[6,7,9] Data show that IOX4 binds to PHD2 since the protein and IOX4 share similar intermolecular NOEs signs. (**A-C**) Assay mixtures contained 50 µM *apo*-PHD2$_{181\text{-}426}$, 200 µM Zn(II), 500 µM IOX4 and 50 mM Tris-D_{11}, pH 7.5 in 10 % D_2O and 90 % H_2O. (**D**) ^1H CPMG NMR screening of an equimolar enzyme-inhibitor mixture (50 µM) of IOX4 and PHD2. The addition of IOX4 to PHD2 leads to a reduction in the intensity of the IOX4 peaks with an equimolar enzyme-inhibitor mixture, characteristic of strong binding. Red curves: IOX4 and PHD2; blue curves: IOX4 alone.

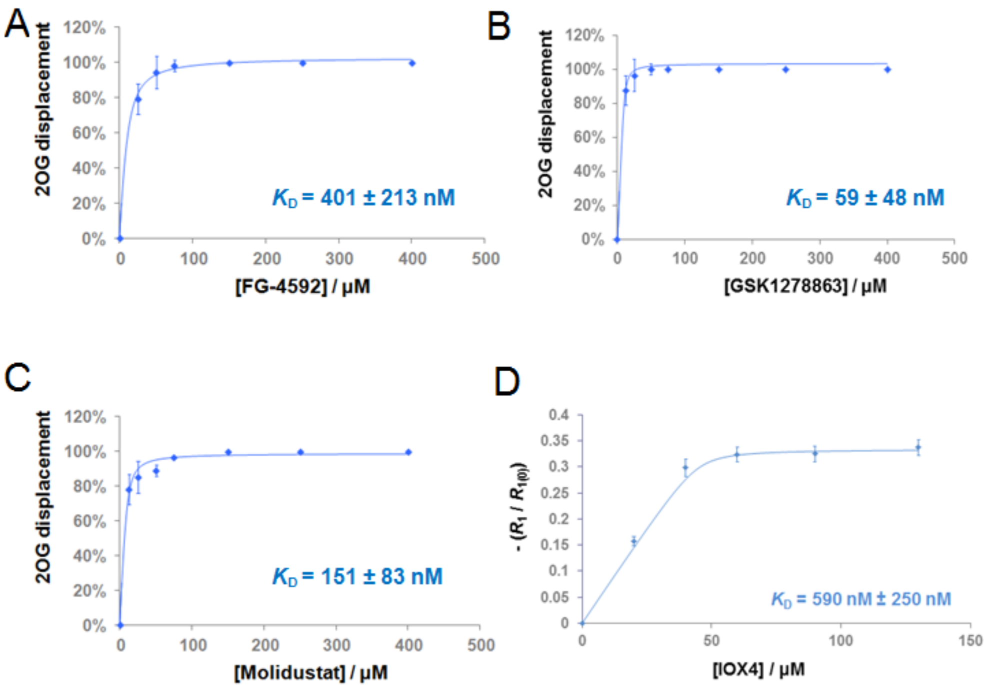

Fig. S10. Measurement of binding constants between PHD inhibitors and the catalytic domain of PHD2. (**A**-**C**) K_D curve fitting for FG-4592, GSK1278863, and Molidustat, respectively, as obtained by 2OG displacement by ^{1}H CPMG monitoring using PHD2.Zn(II) complex and 2OG as the reporter ligand.[5–7,9] Assay mixtures contained 10 μM *apo*-PHD2$_{181\text{-}426}$, 60 μM Zn(II), 10 μM 2OG, and increasing concerntrations of compound in 50 mM Tris-D11, pH 7.5 in 10 % D_2O and 90 % H_2O. (**D**) K_D curve fitting for IOX4 as obtained by the water relaxation method.[9]

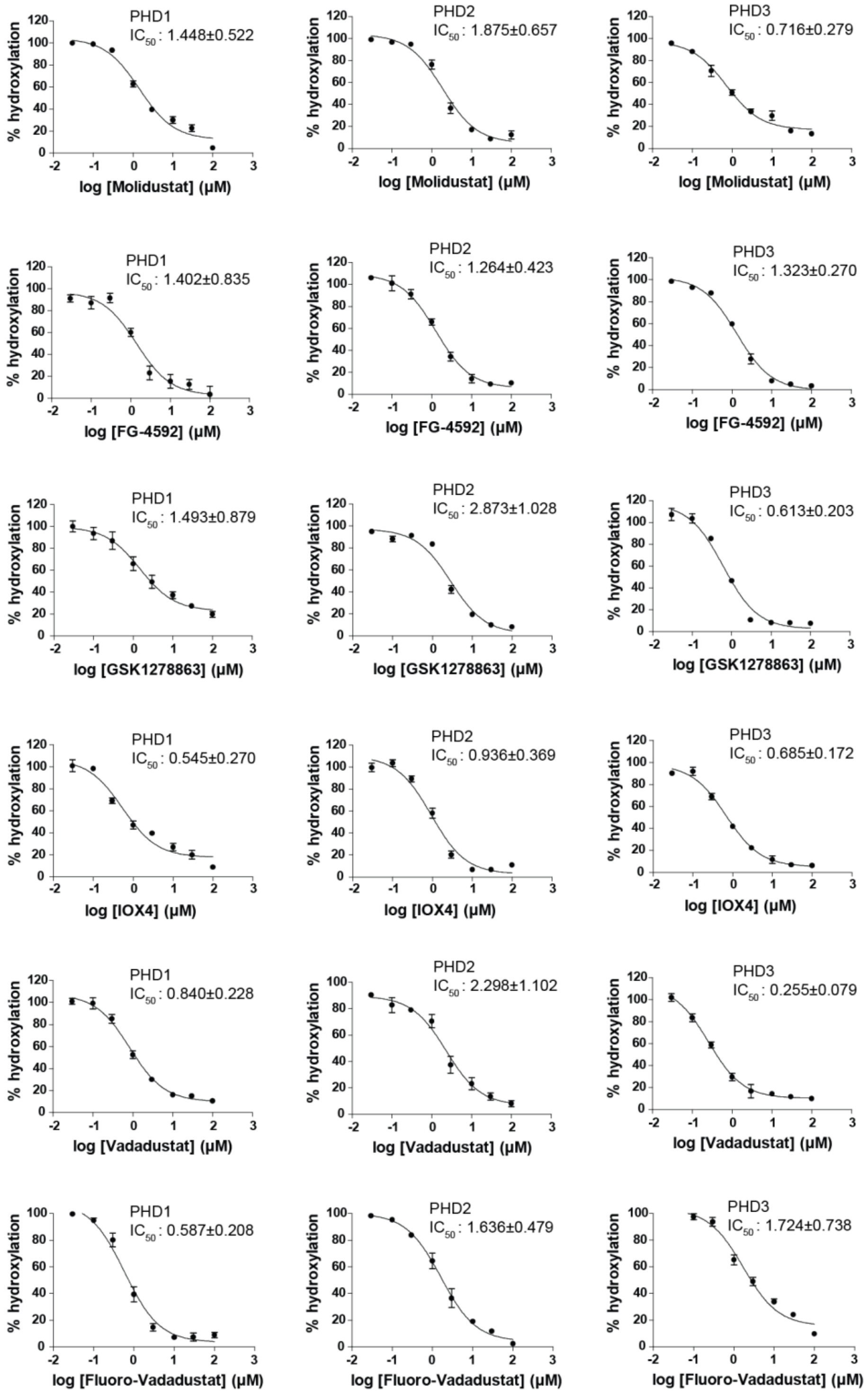

Fig. S11. *In vitro* PHD isoform selectivity profiling of PHD inhibitors. Inhibition of PHD inhibitors against human PHD1-3 as assayed by MALDI-TOF MS (the protocol is described in Chan *et al.*[25]). Eight concentrations were used to obtained dose-response curve for each compound. Data points presented in the graph are mean with standard deviation, n=3. IC_{50} values (listed in Supplemental Table S3) were calculated using GraphPad Prism® 5.04.

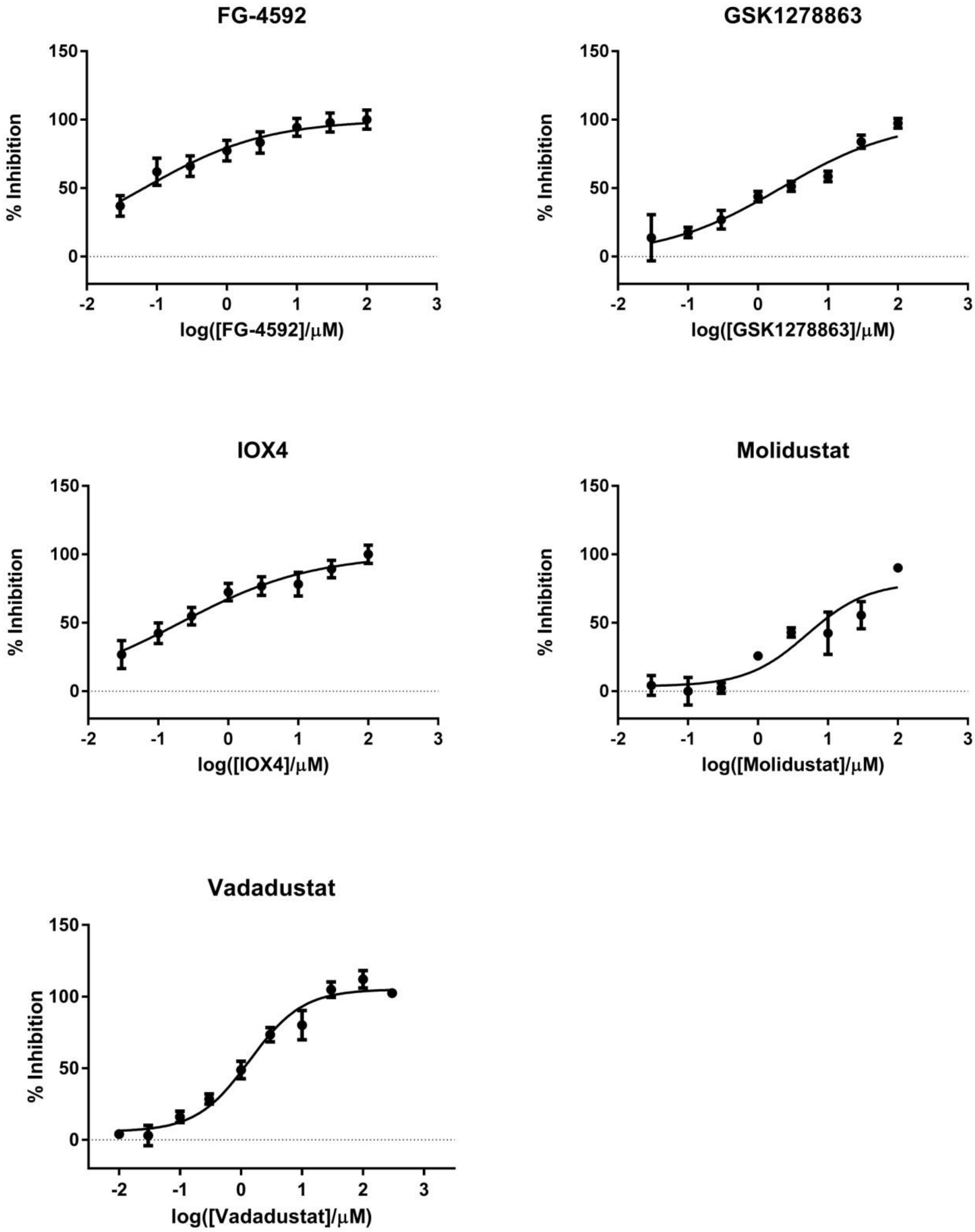

Fig. S12. *In vitro* OGFOD1 selectivity profiling of PHD inhibitors. Inhibition of PHD inhibitors against human OGFOD1 as assayed by MALDI-TOF MS (the protocol is described in the Supplemental Materials and Methods section).[11,13] No enzyme and DMSO controls were used for the normalisation of RPS23 peptide hydroxylation. Eight concentrations were used to obtained dose-response curve for each compound. Data points presented in the graph are means with standard deviation, n=3. IC_{50} values (listed in Table 2) were calculated using GraphPad Prism® 5.04.

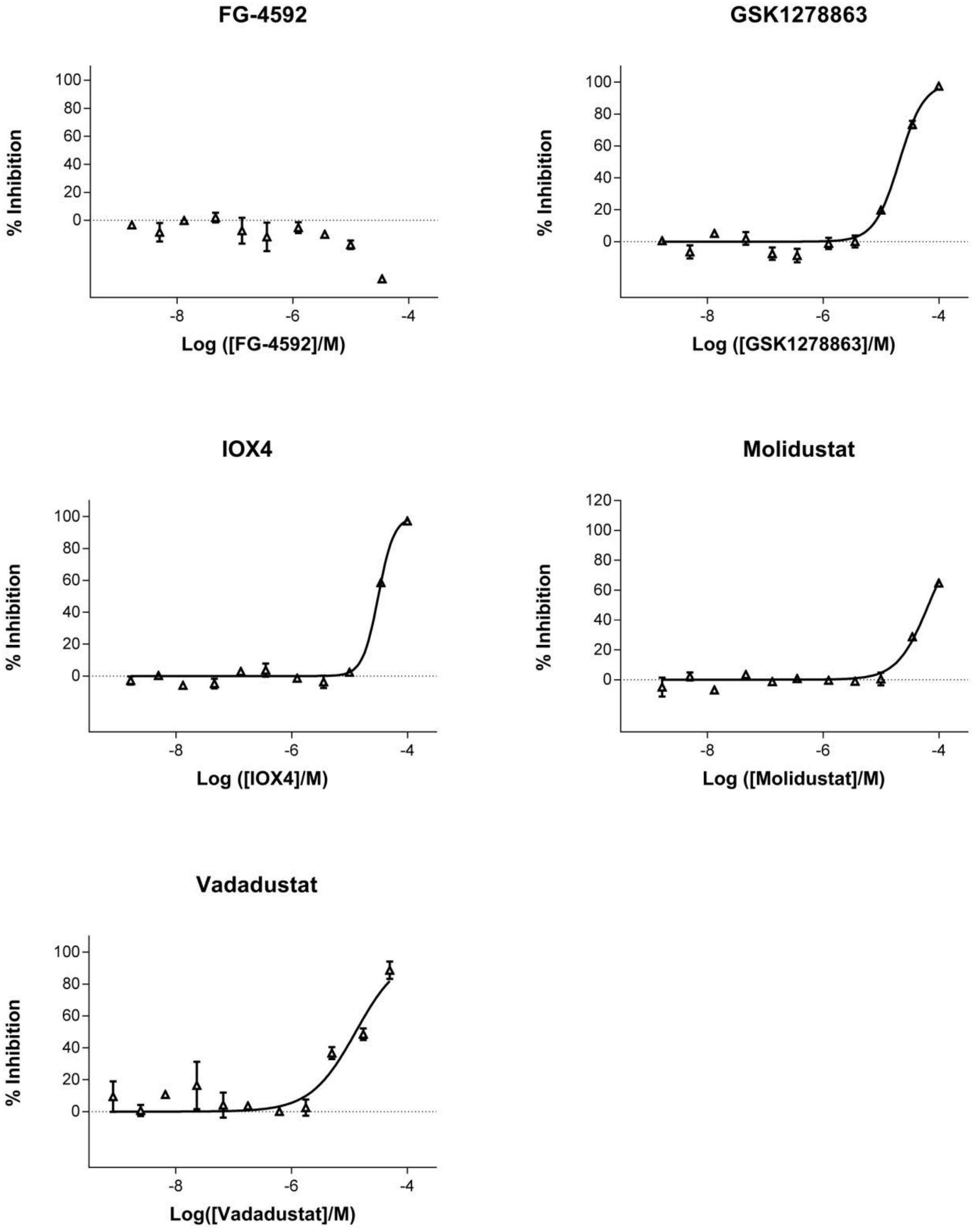

Fig. S13. *In vitro* FIH selectivity profiling of PHD inhibitors. Inhibition of PHD inhibitors against FIH as assayed by RapidFire-MS (the protocol is described in the Supplementary Materials and Methods section). Eleven concentrations assayed in duplicate were used to obtained dose-response curve for each compound. Values present in the graph are means with standard deviation, n=2. IC_{50} values were calculated using GraphPad Prism® 5.04.

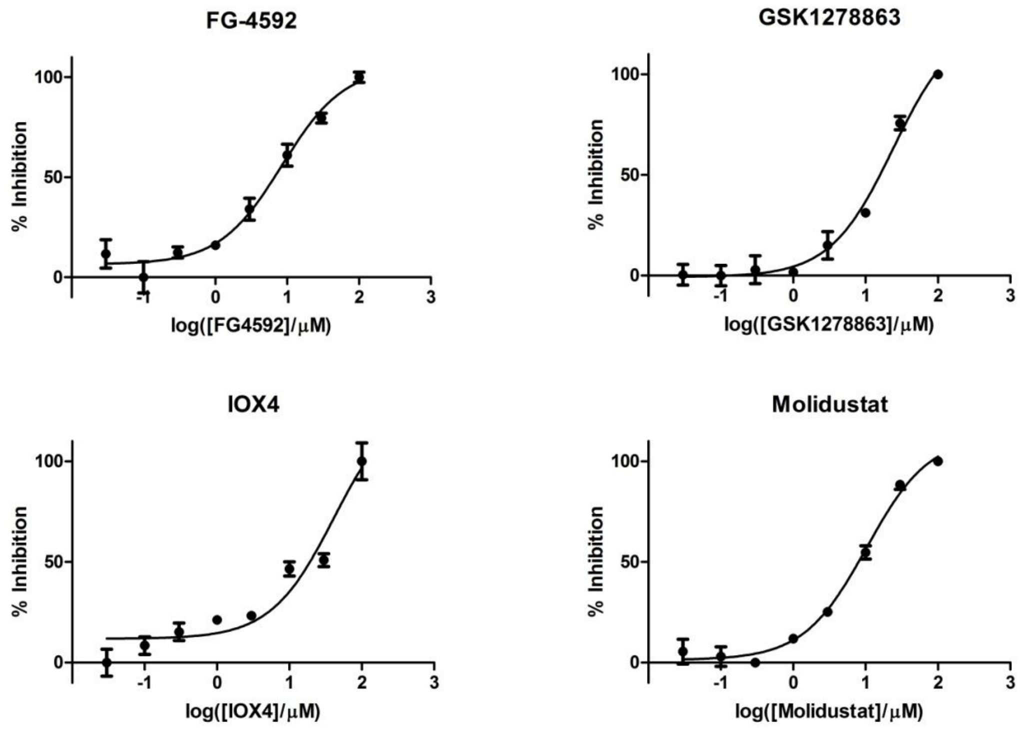

Fig. S14. *In vitro* OFD1 selectivity profiling of PHD inhibitors. Inhibition of PHD inhibitors against Schizosaccharomyces pombe OFD1 as assayed by MALDI-TOF MS (the protocol is described in the Supplementary Materials and Methods section). No enzyme and DMSO controls were used for normalisation of RPS23 peptide hydroxylation. Eight concentrations were used to obtained dose-response curve for each compound. Data points presented in the graph are means with standard deviation, n=3. IC_{50} values (listed in Table 2) were calculated using GraphPad Prism® 5.04.

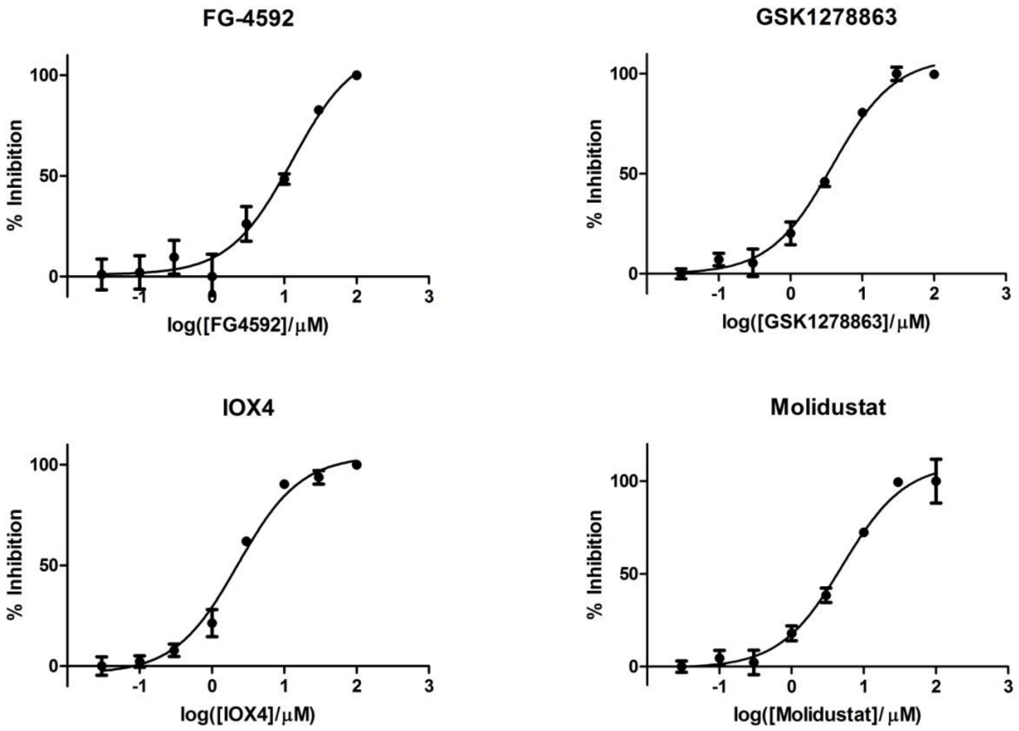

Fig. S15. *In vitro* TPA1p selectivity profiling of PHD inhibitors. Inhibition of PHD inhibitors against *Saccharomyces cerevisiae* TPA1p as assayed by MALDI-TOF (the protocol is described in the Supplementary Materials and Methods section). No enzyme and DMSO controls were used for normalisation of RPS23 peptide hydroxylation. Eight concentrations were used to obtained dose-response curve for each compound. Data points presented in the graph are means with standard deviation, n=3. IC_{50} values (listed in Table 2) were calculated using GraphPad Prism® 5.04.

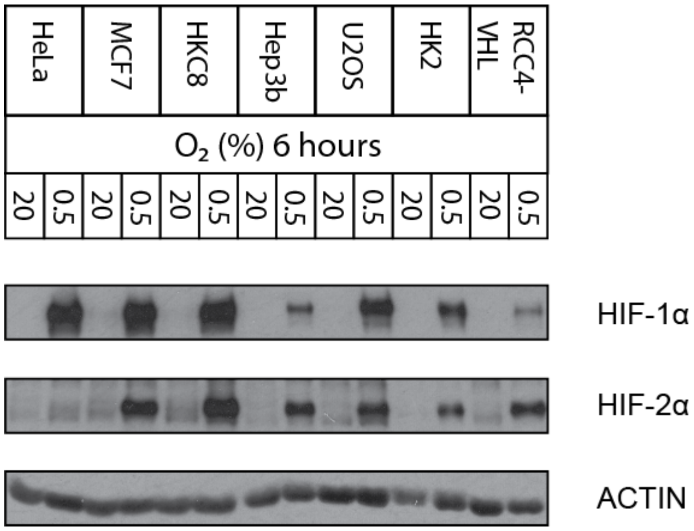

Fig. S16. Immunoblot showing comparison of HIF-1α and HIF-2α expression in various cell lines. HeLa, MCF7, HKC8, Hep3b, U2OS, HK2 and RCC4-VHL cells were incubated in normoxic levels (i.e. 20% O_2) and hypoxic condition (0.5% O_2) for 6 hours.

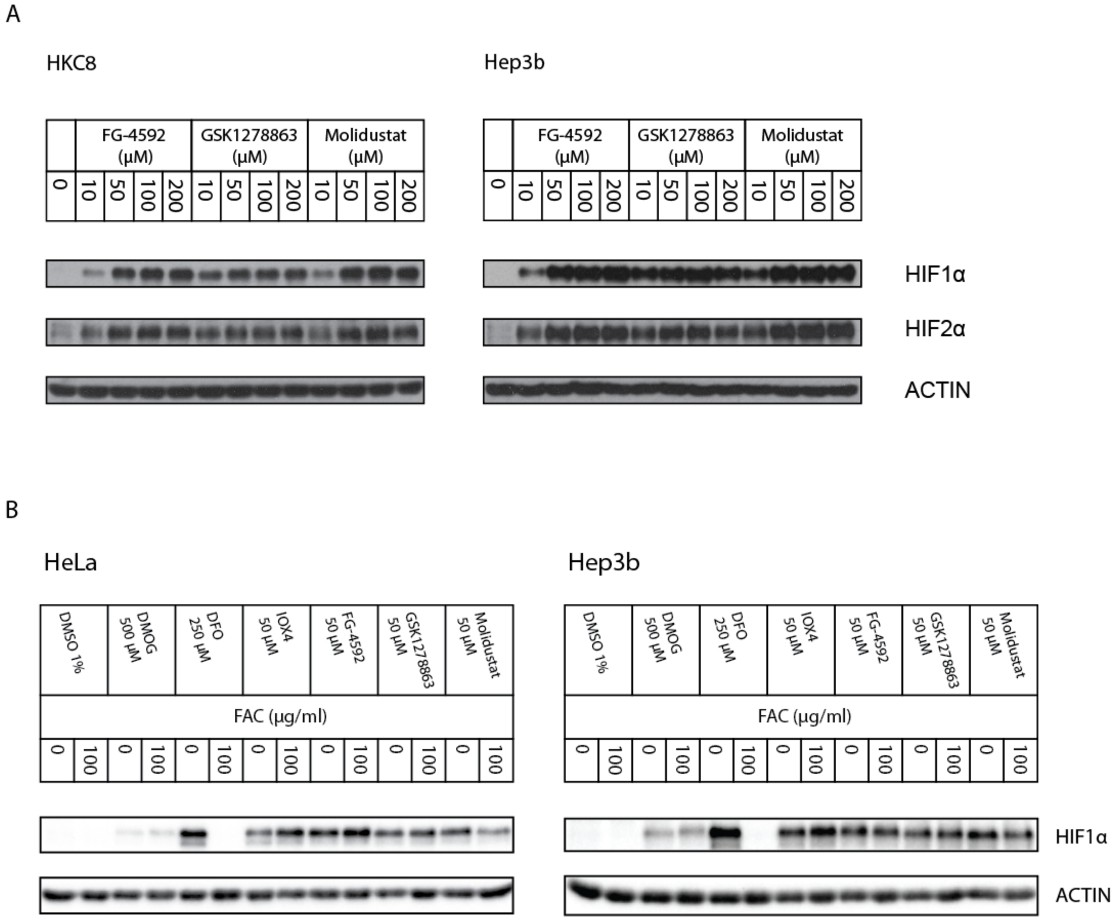

Fig. S17. Comparison of PHD inhibitors on HIF-α stabilization in human cell lines. (**A**) Comparison of the effects of FG-4592, GSK1278863 and Molidustat on HIF1-α and HIF2-α stabilization in HKC8 and Hep3b cells. (**B**) Fe(II) titration in the presence of PHD inhibitors. Ferrous ammonium citrate (FAC) was added to HeLa and Hep3b cells pre-treated with PHD inhibitors, including DMOG, DFO, IOX4, FG-4592, GSK1278863 and Molidustat. Addition of Fe(II) completely abolished observed HIF1-α stabilization in iron-chelator (DFO) treated cells whereas HIF1-α remained stabilized after administration of Fe(II) in cells treated with the other inhibitors. Consistent with the biophysical analyses, these observations suggest that the mechanism of inhibition is not (at least predominantly) through iron chelation in solution by these inhibitors.

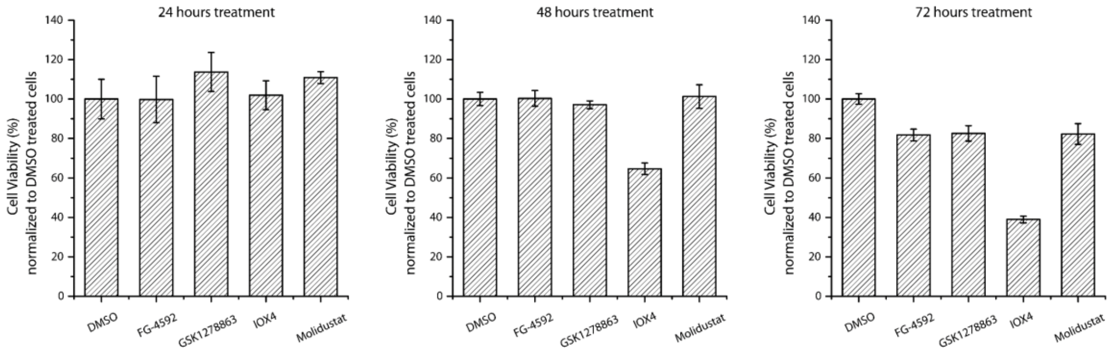

Fig. S18. Hep3b cell viability after prolonged treatment of PHD inhibitors. Cell viability normalized to DMSO treated sample after A 24 hours, B 48 hours and C 72 hours treatment with10 μM FG-4592, GSK1278863, IOX4, or Molidustat. Note that prolonged treatment of IOX4 caused a significant reduction in cell viability.

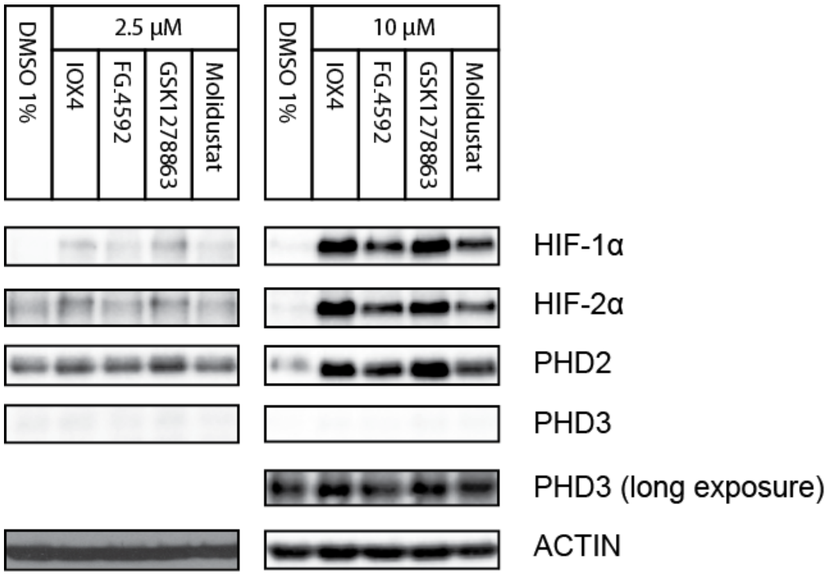

Fig. S19. Immunoblots of Hep3b cells treated with 2.5 μM and 10 μM PHD inhibitors for 24 hours. The blots show protein levels of HIF-1α, HIF-2α, PHD2, PHD3 and β-actin at the 24th hour. The PHD3 blots show no evidence of PHD3 protein induction after inhibitor treatment (10 μM; Signal after 30sec application of the SuperSingal West Dura reagent); longer exposure of PHD3 (10 μM; signal after 36sec application of the SuperSignal West Femto reagent; typically it takes 9 times incubation time with Dura to match the signal level detected with Femto for PHD3) implies the low/basal expression levels for PHD3 after inhibitor treatment.

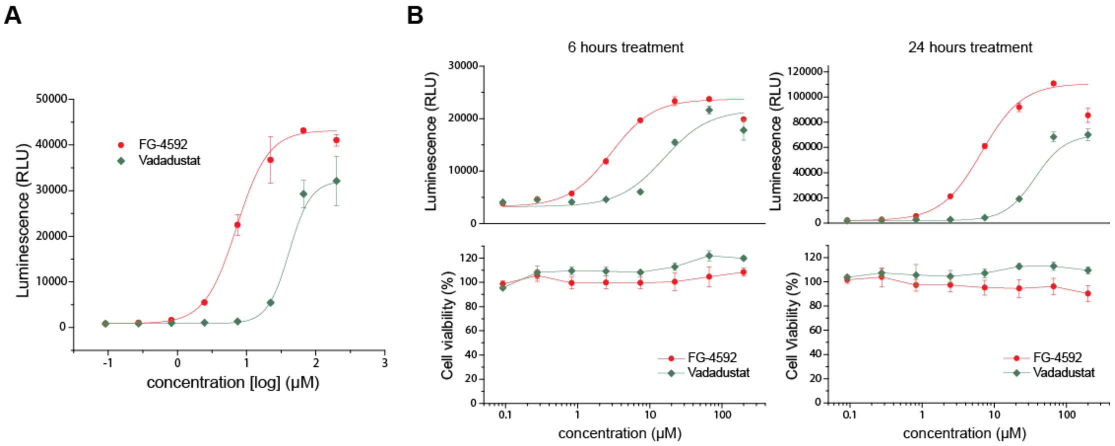

Fig. S20. Effects of Vadadustat on Cellular Inhibition assessed by the HRE luciferase reporter assay. (**A**) HIF-α induction in HT1080 cells 16 hours following PHD inhibitor treatment as measured by the HRE luciferase reporter assay. The dose-response curve for Vadadustat substantially lags behind that for FG-4592 suggesting that under these conditions Vadadustat is a weaker inhibitor (EC_{50} FG-4592: 7 μM , Vadadustat: 41 μM). (**B**) Dose response of HIF-α stabilization and on cell viability after 6 hours and 24 hours inhibitor treatments. 6 hours treatment EC_{50} FG-4592: 3 μM, Vadadustat: 15.7 μM; 24 hours treatment EC_{50} FG-4592: 7 μM , Vadadustat: 37 μM.

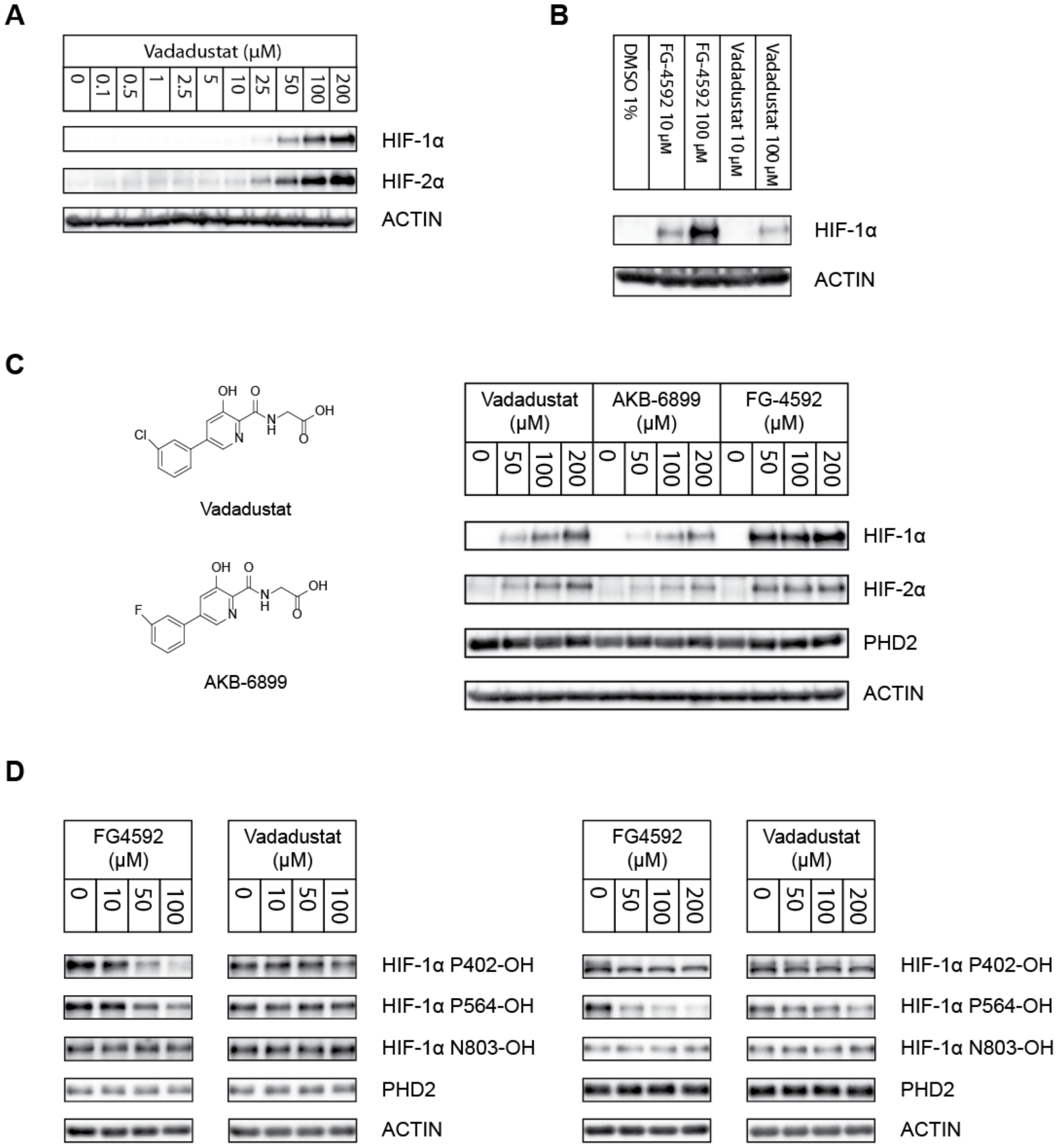

Fig. S21. Effects of Vadadustat on Cellular Activity of the HIF prolyl- and asparaginyl-hydroxylases. (**A**) Immunoblots showing dose-dependent HIF-1α and HIF-2α stabilization in Hep3b cells after 6 hours treatment with Vadadustat. (**B**) Structure of Vadadustat and its fluorinated derivative, AKB-6899. Comparison of the effects of FG-4592 and Vadadustat on HIF1-α stabilization in Hep3b cells after 6 hour treatment with the PHD inhibitors. (**C**) Comparison of the effects of FG-4592, Vadadustat and AKB-6889 on HIF1-α and HIF2-α stabilization in Hep3b cells after 6 hours treatment of PHD inhibitors. (**D**) Comparison of inhibitor potency on Pro hydroxylation of NODD and CODD sites and on Asn hydroxylation between FG-4592 and Vaddadustat at various concentrations in RCC4 cells after 6 hour treatment with the PHD inhibitors.

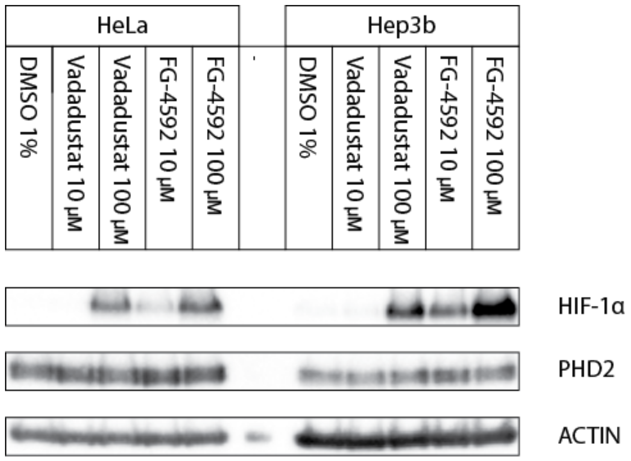

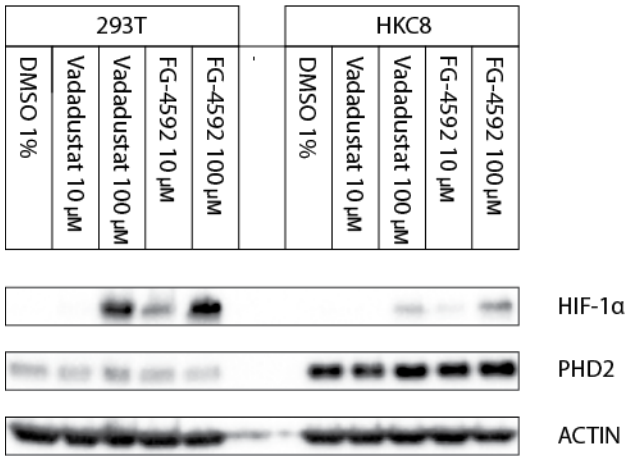

Fig. S22. Effects of Vadadustat on HIF-1α stabilization in different cell lines. Immunoblots showing HIF-1α stabilization after 7 hour treatment of FG-4592 or Vadadustat in HeLa, Hep3b, 293T and HKC8 cells. Protein levels of PHD2 and corresponding HIF-1α show that the level of PHD2 varies in cell lines and therefore the degree of PHD inhibitor's effect on HIF-1α stabilization. (Blots of the same antibody were incubated at the same time.)

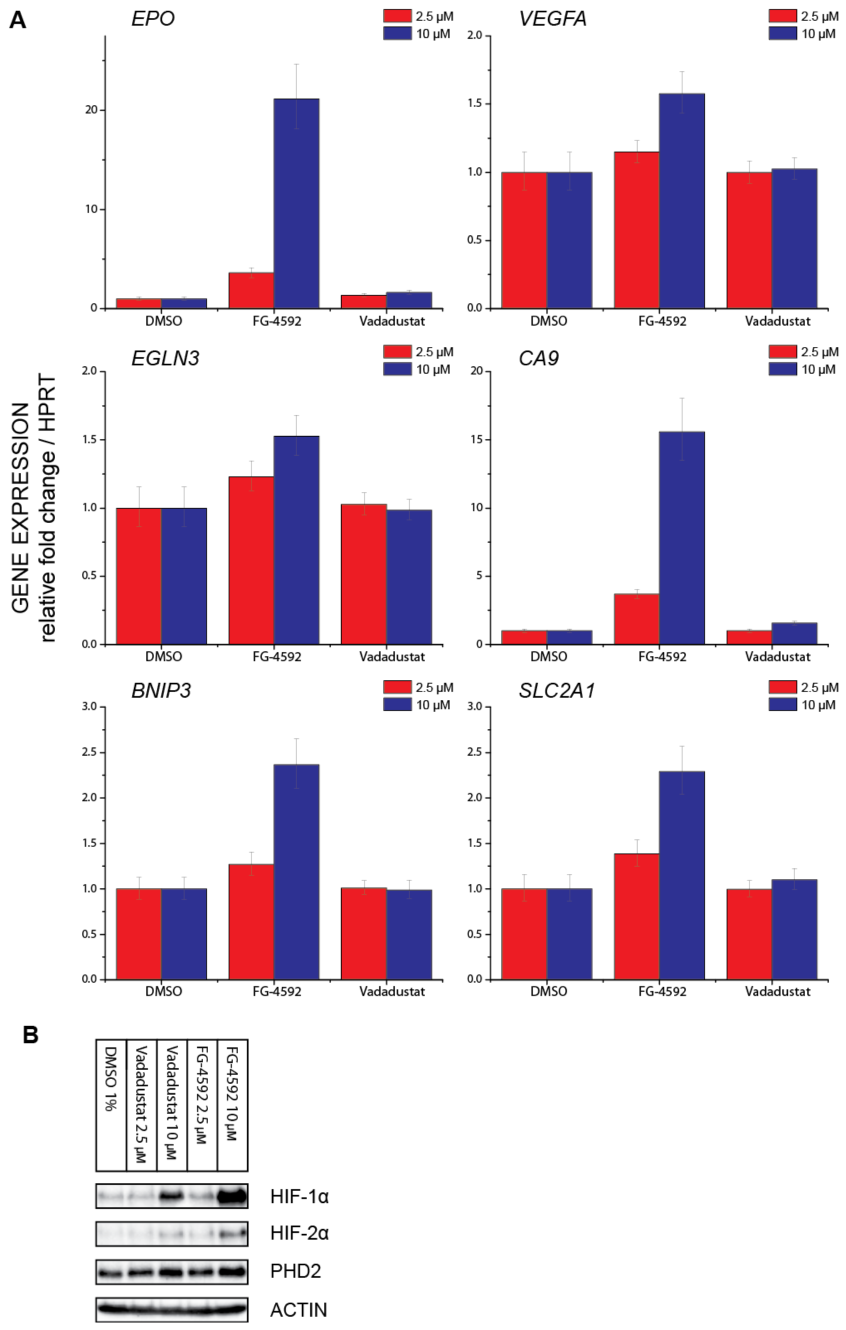

Fig. S23. Effects of Vadadustat on HIF target gene transcription (with FG-4592 for comparison). (**A**) Regulation of HIF target genes, i.e. HIF-1α dependent cabonic anhydrase IX (*CA9*) and BCL2/adenovirus E1B 19 kDa protein-interacting protein 3 (*BNIP3*), HIF-2α dependent erythropoietin (*EPO*), both HIF-1α and HIF-2 α dependent glucose transporter 1 (*SLC2A1*) and vascular endothelial growth factor (*VEGFA*), and the strongly FIH dependent HIF target gene PHD3 (*EGLN3*), after treatment with 2.5 µM or 10 µM Vadadustat, or FG-4592 for 24 hours, as assayed by RT-qPCR. Each data point represents the mean with standard deviation of the relative fold change with respect to DMSO treated sample normalized to reference gene HPRT level, n=3 biological repeats for inhibitors and n=4 for DMSO control. (**B**) Immunoblots of cells treated with 2.5 µM or 10 µM Vadadustat, or FG-4592 for 24 hours showing level of HIF-1α, HIF-2α and PHD2 protein level at the 24th hour. Cells were seeded and treated with inhibitors at the same time of the experiments shown in A. Note that an overall correlation between the HIF-α protein level and HIF-α target gene response is observed.

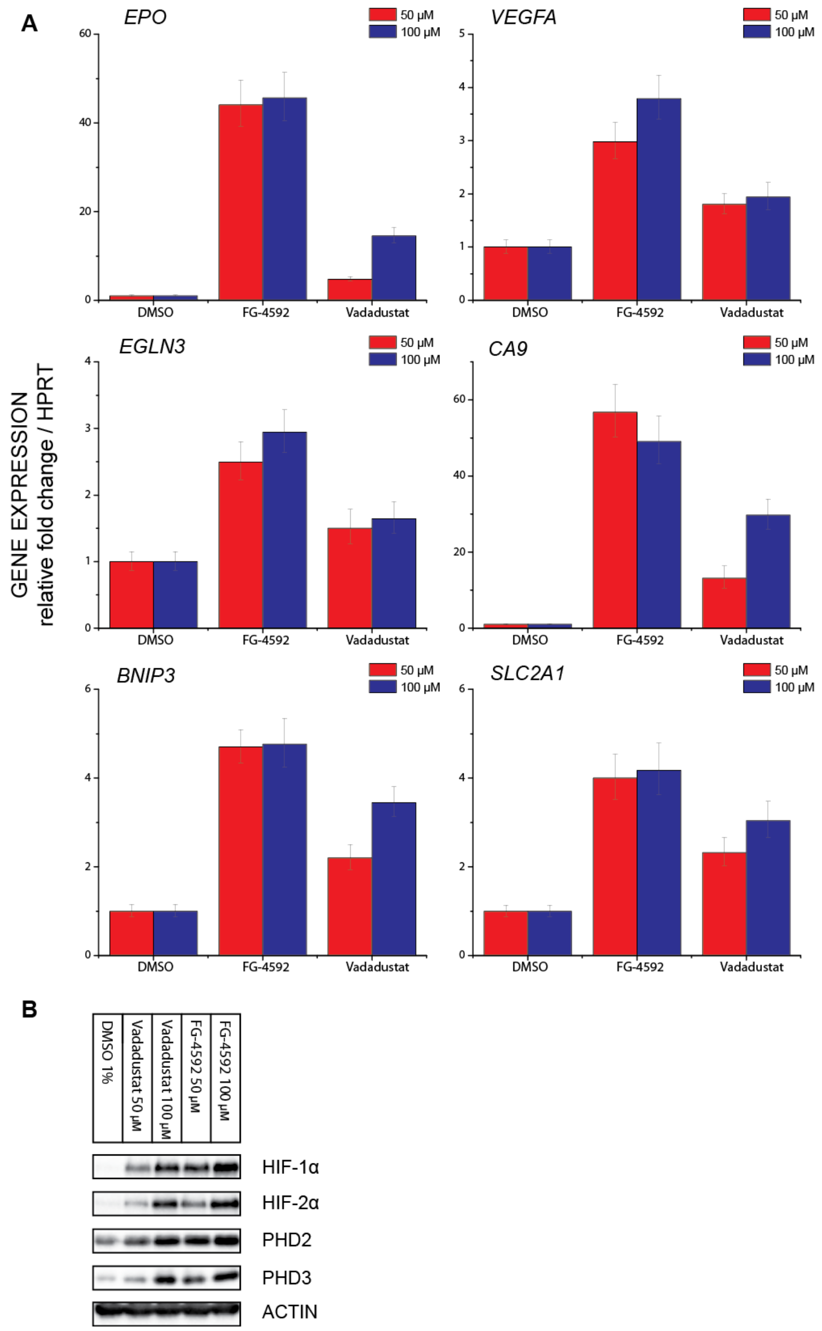

Fig. S24. Effects of Vadadustat on HIF target gene transcription (with FG-4592 for comparison). (**A**) Regulation of HIF target genes, i.e. HIF-1α dependent cabonic anhydrase IX (*CA9*) and BCL2/adenovirus E1B 19 kDa protein-interacting protein 3 (*BNIP3*), HIF-2α dependent erythropoietin (EPO), both HIF-1α and HIF-2 α dependent glucose transporter 1 (*SLC2A1*) and vascular endothelial growth factor (*VEGFA*), and the strongly FIH dependent gene PHD3 (*EGLN3*), after treatment with 50 µM or 100 µM Vadadustat or FG-4592 for 24 hours, as assayed by RT-qPCR. Each data point represent the mean and standard deviation of the relative fold change with respect to DMSO treated sample normalized to reference gene *HPRT* level, n=3 biological repeats for inhibitors and n=4 for DMSO control. (**B**) Immunoblots of cells treated with 50 µM or 100 µM Vadadustat, or FG-4592 for 24 hours showing level of HIF-1α, HIF-2α, PHD2 and PHD3 protein level at the 24th hour. Cells were seeded and treated with inhibitors at the same time of the experiments shown in A. Note that an overall correlation between the HIF-α protein levels and HIF-α target gene response is observed.

Supplementary Tables

Table S1. Data collection and refinement statistics of FIH and PHD2 complex structures.

	**FIH** Zn(II)/GSK1278863	**FIH** Zn(II)/Molidustat -	**FIH** Zn(II)/Vadadustat	**PHD2** Mn(II)/Vadadustat	**PHD2** Mn(II)/CCT6
**PDB Code**	**5OP6**	**5OP8**	**5OPC**	**5OX6**	**5OX5**
**Data Collection**					
Beamline (Wavelength, Å)	DLS I04 (0.9795)	DLS I04 (0.9795)	DLS I03 (0.9763)	DLS I02 (0.9762)	DLS I02 (0.9800)
Detector	PILATUS 6M-F	PILATUS 6M-F	PILATUS 6M-F	PILATUS 6M-F	PILATUS 6M-F
Data Processing	MOSFLM[26]/SCALA[27]	MOSFLM[26] /SCALA[27]	MOSFLM[26] /SCALA[27]	HKL2000[28]	HKL2000[28]
Space group	$P4_12_12$	$P4_12_12$	$P4_12_12$	$P6_3$	$P6_3$
Cell dimensions					
a,b,c (Å)	86.2, 86.2, 147.1	86.2, 86.2, 148.2	86.8, 86.8, 149.5	111.1, 111.1, 40.0	110.1, 110.1, 39.4
α,β,γ(°)	90.0, 90.0, 90.0	90.0, 90.0, 90.0	90.0, 90.0, 90.0	90, 90, 120	90.0, 90.0, 120.0
No. reflections	21126 (3014)*	25593 (3657)*	26216 (3748)*	19366 (1883)*	13170 (1314)*
Resolution (Å)	30.48-2.45 (2.58-2.45)*	30.47-2.30 (2.41-2.30)	86.80-2.30 (2.42-2.30)*	36-36-1.99 (2.06-1.99)*	47.69-2.25 (2.33-2.25)*
R_{sym} or R_{merge}**	0.138 (1.253)*	0.148 (1.229)*	0.152 (1.834)*	0.108 (1.076)*	0.138 (0.953)*
I/σI	11.5 (2.0)*	8.9 (1.9)*	10.9 (1.8)*	14.8 (1.5)*	12.9 (2.1)*
CC 1/2	0.998 (0.570)*	0.995 (0.695)*	0.998 (0.689)*	0.996 (0.438)*	0.994 (0.598)*
Completeness (%)	100 (100)*	99.5 (100)*	100 (100)*	98.2 (98.0)*	100 (99.9)*
Redundancy	11.5 (11.1)*	11.3 (11.5)*	17.5 (17.5)*	6.5 (6.4)*	5.7 (5.7)*
Wilson B value ($Å^2$)	52.45	48.87	55.78	33.77	40.16
**Refinement**	REFMAC[29]/ PHENIX[30]	REFMAC[29]/ PHENIX[30]	REFMAC[29]/ PHENIX[30]	CNS[31]/PHENIX[30]	CNS[31]/PHENIX[30]
$R_{work}/R_{free†}$	0.2037/0.2274	0.1992/0.2142	0.1925/0.2140	0.1743/0.2053	0.1747/0.2026
No. atoms					
- Enzyme	2619	2664	2697	1765	1676
- Ligand	28	23	21	21	18
- Water	46	62	72	127	85
Average B-factors					
- Enzyme	66.5	71.7	74.0	48.9	54.3
- Ligand	67.4	109.1	93.1	33.0	29.3
- Water	54.9	60.9	63.1	54.8	51.4
R.m.s deviations					
-Bond lengths (Å)	0.005	0.012	0.003	0.010	0.003
-Bond angles (°)	0.569	0.664	0.522	1.027	0.553

* Highest resolution shell in parenthesis ** Rsym = $\Sigma|I-\langle I\rangle|/\ \Sigma I$, where *I* is the intensity of an individual measurement and <I> is the average intensity from multiple observations †Rfactor= $\Sigma hkl||F\text{obs}(hkl)|-k|F\text{calc}(hkl)||/\Sigma hkl|F\text{obs}(hkl)|$, Rfree is the Rfactor for ~5%of reflections excluded from the refinement.

Table S2. Buffer and vapour diffusion condition used for FIH and PHD2 complex crystallization.

Protein complex	Sample composition	crystallization conditions	Vapour diffusion conditions
**FIH** Zn(II)/GSK1278863	11 mg/mL FIH (0.27 mM) in 50 mM Tris-HCl pH = 7.5, 4% DMSO, zinc acetate (0.5 mM), GSK1278863 (0.74 mM)	0.1 M HEPES pH = 7.5, 1.7 M ammonium sulphate, 5.0% PEG400	Sitting drop (300nL) protein-to-well ratio 1:2, 293K
**FIH** Zn(II)/Molidustat	11 mg/mL FIH (0.27 mM) in 50 mM Tris-HCl pH = 7.5, 4% DMSO, zinc acetate (0.5 mM), Molidustat (1.92 mM)	0.1 M HEPES pH = 7.5, 1.7 M ammonium sulphate, 5.5% PEG400	Sitting drop (300nL) protein-to-well ratio 1:2, 293K
**FIH** Zn(II)/Vadadustat	11 mg/mL FIH (0.27 mM) in 50 mM Tris-HCl pH = 7.5, zinc acetate (0.5 mM), Vadadustat (1.92 mM)	0.1 M HEPES pH = 7.5, 1.5 M ammonium sulphate, 5.5% PEG400	Sitting drop (300nL) protein-to-well ratio 1:2, 293K
**PHD2** Mn(II)/Vadadustat	27 mg/mL PHD2 (1 mM) in 50 mM Tris-HCl pH = 7.5, $MnCl_2$ (1.2 mM); Vadadustat (2mM)	0.1 M sodium acetate trihydrate pH 4.6, 0.2 M ammonium sulfate, 25% w/v PEG 4000	Sitting drop (300nL) protein to well ratio 2:1, 293K
**PHD2** Mn(II)/CCT6	27 mg/mL PHD2 (1 mM) in 50 mM Tris-HCl pH = 7.5, $MnCl_2$ (1.2 mM); CCT6 (2mM)	1.5 M sodium citrate tribasic dehydrate pH = 6.5	Sitting drop (200nL) protein-to-well ratio 1:1, 293K

Table S3. Selectivity of FG-4592, GSK1278863, Molidustat, IOX4, Vadadustat and Fluoro-Vadadustat against 1μM isolated recombinant human PHD1-3 using the MALDI-TOF MS based assay.[25]

	**IC_{50} (μM)**		
**Inhibitor**	**PHD1**	**PHD2**	**PHD3**
Molidustat	1.45	1.85	0.72
FG-4592	1.40	1.26	1.32
GSK1278863	1.50	2.87	0.61
IOX4	0.55	0.94	0.69
Vadadustat	0.84	2.30	0.26
AKB-6899	0.59	1.64	1.72

Table S4. *In vitro* activity of clinical trial PHD inhibitors against 150 nM isolated recombinant human PHD2 using the LC-MS based RapidFire assay.

**Inhibitor**	**PHD2**<br>**IC_{50} (μM)**
Molidustat	1.32
FG-4592	2.76
GSK1278863	0.09
IOX4	0.54
Vadadustat	1.33

Table S5. Published phase 2 studies of PHD inhibitors in Anaemia CKD.

Identifier	Location/Subjects	Study Design	Size	Dosage	Outcome*
		**Vadadustat (AKB-6548)**			
NCT01381094[32]	US; patients with anaemia secondary to CKD stage 3 or 4	Phase 2a; 6 weeks; randomized, double-blind, placebo-controlled	93	240, 370, 500, and 630 mg, daily	Hb increase ≥ 1 g/dL from baseline: 31%, 40%, 65% and 78% in 240, 370, 500 and 630 mg daily Vadadustat treatment, respectively, versus 11% in placebo arm
NCT01906489[33]	US; patients with non-dialysis dependent (NDD) CKD (stage 3a/b, 4, or 5)	Phase 2b; 20 weeks; randomized, double-blind, placebo-controlled	210	Starting 450 mg daily titrated by 1 tablet(150 mg); max 600mg and min 150 mg	Hb increase ≥ 1.2 g/dL from baseline or mean Hb ≥ 11.0 g/dL during last 2 weeks of treatment: 55% of Vadadustat treated subjects versus 10% in placebo arm
		**Roxadustat(FG-4592)**			
NCT00761657[34]	US; NDD-CKD subjects with Hb ≤ 11.0 g/dL	Phase 2a; 4 weeks (+12 weeks follow-up); randomized, single-blind, placebo-controlled	116	0.7, 1.0, 1.5, and 2.0 mg/kg; BIW or TIW	Hb increase ≥ 1 g/dL from baseline: 30%, 60%, 80% and 100% in 0.7, 1.0, 1.5 and 2.0 mg/ml Roxadustat treatment BIW, respectively; 58%, 40%, 91% and 100% in 0.7, 1.0, 1.5 and 2.0 mg/ml Roxadustat treatment TIW, respectively; 13% in placebo arm
NCT01147666[35]	US; patients with stable end-stage renal disease (ESRD) treated with haemodialysis and previously with Epoetin Alfa to maintain Hb levels	Phase 2; 6 weeks and 19 weeks; randomized, open-label, active comparator (Epoetin Alfa), placebo-controlled	161	Part 1: 6-week treatment of weight-based starting dose 1.0, 1.5, 1.8, or 2 mg/kg TIW<br>Part 2: 19-week treatment (refined by part 1) o 1.3, 1.8, 2.0 mg/kg or weight-tiered treatment: 70-100-150(low)/70-120-200(high) mg TIW	Part 1- Hb increase ≥ -0.5 g/dL from baseline: 79% of subjects in Roxadustat 1.5-2.0 mg/kg pool, versus 33% subjects in Epoetin Alfa arm<br>Part 2- mean Hb ≥ 11.0 g/dL during last 4 weeks of treatment: average Roxadustat for Hb level maintenance 1.68 ± 0.65 mg/kg TIW
NCT01244763[36]	US; patients with NDD CKD (stage 3 or 4) and Hb ≤ 10.5 g/dL	Phase 2b; 16 or 24 weeks; randomized, open label	145	16 weeks: weight-tiered treatment starting dose: 60-100-140 mg TIW (to BIW[a])<br>24 weeks: weight-tiered treatment starting dose: 70-100-150 mg BIW (to QW when Hb response was reached) or 50, 100, and 70 mg fixed starting dose (TIW to BIW[a] to	Hb increase ≥ 1 g/dL from baseline and Hb of ≥ 11.0 g/dL (Hb response) by week 17 after 16 weeks of treatment: achieved in 91.6% of 143 subjects (evaluable for efficacy);<br>median times to Hb response shorter in higher weekly starting dose cohorts

				QW[a])<br>Max dose capped at 2.2/2.5 mg/kg	
NCT01414075[37]	US, Hong Kong, Russia; patients with ESRD and Hb ≤ 10.0 g/dL	Phase 2; 12 weeks; randomized, open-label	60	Weight-tiered treatment starting dose: 60-100-140 (low)/100-140-200(medium)/140-200-300(high) mg/kg (max 2.5 mg/kg) TIW	Hb increase ≥ 1 g/dL (Hb response) from baseline: achieved in 96% of all Roxadustat treated subjects in 12 weeks;<br>median time to Hb response: 3 weeks in each cohort
NCT01599507[38]	China; patients with NDD CKD and Hb < 10.0 g/dL	Phase 2; 9 weeks; randomized, double-blind, placebo-controlled	91	Weight-tiered treatment starting dose: 70-90-120(low)/90-120-150(high) mg/kg TIW	Hb increase ≥ 1g/dL from baseline: 80% achieved in the low-dose cohort, 87.1% in the high-dose cohort, versus 23.3% in the placebo arm
NCT01596855[38]	China; patients with ESRD on haemodialysis and previously with Epoetin Alfa (7 weeks prior to randomization)	Phase 2; 7 weeks; randomized, open-label, active comparator (Epoetin Alfa)	87	Weight-tiered treatment starting dose: 70-90-120(low)/90-120-150(medium)/90-140-180(high) mg/kg TIW	Hb levels maintained: 59.1%, 88.9% and 100% in the low-, medium-, and high-dose cohort versus 50% in the Epoetin Alfa-treated group
		**Molidustat (BAY 85-3934)**			
NCT02021370[39]	US, Australia, Bulgaria, France, Germany, Hungary, Israel, Italy, Japan, Republic of Korea, Poland, Romania, Spain, Turkey, and UK; subjects with non-dialysis CKD stage 3-5 and Hb < 10.5 g/dL	Phase 2b; 16 weeks; randomized, double-blind, placebo-controlled	121	25, 50, and 75 mg daily<br>25, and 50 mg BID	All Molidustat doses increased Hb in NDD CKD patients in a dose-dependent fashion of at least 1.14 g/dL, compared to 0.09 g/dL increase with placebo treatment.
NCT02021409[40]	Australia, Bulgaria, France, Germany, Hungary, Israel, Italy, Japan, Republic of Korea, Poland, Romania, Spain, Turkey, and UK; pre-dialysis subjects already on Darbepoetin treatment	Phase 2b; 16 weeks; randomized; open-label, active comparator (Darbepoetin Alfa)	124	Starting doses: 25, 50, and 75 mg daily;<br>dose adjustment based on patient Hb levels and tolerability ;<br>planned doses; 15, 25, 50, 75, 100, 150 mg daily	Mean change in Hb to evaluation period:<br>Molidustat groups + 0.46 g/dL<br>Darbepoetin Alfa +0.18 g/dL
		**Daprodustat (GSK1278863)**			
NCT01047397[41]	Australia, New Zealand, India, and Russia; patients with NDD CKD stage 3-5 (n=70) and haemodialysis	Phase 2a; 28 days; randomized, single-blind, placebo-controlled, parallel-group	107	Non-dialysis group: starting dose 25, 50, and 100 mg daily<br>Haemodialysis group: starting dose 25 mg daily	Hb increase > 1.0 g/dL: 63% to 91% in non-dialysis group treated with GSK1278863<br>Hb increase > 0.5 g/dL: 71%-89% in

	CKD stage 5D (n=37)			Dose lowered to 10 mg daily in both groups if the Hb response stronger than anticipated at doses ≥ 25mg daily.	haemodialysis group treated with GSK1278863<br>Note: 50 mg, 100 mg groups have high discontinuation rates due to high rate of increase in Hb levels.
NCT01587898[42]	US, Canada, Germany; patients with NDD CKD stage 3-5 and Hb 8.5-11.0 g/dL	Phase 2a; 4 weeks; randomized, double-blind, placebo-controlled, parallel-group	73	0.5, 2, and 5 mg daily	GSK1278863 highest dose (5 mg) treatment increased Hb by ~1.0 g/dL over 4 weeks. Hb responded to GSK1278863 dose-dependently.
NCT01587924[42]	US, Canada, Denmark, Germany, Norway, Sweden; patients with haemodialysis CKD, Hb 9.5-12.0 g/dL, and must be treated with rhEPO for 4 weeks prior to screening	Phase 2a; 4 weeks; randomized, double-blind, active-controlled (rhEPO), parallel-group	83	0.5, 2, and 5 mg daily	5-mg GSK1278863 group maintained Hb level after switched from rhEPO. Low-dose (0.5 and 2 mg) groups did not maintain mean Hb.
NCT02019719[43]	Japan; patients with haemodialysis CKD, Hb 9.5-12.0 g/dL and after 2-8 weeks of ESA discontinuation Hb level should be 8.5-10.5 g/dL before randomization.	Phase 2; 4 weeks; randomized, double-blind, placebo-controlled, parallel-group	97	4, 6, 8, and 10 mg daily	Mean Hb increase from baseline at week 4: -0.28, -0.01, 0.54, and 0.97 g/dL in the 4-, 6-, 8-, and 10-mg Daprodustat group, compared to -1.41 g/dL to a placebo group

*Summarized outcome of primary end point in Hb increase/maintenance.

[a] Dose frequency reduction when Hb response was reached.

Abbreviation: BIW- twice weekly; TIW- three times weekly; QW- once weekly; BID- twice daily; CKD- chronic kidney disease; NDD- non-dialysis dependent; ESA- Erythropoiesis-stimulating agent; Hb- hemoglobin.

Reference

1 K. Thede, I. Flamme, F. Oehme, J. K. Ergüden, F. Stoll, J. Schuhmacher, H. Wild, P. Kolkhof, H. Beck, J. Keldenich, US Patent, 20100305085 A1, 2010.

2 K. Thede, I. Flamme, F. Oehme, J. K. Ergüden, F. Stoll, J. Schuhmacher, H. Wild, P. Kolkhof, H. Beck, J. Keldenich, M. Akbaba, M. Jeske, Patent Application WO, 2008067871 A1, 2008.

3 K. J. Duffy, M. Fitch, J. Jin, R. Liu, A. N. Shaw, K. Wiggall, Patent Application WO, 2007150011 A2, 2007.

4 R. M. Kawamoto, S. Wu, N. C. Warshakoon, A. G. Evdokimov, K. D. Greis, A. S. Boyer, US Patent, 20130203816 A1, 2013.

5 J. A. Aguilar, M. Nilsson, G. Bodenhausen and G. A. Morris, *Chem. Commun.*, 2012, **48**, 811–813.

6 C. Dalvit, P. Pevarello, M. Tato, M. Veronesi, a. Vulpetti and M. Sundstrom, *J. Biomol. NMR*, 2000, **18**, 65–68.

7 R. Chowdhury, I. K. H. Leung, Y.-M. Tian, M. I. Abboud, W. Ge, C. Domene, F.-X. Cantrelle, I. Landrieu, A. P. Hardy, C. W. Pugh, P. J. Ratcliffe, T. D. W. Claridge and C. J. Schofield, *Nat. Commun.*, 2016, **7**, 12673.

8 A. Enthart, J. C. Freudenberger, J. Furrer, H. Kessler and B. Luy, *J. Magn. Reson.*, 2008, **192**, 314–22.

9 I. K. H. Leung, M. Demetriades, A. P. Hardy, C. Lejeune, T. J. Smart, A. Szöllössi, A. Kawamura, C. J. Schofield and T. D. W. Claridge, *J. Med. Chem.*, 2013, **56**, 547–55.

10 I. K. H. Leung, E. Flashman, K. K. Yeoh, C. J. Schofield and T. D. W. Claridge, *J. Med. Chem.*, 2010, **53**, 867–75.

11 S. Horita, J. S. Scotti, C. Thinnes, Y. S. Mottaghi-Taromsari, A. Thalhammer, W. Ge, W. Aik, C. Loenarz, C. J. Schofield and M. a McDonough, *Structure*, 2015, **23**, 639–52.

12 R. Chowdhury, K. K. Yeoh, Y.-M. Tian, L. Hillringhaus, E. A. Bagg, N. R. Rose, I. K. H. Leung, X. S. Li, E. C. Y. Woon, M. Yang, M. A. McDonough, O. N. King, I. J. Clifton, R. J. Klose, T. D. W. Claridge, P. J. Ratcliffe, C. J. Schofield and A. Kawamura, *EMBO Rep.*, 2011, **12**, 463–9.

13 C. Loenarz, R. Sekirnik, A. Thalhammer, W. Ge, E. Spivakovsky, M. M. Mackeen, M. A. McDonough, M. E. Cockman, B. M. Kessler, P. J. Ratcliffe, A. Wolf and C. J. Schofield, *Proc. Natl. Acad. Sci. U. S. A.*, 2014, **111**, 4019–24.

14 A. P. Hardy, I. Prokes, L. Kelly, I. D. Campbell and C. J. Schofield, *J. Mol. Biol.*, 2009, **392**, 994–1006.

15 S.-H. Lee, Jeong Hee Moon, Eun Ah Cho, S.-E. Ryu and Myung Kyu Lee, *J. Biomol. Screen.*, 2008, **13**, 494–503.

16 R. J. Appelhoff, Y.-M. Tian, R. R. Raval, H. Turley, A. L. Harris, C. W. Pugh, P. J. Ratcliffe and J. M. Gleadle, *J. Biol. Chem.*, 2004, **279**, 38458–65.

17 Y.-M. Tian, K. K. Yeoh, M. K. Lee, T. Eriksson, B. M. Kessler, H. B. Kramer, M. J. Edelmann, C. Willam, C. W. Pugh, C. J. Schofield and P. J. Ratcliffe, *J. Biol. Chem.*, 2011, **286**, 13041–51.

18 K. J. Livak and T. D. Schmittgen, *Methods*, 2001, **25**, 402–408.

19 M. A. McDonough, L. A. McNeill, M. Tilliet, C. A. Papamicaël, Q.-Y. Chen, B. Banerji, K. S. Hewitson and C. J. Schofield, *J. Am. Chem. Soc.*, 2005, **127**, 7680–1.

20 N. R. Rose, E. C. Y. Woon, A. Tumber, L. J. Walport, R. Chowdhury, X. S. Li, O. N. F. King, C. Lejeune, S. S. Ng, T. Krojer, M. C. Chan, A. M. Rydzik, R. J. Hopkinson, K. H. Che, M. Daniel, C. Strain-Damerell, C. Gileadi, G. Kochan, I. K. H. Leung, J.

Dunford, K. K. Yeoh, P. J. Ratcliffe, N. Burgess-Brown, F. von Delft, S. Muller, B. Marsden, P. E. Brennan, M. A. McDonough, U. Oppermann, R. J. Klose, C. J. Schofield and A. Kawamura, *J. Med. Chem.*, 2012, **55**, 6639–43.
21 N. Pearce, A. R. Bradley, P. Collins, T. Krojer, R. Nowak, R. Talon, B. D. Marsden, S. Kelm, J. Shi, C. Deane and F. von Delft, *bioRxiv*, 2016.
22 J. M. Elkins, K. S. Hewitson, L. A. McNeill, J. F. Seibel, I. Schlemminger, C. W. Pugh, P. J. Ratcliffe and C. J. Schofield, *J. Biol. Chem.*, 2003, **278**, 1802–6.
23 R. Chowdhury, M. A. McDonough, J. Mecinović, C. Loenarz, E. Flashman, K. S. Hewitson, C. Domene and C. J. Schofield, *Structure*, 2009, **17**, 981–9.
24 W. S. Aik, R. Chowdhury, I. J. Clifton, R. J. Hopkinson, T. Leissing, M. A. McDonough, R. Nowak, C. J. Schofield and L. J. Walport, in *2-Oxoglutarate-Dependent Oxygenases*, eds. R. Hausinger and C. Schofield, The Royal Society of Chemistry, Cambridge, 2015, pp. 59–94.
25 M. C. Chan, N. E. Ilott, J. Schödel, D. Sims, A. Tumber, K. Lippl, D. R. Mole, C. W. Pugh, P. J. Ratcliffe, C. P. Ponting and C. J. Schofield, *J. Biol. Chem.*, 2016, **291**, 20661–73.
26 T. G. G. Battye, L. Kontogiannis, O. Johnson, H. R. Powell and A. G. W. Leslie, *Acta Crystallogr. D. Biol. Crystallogr.*, 2011, **67**, 271–81.
27 M. D. Winn, C. C. Ballard, K. D. Cowtan, E. J. Dodson, P. Emsley, P. R. Evans, R. M. Keegan, E. B. Krissinel, A. G. W. Leslie, A. McCoy, S. J. McNicholas, G. N. Murshudov, N. S. Pannu, E. A. Potterton, H. R. Powell, R. J. Read, A. Vagin and K. S. Wilson, *Acta Crystallogr. D. Biol. Crystallogr.*, 2011, **67**, 235–42.
28 Z. Otwinowski and W. Minor, *Methods Enzymol.*, 1997, **276**, 307–326.
29 G. N. Murshudov, A. A. Vagin and E. J. Dodson, *Acta Crystallogr. D. Biol. Crystallogr.*, 1997, **53**, 240–55.
30 P. D. Adams, P. V Afonine, G. Bunkóczi, V. B. Chen, I. W. Davis, N. Echols, J. J. Headd, L.-W. Hung, G. J. Kapral, R. W. Grosse-Kunstleve, A. J. McCoy, N. W. Moriarty, R. Oeffner, R. J. Read, D. C. Richardson, J. S. Richardson, T. C. Terwilliger and P. H. Zwart, *Acta Crystallogr. D. Biol. Crystallogr.*, 2010, **66**, 213–21.
31 A. T. Brünger, P. D. Adams, G. M. Clore, W. L. DeLano, P. Gros, R. W. Grosse-Kunstleve, J. S. Jiang, J. Kuszewski, M. Nilges, N. S. Pannu, R. J. Read, L. M. Rice, T. Simonson and G. L. Warren, *Acta Crystallogr. D. Biol. Crystallogr.*, 1998, **54**, 905–21.
32 E. R. Martin, M. T. Smith, B. J. Maroni, Q. C. Zuraw and E. M. DeGoma, *Am. J. Nephrol.*, 2017, **45**, 380–388.
33 P. E. Pergola, B. S. Spinowitz, C. S. Hartman, B. J. Maroni and V. H. Haase, *Kidney Int.*, 2016, **90**, 1115–1122.
34 A. Besarab, R. Provenzano, J. Hertel, R. Zabaneh, S. J. Klaus, T. Lee, R. Leong, S. Hemmerich, K.-H. P. Yu and T. B. Neff, *Nephrol. Dial. Transplant.*, 2015, **30**, 1665–1673.
35 R. Provenzano, A. Besarab, S. Wright, S. Dua, S. Zeig, P. Nguyen, L. Poole, K. G. Saikali, G. Saha, S. Hemmerich, L. Szczech, K. H. P. Yu and T. B. Neff, *Am. J. Kidney Dis.*, 2016, **67**, 912–924.
36 R. Provenzano, A. Besarab, C. H. Sun, S. A. Diamond, J. H. Durham, J. L. Cangiano, J. R. Aiello, J. E. Novak, T. Lee, R. Leong, B. K. Roberts, K. G. Saikali, S. Hemmerich, L. A. Szczech, K.-H. P. Yu and T. B. Neff, *Clin. J. Am. Soc. Nephrol.*, 2016, **11**, 982–991.
37 A. Besarab, E. Chernyavskaya, I. Motylev, E. Shutov, L. M. Kumbar, K. Gurevich, D. T. M. Chan, R. Leong, L. Poole, M. Zhong, K. G. Saikali, M. Franco, S. Hemmerich, K.-H. P. Yu and T. B. Neff, *J. Am. Soc. Nephrol.*, 2016, **27**, 1225–33.

38 N. Chen, J. Qian, J. Chen, X. Yu, C. Mei, C. Hao, G. Jiang, H. Lin, X. Zhang, L. Zuo, Q. He, P. Fu, X. Li, D. Ni, S. Hemmerich, C. Liu, L. Szczech, A. Besarab, T. B. Neff, K. H. Peony Yu and F. H. Valone, *Nephrol. Dial. Transplant.*, 2017, **32**, 1373–1386.
39 I. C. Macdougall, T. Akizawa, J. Berns, S. Lentini and T. Bernhardt, *Nephrol. Dial. Transplant.*, 2016, **31**, i16–i16.
40 I. C. Macdougall, T. Akizawa, J. Berns, S. Lentini, T. Bernhardt and T. Krüger, *Nephrol. Dial. Transplant.*, 2016, **31**, i193–i193.
41 R. A. Brigandi, B. Johnson, C. Oei, M. Westerman, G. Olbina, J. de Zoysa, S. D. Roger, M. Sahay, N. Cross, L. McMahon and V. Guptha, *Am. J. Kidney Dis.*, 2016, **67**, 861–871.
42 L. Holdstock, A. M. Meadowcroft, R. Maier, B. M. Johnson, D. Jones, A. Rastogi, S. Zeig, J. J. Lepore and A. R. Cobitz, *J. Am. Soc. Nephrol.*, 2016, **27**, 1234–1244.
43 T. Akizawa, Y. Tsubakihara, M. Nangaku, Y. Endo, H. Nakajima, T. Kohno, Y. Imai, N. Kawase, K. Hara, J. Lepore and A. Cobitz, *Am. J. Nephrol.*, 2017, **45**, 127–135.
